# A New Way to 2,3,4-Trisubstituted Benzo[*h*]quinolines: Synthesis, Consecutive Reactions and Cellular Activities †

**DOI:** 10.3390/molecules28062479

**Published:** 2023-03-08

**Authors:** Viktor A. Zapol’skii, Sandra Kaul, Bianka Karge, Mark Brönstrup, Mimoza Gjikaj, Dieter E. Kaufmann

**Affiliations:** 1 Institute of Organic Chemistry, Clausthal University of Technology, Leibnizstraße 6, 38678 Clausthal-Zellerfeld, Germany; 2 Department of Chemical Biology, Helmholtz Centre for Infection Research, Inhoffenstrasse 7, 38124 Braunschweig, Germany; 3 Institute of Inorganic and Analytical Chemistry, Clausthal University of Technology, Paul-Ernst-Str. 4, 38678 Clausthal-Zellerfeld, Germany

**Keywords:** synthetic methods, 2-nitroperchlorobutadiene, benzoquinolines, cyclization, amines, sulfides, nucleophilic substitution, oxidation, medicinal chemistry

## Abstract

The reaction of mercaptoacetic acid esters with pentachloro-2-nitro-1,3-butadiene provides the appropriate precursors for the synthesis of 2,3,4-trisubstituted benzo[*h*]quinolines. These heterocycles are easily accessible via a single-step reaction with naphthalen-1-amine or anthracen-1-amine as the precursor. Due to the steric bulk and high electron density ring, the ring closure of benzo[*h*]quinolines takes place exclusively. Such highly substituted annelated pyridine systems can be modified in subsequent, selective reactions to build up new *N*-heterocycles with promising microbiological properties. The antibacterial and antiproliferative assays against four mammalian cell lines demonstrate that some of the sulfur-substituted benzo[*h*]quinoline analogs display potent phenotypic bioactivities in the single-digit micromolar range.

## 1. Introduction

**Figure 1:**
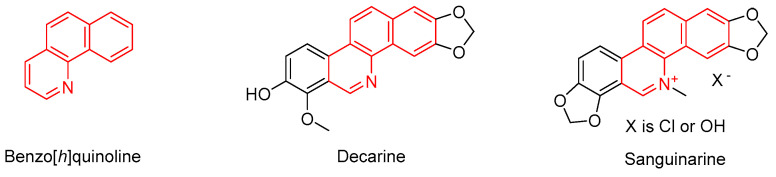
Structures of benzo[*h*]quinoline and two of its natural derivatives.

Previous articles in the field of polyhalogenated nitrobutadienes have already demonstrated the enormous potential of pentachloro-2-nitro-1,3-butadiene (**1**) as a precursor for the “click synthesis” of highly functionalized (hetero)cyclic, as well as acyclic, compounds [1,2]. The corresponding syntheses that we have developed to date always start with the attack of an appropriate nucleophile at the activated terminal carbon atom of the nitrodichlorovinyl group within **1** to undergo a vinylic substitution reaction. Thus, in the case of sulfur nucleophiles, the corresponding thioperchlorobutadiene derivatives are easily accessible [3,4]. In this paper, we describe the unexpected reaction of thioperchloronitrobutadienes to 2,3,4-trisubstituted benzo[*h*]quinolines, which are tricyclic azaheterocycles with an unusual substitution pattern. Benzo[*h*]quinolines are important natural products, which are isolated from the stem wood of *Zanthoxylum nitidum* [5] or from the roots of *Zanthoxylum capense* [6]. Alkaloid decarine (Figure 1) shows high antibacterial activity against mycobacterial, Gram-positive, and Gram-negative bacteria, and low cytotoxicity toward human macrophages [7]. Sanguinarine (Figure 1) is one of the most examined members in the class of natural 3,4-disubstituted benzo[*h*]quinoline compounds and has multiple application possibilities due to its broad scale of bioactivities, such as antibacterial activities [8], inhibition of enzyme activities [9], prevention and treatment of cancer [10], and mono-nucleosome-binding affinities [11]. In the field of plant protection, sanguinarine was shown to improve the environmental stress resistance of a plant [12]. A panel of fluoro-substituted benzo[*h*]quinolines as inhibitors of tolbutamide hydroxylation was used for the phenotype analysis of human cytochrome P450 2C9 polymorphism [13]. Benzo[*h*]quinoline itself (Figure 1) has been suggested as part of an insecticidal composition for controlling stored product insects [14]. Additionally, the parent azatricyclic system has been proposed as a starting reagent for the synthesis of osmium and ruthenium complexes containing an *N*-heterocyclic carbene ligand [15].

These selected applications, in combination with the unique substitution pattern of the novel benzo[*h*]chinolines, presented herein indicate that it is promising to continue this field of medicinal chemistry.

**Scheme 1:**
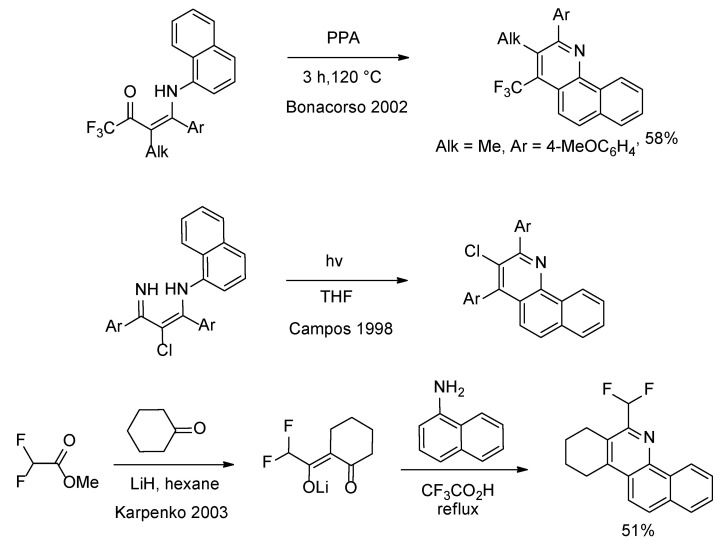
Three synthetic ways to synthesize 2,3,4-trisubsituted benzo[*h*]quinolines [16,17,18].

Until now, it is proven possible to synthesize 2,3,4-trisubsituted benzo[*h*]quinolines, to our best knowledge, by using three ways (Scheme 1):

1. Intramolecular cyclization of *N*-(2-alkyl-1-aryl-3-oxo-4,4,4-trifluoro-1-butenyl)-1-naphthylamines using polyphosphoric acid (PPA) at 120 °C leads to 3-alkyl-2-phenyl-4-(trifluoromethyl)benzo[*h*]quinoline derivatives [16].

2. Electrocyclization of 3-(naphthylamino)-2-alkene imines triggered by ultraviolet light leads to the regioselective synthesis of substituted benzo[*h*]quinolines, giving good to high yields [17].

3. Heterocyclization of naphthalen-1-amine with lithium enolates of 2-(fluoroacetyl)cycloalkanones in refluxing trifluoroacetic acid leads to fluoromethylbenzo[*h*]cyclopenta[*c*]quinolines (yield 51–61%) [18].

A literature review has revealed that many substituted benzo[*h*]quinolines show interesting microbiological properties [7,8,9,10,11,12,13,14]. With our new synthesis starting from 2-nitro-1,3-butadiene derivatives **3a**,**b**, we were able to create uniquely trisubstituted benzo[*h*]quinolines **5a**,**b** at the gram scale. This synthetic route makes it feasible to introduce widely modifiable substituents in a one-step reaction.

## 2. Results

**Figure 2:**
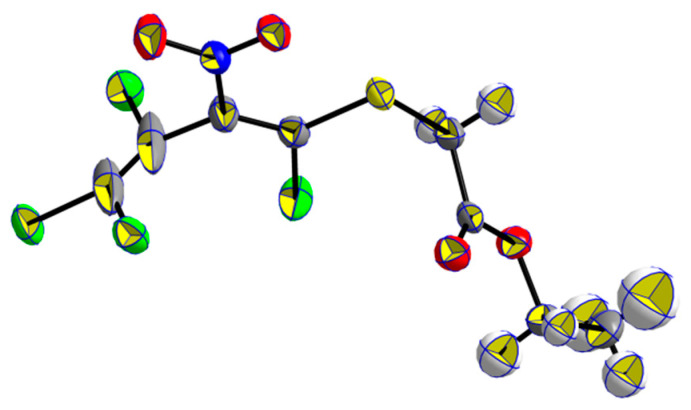
X-ray single-crystal structure of ethyl (*E*)-2-((1,3,4,4-tetrachloro-2-nitrobuta-1,3-dien-1-yl)thio)acetate (**3a**).

**Scheme 2:**
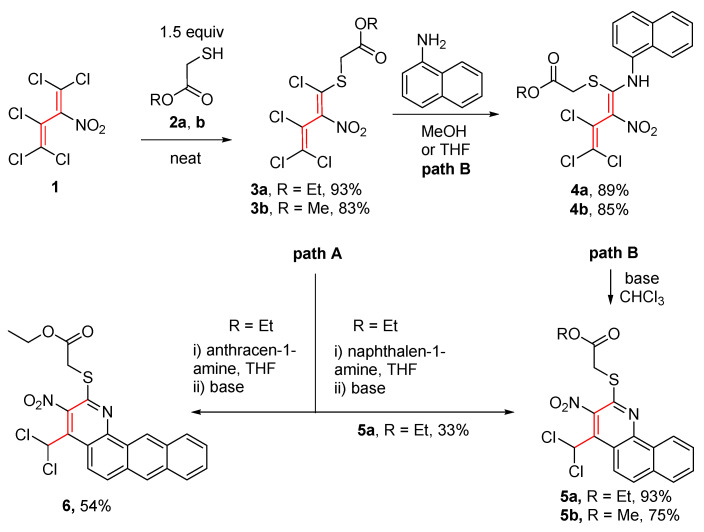
A new way to synthesize 2,3,4-trisubstituted benzo[*h*]quinolines.

The solventless reaction of pentachloro-2-nitro-1,3-butadiene (**1**) with 1.5 equivalents of ethyl 2-mercaptoacetate (**2a**) at room temperature for 14 days furnished ethyl 2-((1,3,4,4-tetrachloro-2-nitrobuta-1,3-dien-1-yl)thio)acetate (**3a**) as a single isomer at a yield of 93% yield, and additionally, the same procedure led to methyl ester **3b** at a yield of 83% (Scheme 2). The *E*-configuration of the sulfanyl-substituted C=C bond could be proven by the X-ray analysis of a single crystal of thiodiene **3a** (Figure 2).

Products **3a**,**b** are versatile synthetic building blocks. Base-promoted reaction of **3a**,**b** with naphthalen-1-amine and subsequent cyclization led to benzo[*h*]quinolines **5**,**6** (Scheme 2).

**Scheme 3:**
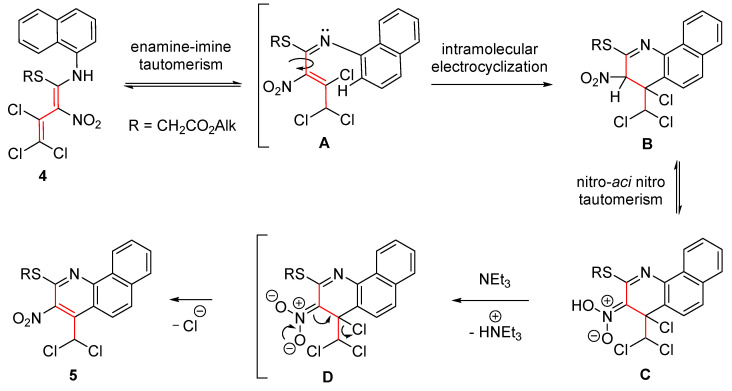
Assumed mechanism for the formation of benzo[*h*]quinoline **5**.

A conceivable mechanism for the cascade reaction leading to the benzo[*h*]quinoline **5** is presented in Scheme 3. The starting material **4** exists exclusively as an *E*-isomer, probably due to a strong hydrogen bond between the NH and NO_2_ groups. Initially, (*E*,*E*)-imine **A** is formed from diene **4** upon enamine–imine tautomerization. Due to an almost free rotation around the central single bond of the side chain, an intramolecular electrocyclization of **A** to give the intermediate **B** as part of an equilibrium appears feasible. Subsequently, dihydropyridine **B** is aromatized by the elimination of HCl in the presence of triethylamine, giving benzo[*h*]quinoline **5**. A Friedel–Crafts substitution or a Michael addition appears less likely under the applied reaction conditions.

**Figure 3:**
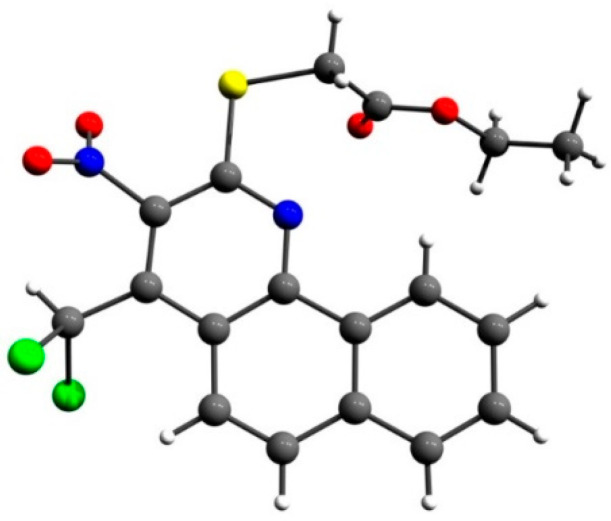
X-ray single-crystal structure of ethyl 2-((4-(dichloromethyl)-3-nitrobenzo[*h*]quinolin-2-yl)thio)acetate (**5a**).

The structure of benzo[*h*]quinoline **5a** was also confirmed by an X-ray single-crystal analysis (Figure 3).

The key step of the reaction path to form benzo[*h*]quinolines **5** is a cyclization reaction. There are two possible ways to obtain product **5**. The first one is a one-pot reaction of mercaptoacetate **3**, naphthalene-1-amine, and triethylamine as a base in THF with up to 33% yield (path **A**, Scheme 2). Following the synthetic route **A,** it also proves to be possible to synthesize naphtho[*h*]quinoline **6** at a 54% yield. To improve the yield, we varied the base (triethylamine, *N*,*N*-dimethylaniline, and pyridine, without a base), solvent (THF, methanol, and chloroform), reaction temperature, and time. Benzo[*h*]quinolines were formed at moderate yields when triethylamine or *N*,*N*-dimethylaniline was used as a base. Without a base, the intermediate product **4** could be isolated at yields up to 89%. The interaction of isolated **4** in a subsequent reaction with triethylamine (path **B**) led to the formation of **5** at yields up to 93%. Therefore, it appeared advantageous to carry out the reaction in a two-step mode (Scheme 2).

**Scheme 4:**
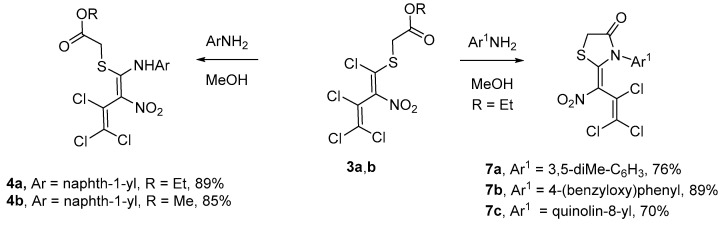
Synthetic pathways to aminothiodienes **4a,b** and thiazolidinones **7a**–**c**.

Since path **B** had been found to be the more efficient one, an attempt was made to synthesize more intermediate products under these reaction conditions. A variety of aromatic amines were selected (Scheme 4).

Interestingly, conversion of **3** with a one-ring system, such as 3,5-dimethylaniline or 4-(benzyloxy)aniline in methanol, afforded (*Z*)-thiazolidinones **7a** and **7b** at a yield of 76% and 89%, respectively, which is similar to the literature [3], and not the open-chain product **4**. Quinoline-8-amine produced (*Z*)-thiazolidinone **7c** as two atropisomers (1:0.75, ^1^H-NMR) at a 70% yield. This phenomenon is due to a less hindered rotation of the C–N bond of the former quinoline-8-amine unit. Unfortunately, there were no conversions to the desired product in the case of 5-aminonaphthalene-2-sulfonic acid, naphthalene-1,8-diamine, naphthalene-1,5-diamine, and anthracen-1-amine.

**Scheme 5:**
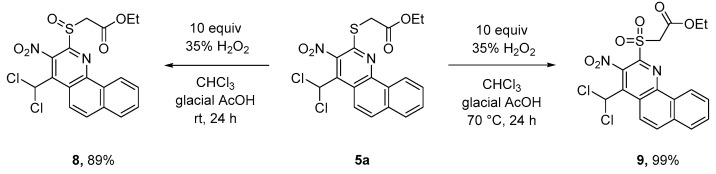
Oxidation of sulfide **5a** to sulfoxide **8** and sulfone **9**.

The original substituents of benzo[*h*]quinolines **5** allow their selective transformation into other functional groups, too. For example, the sulfoxide group of **8** is a good leaving group and can therefore be used in S_N_ reactions, probably proceeding via an addition–elimination mechanism. This way, both *S*- (sulfide **10a**–**d**) and *N*-substituents (amines **11a**,**b**) can be introduced. Access to sulfoxide **8** is gained by the selective oxidation of **5a** with 35% of hydrogen peroxide, and this subsequently leads to sulfone **9** in very good yields. The reaction steps are temperature controlled (Scheme 5).

**Scheme 6:**
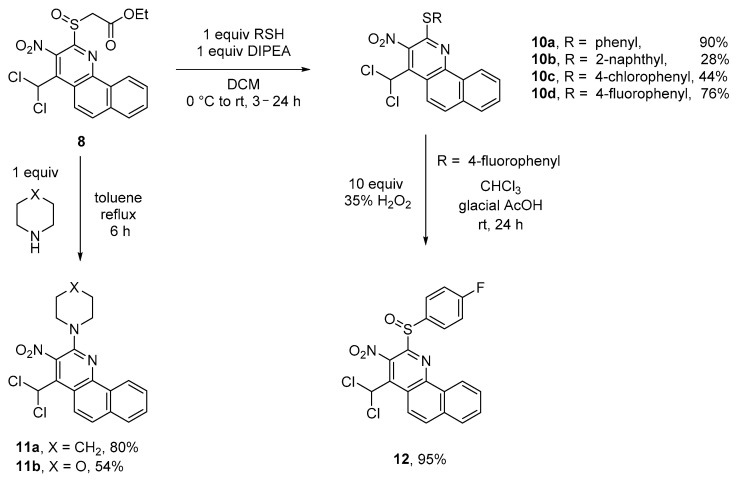
Substitution reactions of sulfoxide **8** with *S*- and *N*-nucleophiles.

The selective substitution reactions of the sulfoxide group of **8** proceeded well with different thiophenolates and morpholine (Scheme 6). The nucleophilicity of alkoholates proved to be too low for an S_N_ reaction.

**Figure 4:**
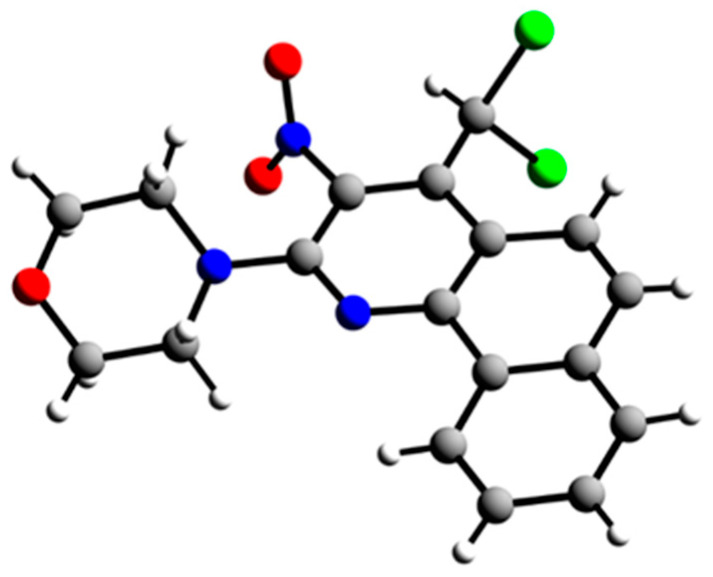
X-ray single-crystal structure of 4-(4-(dichloromethyl)-3-nitrobenzo[*h*]quinolin-2-yl)morpholine (**11b**).

In the case of quinoline **11b,** we were able to carry out an X-ray single-crystal analysis to prove the structural conclusions that we had drawn from the NMR spectra (see Figure 4).

**Scheme 7:**
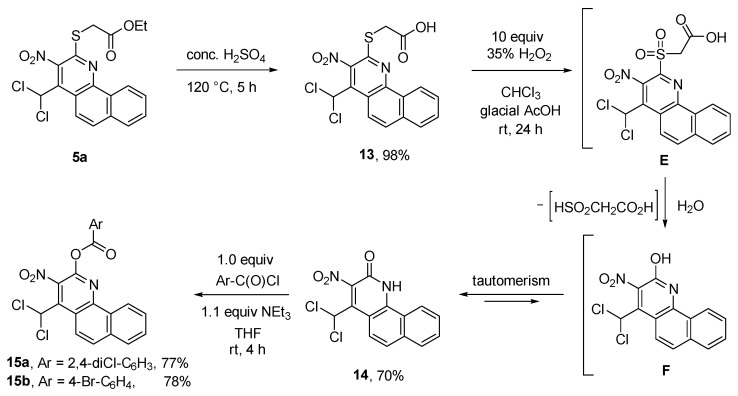
Chemical transformations of sulfide **5a** to acid **13**, lactam **14,** and ester **15a**,**b**.

Another method to modify compound **5a** is the almost quantitative acidic cleavage of its ester group to 2-((4-(dichloromethyl)-3-nitrobenzo[*h*]quinolin-2-yl)thio)acetic acid (**13**). This molecule deserves interest because of its high activity against methicillin-resistant *Staphylococcus aureus* (MRSA) in microbiological tests. The oxidation of acid **13** with hydrogen peroxide furnished lactam **14**. The expected sulfone **E** (Scheme 7), in contrast to sulfone **9** (Scheme 5), was not isolable. We assume that the primarily formed **E** was hydrolyzed instead under the reaction conditions to give quinolin-2-ol **F**. The tautomerization of the intermediate **F** led to lactam **14** at a 70% yield. The esterification of **14** with benzoyl chlorides gave **15a** and **15b** at a 77% and 78% yield, respectively.

**Scheme 8:**
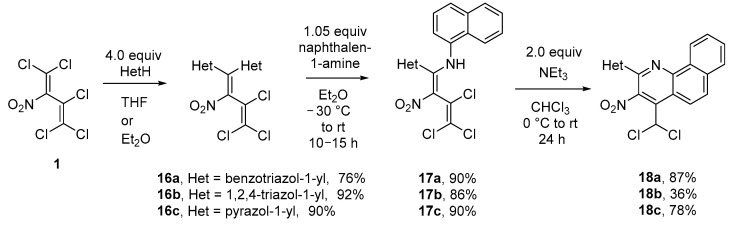
Formation of benzo[*h*]quinolines **18a**–**c** starting from nitrobutadiene **1**.

Additionally, a convenient way to synthesize 2-substituted azolylbenzoquinolines in three steps, starting from nitrodiene **1,** had been developed. At first, diene **1** was reacted with azoles, such as benzotriazole, 1,2,4-triazole, and pyrazole, leading to the formation of 1,1-diazolyl-2-nitrotrichlorobutadienes **16a**–**c** in good yields (76–92%). The transamination of compound **16** with an equimolar amount of naphthalen-1-amine ran smoothly in ether at −30 °C and then r.t., furnishing 1-azolyl-1-(naphthylamino)diene **17** (86–90%). The treatment of butadienes **17** with triethylamine as a base in CHCl_3_ again led to the formation of 2,3,4-trisubstituted benzo[*h*]quinolines **18**, at 36–87% yield (Scheme 8). A change of solvent (CH_2_Cl_2_, DMSO, MeOH, or Et_2_O) and base (NaOH or NaHCO_3_) decreased the yields of quinoline **18a** to 9–55%.

Because of the diverse bioactivities reported for benzo[*h*]quinolines before, a selection of the newly prepared analogs shown above was made to represent all key structural variants. These selected analogs were characterized using phenotypic cellular assays. Their antibiotic activities were measured by using growth inhibition assays against one Gram-positive pathogen, i.e., methicillin-resistant *Staphylococcus aureus* (MRSA), and two Gram-negative pathogens, i.e., *Escherichia coli* and *Pseudomonas aeruginosa*. None of the compounds could inhibit the Gram-negative strains at a concentration of 50 µM, but some analogs were active against MRSA (Table 1). Notably, compounds **8**, **12** and **13** prevented the growth of MRSA with the minimal inhibitory concentrations (MICs) of 17.5, 1.2, and 8.4 µM (corresponding to 7.7, 0.5, and 3.3 µg/mL), respectively. Thus, **12** was the most potent antibiotic among all tested analogs. Interestingly, the formal reduction of sulfoxide in **12** to a sulfide, as in **10d**, led to a complete loss of activity against MRSA. The importance of the naphth-1-yl residue in **8** was illustrated by the fact that its replacement by an anthracen-1-yl moiety, as present in **6**, also led to an inactive compound. Additionally, the sulfur substituent was crucial for activity because nitrogen-substituted benzo[*h*]quinolines, such as the amines **11a** and **11b**, or the triazols and pyrazol **18a**–**18c**, were inactive. Additionally, the tested non-cyclized precursors, such as **16c** and **17a**–**17c**, had no activity.

In addition to probing effects on bacterial pathogens, the compounds’ ability to interfere with the proliferation and/or viability of four mammalian cell lines was assessed by quantifying their mitochondrial dehydrogenase activity in a colorimetric assay with the tetrazolium dye WST-1. For this purpose, the cells were exposed to the compounds at varying concentrations for a time period of 5 days (for L929, KB-3-1, and MCF-7 cells) or 24 h (for FS4-LTM cells). For **8** and **13**, similar activity trends were observed against mammalian cell lines: The sulfoxide **8** was most potent and inhibited the proliferation of all four cell lines with EC_50′_s of 2.5–3.8 µM. Compound **13**, which differs from **8** by the hydrolysis of ester to a carboxylic acid and the oxidation of sulfide to a sulfoxide, had weaker activities with EC_50′_s of 6.9–45 µM. In contrast, the aliphatic substituent of sulfoxide in **8** could be replaced by an aromatic *p*-fluorophenyl group, because **12** inhibited the proliferation of L929, KB-3-1, and FS4-LTM cells with EC_50′_s of 3.3, 3.1, and 4.4 µM, respectively. Compound **10d**, which differs from **12** by the oxidation state at sulfur, displayed significantly reduced activities in L929 and FS4-LTM cells, but it was more potent in KB-3-1 cells.

In summary, the phenotypic cellular assays demonstrate that a distinct subset of the benzo[*h*]quinolines synthesized in this study possess high bioactivities against mammalian cell lines and the bacterial pathogen MRSA.

## 3. Materials and Methods

### 3.1. General Remarks

The solvents and reagents were used as received from commercial sources without further purification. The TLC was performed with Merck aluminum-backed TLC plates using silica gel 60, F_254_. A flash column chromatography was performed using Macherey–Nagel silica gel 60 M (0.040–0.063 mm) with appropriate mixtures of petroleum ether (PE, boiling range 60–70 °C) and ethyl acetate as the eluents. The melting points were determined in capillary tubes with a Büchi B-520. The FTIR spectra were recorded with a Bruker “Alpha-T” spectrometer (Bruker, Ettlingen, Germany) with the solid compounds measured as KBr pellets. The ATR-IR spectra were measured on the same instrument with a Bruker “Alpha Platinum ATR” single-reflection diamond ATR module. The ^1^H NMR and ^13^C NMR spectra at 600 and 150 MHz, respectively, were recorded with an “Avance III” 600 MHz FTNMR spectrometer (Bruker, Rheinstetten, Germany). The ^1^H NMR and ^13^C NMR spectra at 400 and 100 MHz, respectively, were recorded with an “Avance” 400 MHz FT-NMR spectrometer (also Bruker). The ^1^H and ^13^C NMR spectra were examined with reference to the residual solvent peak: CDCl_3_ at δ 7.26 (^1^H) and 77.0 ppm (^13^C), and DMSO-*d_6_* at δ 2.50 (^1^H) and 39.7 ppm (^13^C). The NMR spectra and HR-MS data of the newly synthesized compounds are available in the Supplementary Materials. The mass spectra were obtained with a Hewlett–Packard MS 5989B spectrometer (HP Inc., Palmer, MA, USA), usually in a direct mode with the electron impact (70 eV). For the chlorinated and brominated compounds, all peak values of molecular ions and fragments refer to the isotope ^35^Cl. High-resolution mass spectra were recorded with the “Impact II” from BRUKER (Bruker Daltonik GmbH, Bremen, Germany).

*Pentachloro-2-nitro-1,3-butadiene* (**1**). The product was prepared from 2*H*–pentachloro-1,3-butadiene at a 53% yield (b.p. 69–71 °C/1 mbar), according to the literature [19].

*Ethyl (E)-2-((1,3,4,4-tetrachloro-2-nitrobuta-1,3-dien-1-yl)thio)acetate* (**3a**). The product was prepared according to the published literature [3] from nitrodiene **1** and ethyl 2-mercaptoacetate. The yield was 93%. All spectral data are in accordance with the literature.

*Methyl (E)-2-((1,3,4,4-tetrachloro-2-nitrobuta-1,3-dien-1-yl)thio)acetate* (**3b**). The product was prepared according to the published literature [3] from nitrodiene **1** and methyl 2-mercaptoacetate. The yield was 83%. All spectral data are in accordance with the literature.

*Ethyl (E)-2-((3,4,4-trichloro-1-(naphthalen-1-ylamino)-2-nitrobuta-1,3-dien-1-yl)thio)acetate* (**4a**). A solution of acetate **3a** (5.00 g, 14.0 mmol) and naphthalen-1-amine (4.00 g, 28.0 mmol) in methanol (20 mL) was stirred for 3 h at r.t., and the precipitated product was filtered off and washed with aqueous HCl (18%, 20 mL), water (20 mL), and cold methanol (20 mL). The product was a yellowish solid, at 5.73 g (89%) and m.p. of 118–119 °C. IR (KBr): ν_max_ = 2986, 1741, 1556, 1470, 1393, 1182, and 829 cm^−1^. ^1^H NMR (CDCl_3_, 400 MHz): δ 12.16 (s, 1 H, NH), 8.03–7.93 (m, 3 H), 7.62–7.50 (m, 4 H), 4.98 (q, *J* = 7.1 Hz, 2 H, OCH_2_), 2.95 (s, 2 H, SCH_2_), and 1.12 (t, *J* = 7.1 Hz, 3 H, CH_3_); ^13^C NMR (CDCl_3_, 100 MHz): δ 166.6 (C=O), 158.3 (NCS), 134.2 (Cq), 133.0 (Cq), 131.3 (Cq), 128.8 (CH), 128.7 (CH), 128.2 (CCl), 127.7 (CH), 127.3 (CH), 125.4 (CH), 123.6 (CCl_2_), 122.9 (CH), 122.2 (CNO_2_), 121.5 (CH), 62.1 (OCH_2_), 34.5 (SCH_2_), and 13.9 (CH_3_); EIMS: *m/z* (%) 462 (2) [M + H]^+^, 424 (2) [M − Cl]^+^, 414 (5) [M − NO_2_]^+^, 379 (5) [M − NO_2_ − Cl]^+^, 343 (10) [M − NO_2_ − 2Cl]^+^, 252 (10) [M − NO_2_ − Cl − naphthyl]^+^, 143 (100) [naphthylamine]^+^. HRMS (ESI): *m/z* was calculated for C_18_H_15_Cl_3_N_2_o_4_SNa [M + Na]^+^ 482.9710 and found 482.9720.

*Methyl (E)-2-((3,4,4-trichloro-1-(naphthalen-1-ylamino)-2-nitrobuta-1,3-dien-1-yl)thio)acetate* (**4b**). The product was prepared according to acetate **4a** from acetate **3b** (0.600 g, 1.759 mmol) and naphthalen-1-amine (0.500 g, 3.519 mmol). The product was a yellowish solid, with a yield of 0.668 g (85%) and m.p. of 110 °C. IR (ATR): ν_max_ = 2905, 1745, 1553, 1527, 1390, 1170, and 801 cm^−1^. ^1^H NMR (CDCl_3_, 400 MHz): δ 12.14 (s, 1 H, NH), 8.01 (d, *J* = 7.4 Hz, 1 H), 7.94 (d, *J* = 7.4 Hz, 1 H), 7.89 (d, *J* = 8.0 Hz, 1 H), 7.66–7.60 (m, 3 H), 7.52 (t, *J* = 8.0 Hz, 1 H), 3.53 (s, 3 H, OCH_3_), and 2.97 (s, 1 H, SCH_2_); ^13^C-NMR (CDCl_3_, 100 MHz): δ 167.1 (C=O), 158.1 (NCS), 134.2 (Cq), 133.0 (Cq), 128.8 (Cq), 128.7 (CH), 128.4 (CH), 128.3 (CCl), 127.8 (CH), 127.3 (CH), 125.4 (CH), 123.6 (CCl_2_), 123.0 (CH), 122.4 (CNO_2_), 121.5 (CH), 52.8 (OCH_3_), and 34.5 (SCH_2_); EIMS: *m/z* (%) 447 (25) [M + H]^+^, 351 (20) [M − Cl − COOCH_3_]^+^, 296 (20) [M − NO_2_ − SCH_2_COOCH_3_]^+^, 225 (40) [M − 2Cl − NO_2_ − SCH_2_COOCH_3_]^+^, and 143 (55) [naphthyl]^+^. HRMS (ESI): *m/z* was calculated for C_17_H_13_Cl_3_N_2_O_4_SNa [M + Na]^+^ 468.9554 and found 468.9560.

*Ethyl 2-((4-(dichloromethyl)-3-nitrobenzo[h]quinolin-2-yl)thio)acetate* (**5a**). To a solution of **4a** (2.00 g, 4.3 mmol) in chloroform (20 mL), triethylamine (0.870 g, 0.60 mmol) was added at 0 °C, and the reaction mixture was stirred for 1 d at r.t. The solvent was removed in vacuo and the residue was dissolved in methanol (5 mL). The precipitated product was filtered off with suction and washed with aqueous HCl (18%, 20 mL), water (20 mL), and methanol (30 mL) and then dried in vacuo to give **5a**, yellow solid. The yield was 1.70 g (93%), with m.p. of 171–172 °C. IR (KBr): ν_max_ = 3042, 2989, 1744, 1534, 1334, 831, and 735 cm^−1^. ^1^H NMR (CDCl_3_, 400 MHz): δ 9.16 (d, *J* = 7.2 Hz, 1 H), 8.65 (d, *J* = 9.6 Hz, 1 H), 7.95 (d, *J* = 9.6 Hz, 1 H), 7.94 (d, *J* = 7.2 Hz, 1 H), 7.82–7.74 (m, 2 H), 7.20 (s, 1 H, CHCl_2_), 4.22 (q, *J* = 7.2 Hz, 2 H, OCH_2_), 4.20 (s, 2 H, SCH_2_), and 1.28 (t, *J* = 7.2 Hz, 3 H, CH_3_); ^13^C NMR (CDCl_3_, 100 MHz): δ 168.6 (C=O), 149.0 (Cq), 147.9 (Cq), 139.1 (Cq), 135.7 (Cq), 134.3 (Cq), 130.4 (Cq), 130.3 (CH), 128.8 (CH), 128.0 (CH), 127.9 (CH), 125.7 (CH), 122.3 (CH), 119.6 (Cq), 63.4 (CHCl_2_), 62.1 (OCH_2_), 33.8 (SCH_2_), and 14.2 (CH_3_); EIMS: *m/z* (%) 424 (12) [M]^+^, 379 (5) [M − OEt]^+^, 355 (8) [M − 2Cl]^+^, 303 (25) [M − SCH_2_CO_2_Et]^+^, and 192 (15) [M − 2HCl − SCH_2_CO_2_Et − NO_2_]^+^. HRMS (ESI): *m/z* was calculated for C_18_H_14_Cl_2_N_2_O_4_SNa [M + Na]^+^ 446.9944 and found 446.9947.

*Methyl 2-((4-(dichloromethyl)-3-nitrobenzo[h]quinolin-2-yl)thio)acetate* (**5b**). The product was prepared according to benzo[*h*]quinoline **5a** from acetate **4b** (0.300 g, 0.67 mmol) and triethylamine (0.068 g, 0.126 mmol). The product was a yellowish solid, with a yield of 0.207 g (75%) and m.p. of 263 °C. IR (ATR): ν_max_ = 2951, 1747, 1533, 1331, 827, and 731 cm^−1^. ^1^H NMR (CDCl_3_, 400 MHz): δ 9.13 (d, *J* = 7.2 Hz, 1 H), 8.65 (d, *J* = 9.4 Hz, 1 H), 7.95 (d, *J* = 9.4 Hz, 1 H), 7.94 (d, *J* = 7.2 Hz, 1 H), 7.81–7.75 (m, 2 H), 7.02 (s, 1 H, CHCl_2_), 4.19 (s, 2 H, SCH_2_), and 3.77 (s, 3 H, OCH_3_); ^13^C NMR (CDCl_3_, 100 MHz): δ 169.1 (C=O), 148.9 (Cq), 147.9 (Cq), 139.0 (Cq), 135.8 (Cq), 134.2 (Cq), 130.4 (Cq), 130.3 (CH), 128.8 (CH), 128.1 (CH), 128.0 (CH), 125.5 (CH), 122.2 (CH), 119.6 (Cq), 63.3 (CHCl_2_), 52.8 (OCH_3_), and 33.6 (SCH_2_); EIMS: *m/z* (%) 410 (55) [M]^+^, 304 (40) [M − SCH_2_COOCH_3_]^+^, 260 (15) [M − SCH_2_COOCH_3_ − NO_2_]^+^, and 252 (100) [M − SCH_2_COOCH_3_ − C_4_H_4_]^+^. HRMS (ESI): *m/z* was calculated for C_17_H_12_Cl_2_N_2_O_4_SNa [M + Na]^+^ 432.9787 and found 432.9793.

*Ethyl 2-((4-(dichloromethyl)-3-nitronaphtho[2,3-h]quinolin-2-yl)thio)acetate* (**6**). A solution of acetate **3a** (0.500 g, 1.4 mmol) and 1-aminoanthracene (0.270 g, 1.40 mmol) in anhydrous THF (20 mL) was stirred at r.t. for 16 h. Subsequently, triethylamine (0.141 g, 1.4 mmol) was added, the mixture was stirred at r.t. for 1 d, and the solvent was removed in vacuo. During the addition of methanol (2 mL), a solid precipitated and was filtered off with suction and washed with aqueous HCl (18%, 20 mL), water (20 mL), and cold (methanol (25 mL). The yellowish solid was dried in vacuo. The yield was 0.377 g (54%), with m.p. of 224 °C. IR (ATR): ν_max_ = 1734, 1548, 1533, 1337, 1149, 987 cm^−1^. ^1^H NMR (CDCl_3_, 400 MHz): δ 9.56 (s, 1 H), 8.60 (s, 1 H), 8.38 (d, *J* = 9.5 Hz, 1 H), 8.20–8.16 (m, 3 H), 8.06 (s, 1 H, CHCl_2_), 7.72–7.69 (m, 2 H), 4.45 (s, 2 H, SCH_2_), 4.09 (q, *J* = 7.1 Hz, 2 H, OCH_2_), and 1.06 (t, *J* = 7.1 Hz, 3 H, CH_3_); ^13^C NMR (CDCl_3_, 100 MHz): δ 168.8 (C=O), 149.1 (Cq), 148.4 (Cq), 139.6 (Cq), 135.2 (Cq), 133.5 (Cq), 131.9 (Cq), 130.8 (Cq), 130.0 (CH), 129.0 (CH), 128.2 (CH), 127.9 (CH), 127.4 (Cq), 127.3 (CH), 127.2 (CH), 125.8 (CH), 120.6 (CH), 119.5 (Cq), 64.3 (CHCl_2_), 61.5 (OCH_2_), 33.6 (SCH_2_), and 14.2 (CH_3_); EIMS: *m/z* (%) 474 (77) [M]^+^, 429 (7) [M − OEt]^+^, 386 (10) [M − CH_3_COOEt]^+^, 355 (60) [M − SCH_2_COOEt]^+^, and 302 (100) [M − CH_3_COOEt − CHCl_2_]^+^. HRMS (ESI): *m/z* was calculated for C_22_H_16_Cl_2_N_2_O_4_SNa [M + Na]^+^ 497.0100 and found 497.0106.

*(Z)-3-(3,5-Dimethylphenyl)-2-(2,3,3-trichloro-1-nitroallylidene)thiazolidin-4-one* (**7a**). A mixture of acetate **3a** (0.500 g, 1.4 mmol) and 4-(benzyloxy)aniline (0.350 g, 2.9 mmol) in methanol (2.5 mL) was stirred for 7 d at r.t. while the product was precipitating. It was filtered off with suction, washed with cold methanol (25 mL), and dried in vacuo. The product was a beige solid, with a yield of 0.415 g (76%) and m.p. of 240 °C. IR (ATR): ν_max_ = 2983, 1756, 1595, 1369, 1287, and 679 cm^−1^. ^1^H NMR (DMSO-*d_6_*, 400 MHz): δ 7.14 (s, 1 H), 6.99 (s, 2 H), 4.16 (q, 2 H, *J* = 18.4 Hz, CH_2_), 2.32 (s, 3 H, CH_3_), and 2.30 (s, 3 H, CH_3_); ^13^C NMR (DMSO-d_6_; 100 MHz): δ 173.7 (C=O), 165.7 (SCN), 138.7 (Cq), 138.4 (Cq), 134.0 (Cq), 131.7 (CH), 128.2 (CCl), 126.2 (CH), 124.6 (CH), 121.1 (CNO_2_), 120.9 (CCl_2_), 32.4 (CH_2_), and 20.5 (2C, 2CH_3_); EIMS: *m/z* (%) 391 (3) [M]^+^, 356 (7) [M − Cl]^+^, 264 (30) [M − NO_2_ − CCl_2_]^+^, 247 (100) [M − NO_2_ − 2CH_3_ − 2Cl]^+^, 228 (25) [M − NO_2_ − CCl_2_ − Cl]^+^, 202 (70) [M − CCl_2_ − Ph(CH_3_)_2_]^+^, and 104 (60) [Ph(CH_3_)_2_]^+^. HRMS (ESI): *m/z* was calculated for C_14_H_11_Cl_3_N_2_O_3_SNa [M + Na]^+^ 414.9448 and found 414.9453.

*(Z)-3-(4-(Benzyloxy)phenyl)-2-(2,3,3-trichloro-1-nitroallylidene)thiazolidin-4-one* (**7b**). The product was prepared according to thiazolidin-4-one **7a** from acetate **3a** (1.00 g, 2.80 mmol), 4-(benzyloxy)aniline hydrochloride (1.39 g, 7.02 mmol), and sodium hydroxide (0.28 g, 7.02 mmol) in methanol (10 mL) and stirred for 7 d at r.t. It was a beige solid, with a yield of 1.174 g (89%) and m.p. of 201 °C. IR (ATR): ν_max_ = 2923, 1754, 1523, 1363, 1229, and 743 cm^−1^. ^1^H NMR (DMSO-*d_6_*, 400 MHz): δ 7.46 (d, *J* = 6.8 Hz, 2 H), 7.40 (d, *J* = 6.8 Hz, 1 H), 7.30 (d, *J* = 6.8 Hz, 1 H), 7.33 (t, *J* = 6.8 Hz, 1 H), 7.31 (d, *J* = 7.6 Hz, 2 H), 7.12–7.09 (m, 2 H), 5.17 (q, *J* = 8.2 Hz, 2 H, OCH_2_), and 4.13 (q, *J* = 18.3 Hz, 2 H, SCH_2_); ^13^C NMR (DMSO-*d_6_*, 100 MHz): δ 174.5 (C=O), 166.3 (SCN), 159.8 (Cq), 137.2 (Cq), 130.5 (CH), 129.1 (CH), 128.9 (2C, CH), 128.5 (Cq), 128.4 (CH), 128.1 (2C, CH), 127.4 (CCl), 121.7 (2C, CNO_2_, CCl_2_), 115.7 (CH), 115.6 (CH), 69.9 (OCH_2_), and 32.8 (SCH_2_); EIMS: *m/z* (%) 469 (10) [M]^+^, 435 (20) [M − Cl]^+^, 378 (45) [M − Bz]^+^, 326 (50) [M − OBz − Cl]^+^, and 313 (100) [M − OBz − NO_2_]^+^. HRMS (ESI): *m/z* was calculated for C_19_H_12_Cl_3_N_2_O_4_S [M]^+^ 469.9656 and found 469.9583.

*(Z)-3-(Quinolin-8-yl)-2-(2,3,3-trichloro-1-nitroallylidene)thiazolidin-4-one* (**7c**). The product was synthesized according to thiazolidin-4-one **7a** from acetate **3a** (0.500 g, 1.40 mmol) and quinoline-8-amine (0.60 g, 4.20 mmol) in methanol (10 mL) by stirring for 6 h at r.t. The product was an orange solid in a mixture of two rotamers, with ratio (1: 0.75). The yield was 0.404 g (70%), with m.p. of 197 °C. IR (ATR): ν_max_ = 2978, 1747, 1519, 1374, 1285, and 754 cm^−1^. Major Isomer: ^1^H NMR (DMSO-*d_6_*, 600 MHz): δ 8.92 (dd, *J* = 4.2, 1.6 Hz, 1 H), 8.54 (ddd, *J* = 9.5, 8.2, 1.4 Hz, 1 H), 8.22 (dd, *J* = 8.2, 1.3 Hz, 1 H), 7.95 (dd, *J* = 7.3, 1.3 Hz, 1 H), 7.80 (dd, *J* = 7.4, 4.0 Hz, 1 H), 7.66 (dd, *J* = 8.3, 4.2 Hz, 1 H), and 4.46 (q, *J* = 18.7 Hz, 2 H, CH_2_); ^13^C NMR (DMSO-*d_6_*, 150 MHz): δ 174.0 (C=O), 166.2 (NCS), 151.6 (CH), 142.6 (Cq), 136.8 (CH), 131.2 (Cq), 131.1 (CH), 129.2 (Cq), 129.1 (CH), 127.8 (CCl), 126.2 (CH), 122.8 (CH), 121.3 (CNO_2_), 120.9 (CCl_2_), and 32.1 (CH_2_); Minor Isomer: ^1^H NMR (DMSO-*d_6_*, 600 MHz): δ 8.90 (dd, *J* = 8.2, 1.6 Hz, 1 H), 8.54 (ddd, *J* = 9.5, 8.2, 1.4 Hz, 1 H), 8.18 (dd, *J* = 8.2, 1.3 Hz, 1 H), 8.00 (dd, *J* = 7.3, 1.3 Hz, 1 H), 7.76 (dd, *J* = 7.4, 4.2 Hz, 1 H), 7.66 (dd, *J* = 8.3, 4.2 Hz, 1 H), and 4.35 (q, *J* = 18.7 Hz, 2 H, CH_2_); ^13^C NMR (DMSO-*d_6_*, 150 MHz): δ 174.0 (C=O), 166.8 (NCS), 151.6 (CH), 143.0 (Cq), 136.7 (CH), 131.3 (Cq), 131.2 (CH), 129.3 (Cq), 129.1 (CH), 128.7 (CCl), 126.2 (CH), 122.7 (CH), 121.6 (CNO_2_), 120.2 (CCl_2_), and 32.3 (CH_2_); EIMS: *m/z* (%) 414 (1) [M]^+^, 369 (100) [M − NO_2_]^+^, 299 (15) [M − NO_2_ − 2Cl]^+^, 228 (15) [M − C_3_NO_2_Cl_3_]^+^, and 128 (40) [quinoline]^+^. HRMS (ESI): *m/z* was calculated for C_15_H_7_Cl_3_N_3_O_3_S [M − H]^+^ 413.9279 and found 413.9258.

*Ethyl 2-((4-(dichloromethyl)-3-nitrobenzo[h]quinolin-2-yl)sulfinyl)acetate* (**8**). Acetate **5a** (1.00 g, 2.350 mmol) was suspended in a mixture of chloroform (10 mL) and glacial acetic acid (10 mL). Aqueous hydrogen peroxide solution (35%, 1.5 mL) was added, and the mixture was stirred at r.t. for 2 d. Then, ice (50 g) was added, and the reaction solution was stirred for an additional 5 min, followed by extraction with chloroform (3 × 60 mL). The organic phase was washed with water (2 × 50 mL) and dried (sodium sulfate). After evaporation of the solvent, the obtained solid was purified by column chromatography (petroleum ether/ethyl acetate, 2:1) and finally dried in vacuo. It was a yellowish solid, with a yield of 0.921 g (89%) and m.p. of 90 °C. IR (ATR): ν_max_ = 2987, 1723, 1538, 1334, 1215, and 775 cm^−1^. ^1^H NMR (DMSO-*d_6_*, 400 MHz): δ 9.24 (d, *J* = 7.4 Hz, 1 H), 8.71 (d, *J* = 9.4 Hz, 1 H), 8.43 (d, *J* = 9.4 Hz, 1 H), 8.22 (d, *J* = 7.4 Hz, 1 H), 8.14 (s, 1 H, CHCl_2_), 8.00–7.92 (m, 2 H), 4.64 (d, *J* = 14.2 Hz, 1 H, SCH_2_), 4.41 (d, *J* = 14.2 Hz, 1 H, SCH_2_), 4.08 (q, *J* = 7.1 Hz, 2 H, OCH_2_), and 1.06 (t, *J* = 7.1 Hz, 3 H, CH_3_); ^13^C NMR (DMSO-*d_6_*, 100 MHz): δ 165.2 (C=O), 153.3 (Cq), 146.7 (Cq), 139.2 (Cq), 136.1 (Cq), 133.6 (Cq), 132.0 (CH), 131.1 (CH), 129.7 (Cq), 128.9 (CH), 128.5 (CH), 125.2 (CH), 122.9 (Cq), 121.5 (CH), 63.7 (CHCl_2_), 61.5 (OCH_2_), 57.9 (SCH_2_), and 13.7 (CH_3_); EIMS: *m/z* (%) 440 (12) [M]^+^, 323 (75) [M − NO_2_ − 2Cl]^+^, 304 (20) [M − SOCH_2_COOEt]^+^, 226 (75) [M − NO_2_ − Cl − SOCH_2_COOEt]^+^, and 191 (100) [M − NO_2_ − 2Cl − SOCH_2_COOEt]^+^. HRMS (ESI): *m/z* was calculated for C_18_H_15_Cl_2_N_2_O_5_S [M + H]^+^ 441.0073 and found 441.0076.

*Ethyl 2-((4-(dichloromethyl)-3-nitrobenzo[h]quinolin-2-yl)sulfonyl)acetate* (**9**). Acetate **5a** (0.200 g, 0.47 mmol) was suspended in a mixture of chloroform (4 mL) and glacial acetic acid (4 mL). Aqueous hydrogen peroxide solution (35%, 0.3 mL) was added, and the mixture was stirred at 70 °C for 6 h. Then, ice (50 g) was added, and the reaction mixture was stirred for an additional 5 min, followed by extraction with chloroform (6 × 60 mL). The organic phase was washed with water (2 × 50 mL), dried with sodium sulfate, and the solvent removed in vacuo. It was a yellowish solid, with a yield of 0.212 g (99%) and m.p. of 154 °C. IR (ATR): ν_max_ = 3043, 1745, 1553, 1344, 1263, and 749 cm^−1^. ^1^H NMR (DMSO-*d_6_*, 400 MHz): δ 9.25 (d, *J* = 8.0 Hz, 1 H), 8.71 (d, *J* = 9.2 Hz, 1 H), 8.52 (dd, *J* = 8.0, 1.6 Hz, 1 H), 8.25 (d, *J* = 8.0 Hz, 1 H), 8.13 (br s, 1 H, CHCl_2_), 8.01 (ddd, *J* = 9.2, 8.0, 1.6 Hz, 1 H), 7.96 (ddd, *J* = 9.2, 8.0, 1.6 Hz, 1 H), 5.41 (s, 2 H, SCH_2_), 3.95 (q, *J* = 7.1 Hz, 2 H, OCH_2_), and 0.87 (t, *J* = 7.1 Hz, 3 H, CH_3_); ^13^C NMR (DMSO-*d_6_*, 100 MHz): δ 162.5 (C=O), 145.7 (Cq), 144.9 (Cq), 137.4 (Cq), 135.9 (Cq), 133.7 (CH), 133.6 (Cq), 131.4 (CH), 129.4 (Cq), 129.3 (CH), 128.6 (CH), 125.5 (CH), 124.1 (Cq), 121.5 (CH), 63.5 (CHCl_2_), 61.7 (OCH_2_), 56.8 (SCH_2_), and 13.4 (CH_3_); EIMS: *m/z* (%) 456 (20) [M]^+^, 411 (10) [M − OEt]^+^, 363 (100) [M − OEt − NO_2_]^+^, 318 (45) [M − CH_2_COOEt − NO_2_]^+^, and 177 (70) [benzo[h]quinoline]^+^. HRMS (ESI): *m/z* was calculated for C_18_H_14_Cl_2_N_2_O_6_SNa [M + Na]^+^ 478.9842 and found 478.9847.

*4-(Dichloromethyl)-3-nitro-2-(phenylthio)benzo[h]quinoline* (**10a**). The product was synthesized according to benzo[*h*]quinoline **5a** from quinoline **8** (0.200 g, 0.45 mmol), diisopropyl-*N*-ethylamine (0.058 g, 0.076 mmol), and benzenethiol (0.050 g, 0.45 mmol). It was a yellowish solid, with a yield of 0.185 g (99%) and m.p. of 235 °C. IR (ATR): ν_max_ = 3062, 1729, 1538, 1324, 1247, and 714 cm^−1^. ^1^H NMR (CDCl_3_, 400 MHz): δ 8.59 (d, *J* = 9.6 Hz, 1 H), 8.28 (d, *J* = 8.0 Hz, 1 H), 7.89 (d, *J* = 9.6 Hz, 1 H), 7.85 (d, *J* = 8.0 Hz, 1 H), 7.71–7.68 (m, 3 H), 7.58–7.48 (m, 4 H), and 7.17 (s, 1 H, CHCl_2_); ^13^C-NMR (CDCl_3_, 100 MHz): δ 150.5 (Cq), 147.8 (Cq), 136.3 (2C, CH), 136.2 (Cq), 135.2 (Cq), 134.0 (Cq), 130.5 (Cq), 130.0 (CH), 129.9 (CH 129.5 (2C, CH), 128.9 (CH), 128.6 (Cq), 127.8 (CH), 127.7 (CH), 125.5 (CH), 122.1 (CH), 199.6 (Cq), and 63.5 (CHCl_2_); EIMS: *m/z* (%) 414 (20) [M]^+^, 304 (10) [M − SC_6_H_5_]^+^, 298 (15) [M − 2Cl − NO_2_]^+^, and 186 (25) [M − SC_6_H_5_ − 2Cl − NO_2_]^+^. HRMS (MS): *m/z* was calculated for C_20_H_12_Cl_2_N_2_O_2_S [M]^+^ 413.9991 and found 413.9996.

*4-(Dichloromethyl)-2-(naphthalen-2-ylthio)-3-nitrobenzo[h]quinoline* (**10b**). The product was prepared according to benzo[*h*]quinoline **5a** from quinoline **8** (0.200 g, 0.45 mmol), diisopropyl-*N*-ethylamine (0.058 g, 0. 076 mmol), and 2-naphthylthiol (0.072 g, 0.45 mmol). It was a yellowish solid, with a yield of 0.059 g (28%) and m.p. of 254 °C. IR (ATR): ν_max_ = 3053, 1538, 1323, 1220, 787, and 712 cm^−1^. ^1^H-NMR (CDCl_3_, 400 MHz): δ 8.59 (d, *J* = 9.2 Hz, 1 H), 8.25 (s, 1 H), 8.15 (d, *J* = 8.4 Hz, 1 H), 7.97 (d, *J* = 8.4 Hz, 1 H), 7.96 (d, *J* = 8.0 Hz, 1 H), 7.92 (d, *J* = 9.2 Hz, 1 H), 7.85 (d, *J* = 9.0 Hz, 1 H), 7.81 (d, *J* = 8.0 Hz, 1 H), 7.70 (dd, *J* = 9.0, 2.1 Hz, 1 H), 7.66–7.55 (m, 3 H), 7.29 (ddd, *J* = 8.0, 8.0, 2.0 Hz, 1 H), and 7.21 (s, 1 H, CHCl_2_); ^13^C-NMR (CDCl_3_, 100 MHz): δ 150.2 (Cq), 147.8 (Cq), 139.0 (Cq), 135.8 (CH), 135.2 (Cq), 133.9 (Cq), 133.8 (Cq), 133.6 (Cq), 132.3 (CH), 130.3 (Cq), 129.9 (CH), 128.9 (CH), 128.8 (CH), 128.0 (CH), 127.8 (CH), 127.7 (CH), 127.6 (CH), 127.4 (CH) 126.7 (CH), 125.9 (Cq), 125.5 (CH), 122.0 (CH), 119.7 (Cq), and 63.4 (CHCl_2_); EIMS: *m/z* (%) 464 (40) [M]^+^, 365 (35) [M − NO_2_ − C_4_H_4_]^+^, 349 (15) [M − NO_2_ − 2Cl]^+^, 336 (10) [M − naphthyl]^+^, 191 (40) [M − NO_2_ − 2Cl − naphthyl]^+^, and 127 (100) [naphthyl]^+^. HRMS (ESI): *m/z* was calculated for C_24_H_14_Cl_2_N_2_O_2_S [M]^+^ 464.0148 and found 464.0153.

*2-((4-Chlorophenyl)thio)-4-(dichloromethyl)-3-nitrobenzo[h]quinoline* (**10c**). The product was prepared according to benzo[*h*]quinoline **5a** from quinoline **8** (0.200 g, 0.45 mmol), diisopropyl-*N*-ethylamine (0.058 g, 0. 076 mmol), and 4-chlorobenzenethiol (0.065 g, 0.45 mmol). It was a yellowish solid, with a yield of 0.089 g (44%) and m.p. of 265 °C. IR (ATR): ν_max_ = 1520, 1475, 1326, 1088, 822, and 734 cm^−1^. ^1^H NMR (DMSO-*d_6_*, 400 MHz): δ 8.52 (d, *J* = 9.2 Hz, 1 H), 8.18 (d, *J* = 7.8 Hz, 1 H), 8.16 (d, *J* = 9.2 Hz, 1 H), 8.10 (s, 1 H, CHCl_2_), 8.05 (d, *J* = 7.8 Hz, 1 H), 7.79 (t, *J* = 7.8 Hz, 1 H), 7.76 (d, *J* = 8.6 Hz, 2 H), 7.67 (d, *J* = 8.6 Hz, 2 H), and 7.60 (t, *J* = 7.8 Hz, 1 H); ^13^C-NMR (DMSO-*d_6_*, 100 MHz): δ 149.2 (Cq), 146.6 (Cq), 139.2 (Cq), 137.5 (2C, CH), 135.3 (Cq), 135.2 (Cq), 133.6 (Cq), 130.5 (CH), 129.6 (2C, CH), 129.5 (CH), 128.1 (2C, CH), 127.1 (Cq), 124.3 (CH), 121.3 (CH), 119.4 (Cq), and 64.1 (CHCl_2_); EIMS: *m/z* (%) 447 (45) [M]^+^, 348 (45) [M − C_5_H_4_Cl]^+^, 304 (10) [M − SC_6_H_4_Cl]^+^, 235 (60) [M − SC_6_H_4_Cl − 2Cl]^+^, 191 (25) [M − SC_6_H_4_Cl − 2Cl − NO_2_]^+^, and 177 (100) [benzo[*h*]quinoline]^+^. HRMS (ESI): *m/z* was calculated for C_20_H_11_Cl_3_N_2_O_2_S [M]^+^ 447.9601 and found 447.9607.

*4-(Dichloromethyl)-2-((4-fluorophenyl)thio)-3-nitrobenzo[h]quinoline* (**10d**). To a solution of acetate **8** (0.400 g, 0.90 mmol) in dichloromethane (10 mL), diisopropyl-*N*-ethylamine (0.116 g, 0.90 mmol) was added under stirring. The solution was cooled to 0 °C and treated with 4-fluorobenzenethiol (0.115 g, 0.90 mmol). The reaction mixture was stirred for 3.5 h at r.t. Subsequently, the reaction mixture was acidified with aqueous HCl (18%, 1 mL), extracted with chloroform (3 × 30 mL), washed with water (1 × 50 mL), and dried with calcium chloride. After the evaporation of the solvent, the obtained solid was purified by column chromatography (petroleum ether/ethyl acetate, 10:1). The solvents were removed in vacuo to receive a yellowish solid, with a yield of 0.296 g (74%) and m.p. of 267 °C. IR (ATR): ν_max_ = 1522, 1488, 1328, 1220, 827, and 744 cm^−1^. ^1^H NMR (CDCl_3_, 600 MHz): δ 8.61 (d, *J* = 9.3 Hz, 1 H), 8.30 (d, *J* = 7.9 Hz, 1 H), 7.89 (d, *J* = 7.9 Hz, 1 H), 7.85 (d, *J* = 7.9 Hz, 1 H), 7.69 (t, *J* = 7.9 Hz, 1 H), 7.69–7.65 (m, 2 H), 7.55 (t, *J* = 7.9 Hz, 1 H), 7.26–7.22 (m, 2 H), and 7.17 (s, 1 H, CHCl_2_); ^13^C NMR (CDCl_3_, 150 MHz): δ 164.0 (CF, ^1^*J*_C,F_ = 250.9 Hz), 150.3 (Cq), 147.8 (Cq), 139.0 (Cq), 138.5 (CH, ^3^*J*_C,F_ = 8.3 Hz), 135.3 (Cq), 134.1 (Cq), 130.4 (Cq), 130.1 (CH), 129.0 (CH), 127.9 (CH), 127.8 (CH), 125.3 (CH), 123.9 (Cq, ^4^*J*_C,F_ = 3.4 Hz), 122.1 (CH), 119.8 (Cq), 116.7 (CH, ^2^*J*_C,F_ = 22.0 Hz), and 63.4 (CHCl_2_); EIMS: *m/z* (%) 432 (30) [M]^+^, 397 (8) [M − Cl]^+^, 333 (30) [M − Cl − F − NO_2_]^+^, and 285 (20) [M − CCl_2_ − F − NO_2_]^+^. HRMS (EI): *m/z* was calculated for C_20_H_11_Cl_2_FN_2_O_2_S [M]^+^ 431.9897 and found 431.9902.

*4-(Dichloromethyl)-3-nitro-2-(piperidin-1-yl)benzo[h]quinoline* (**11a**). To a solution of acetate **8** (0.127 g, 0.29 mmol) in dry toluene (10 mL), piperidine (0.025 g, 0.29 mmol) was added under stirring, followed by refluxing for an additional 6 h. Subsequently, the reaction mixture was cooled to r.t., acidified with aqueous HCl (18%, 1 mL), extracted with chloroform (3 × 30 mL), washed with water (1 × 50 mL), and dried with calcium chloride. After the evaporation of the solvent, the obtained solid was purified by column chromatography (petroleum ether/ethyl acetate, 10:1). The product was a red solid, with a yield of 0.091 g (80%) and m.p. of 236 °C. IR (ATR): ν_max_ = 2930, 2832, 1582, 1527, 1332, 1234, and 727 cm^−1^. ^1^H NMR (CDCl_3_, 400 MHz): δ 9.07 (d, *J* = 7.6 Hz, 1 H), 8.54 (d, *J* = 9.2 Hz, 1 H), 7.87 (d, *J* = 7.6 Hz, 1 H), 7.76 (d, *J* = 9.2 Hz, 1 H), 7.73–7.65 (m, 2 H), 7.08 (s, 1 H, CHCl_2_), 3.53–3.50 (m, 4 H), and 1.77–1.67 (m, 6 H); ^13^C NMR (CDCl_3_, 100 MHz): δ 149.5 (Cq), 146.9 (Cq), 135.9 (Cq), 134.3 (Cq), 134.0 (Cq), 130.5 (Cq), 129.4 (CH), 127.8 (CH), 127.0 (CH), 125.7 (CH), 125.4 (CH), 122.6 (CH), 116.0 (Cq), 63.8 (CHCl_2_), 49.9 (2C, NCH_2_), 25.7 (2C, CH_2_), and 24.4 (CH_2_); EIMS: *m/z* (%) = 389 (100) [M]^+^, 318 (60) [M − 2Cl]^+^, and 304 (40) [M − piperidine]^+^. HRMS (MS): *m/z* was calculated for C_19_H_17_Cl_2_N_3_O_2_ [M]^+^ 389.0692 and found 389.0699.

*4-(4-(Dichloromethyl)-3-nitrobenzo[h]quinolin-2-yl)morpholine* (**11b**). The product was prepared according to benzo[*h*]quinoline **11a** from quinoline **8** (0.200 g, 0.45 mmol) and morpholine (0.039 g, 0.45 mmol). The reaction mixture was stirred for 8 h at 70 °C. Then, purification by column chromatography (petroleum ether/ethyl acetate, 10:1) led to a red solid, with a yield of 0.095 g (54%) and m.p. of 233 °C. IR (ATR): ν_max_ = 2960, 2857, 1587, 1525, 1352, 1227, and 734 cm^−1^. ^1^H NMR (DMSO-*d_6_*, 400 MHz): δ 9.02 (d, *J* = 7.8 Hz, 1 H), 8.47 (d, *J* = 9.3 Hz, 1 H), 8.05 (d, *J* = 7.8 Hz, 1 H), 8.02 (d, *J* = 9.3 Hz, 1 H), 7.84–7.59 (m, 2 H), 7.83 (s, 1 H, CHCl_2_), 3.77 (t, *J* = 4.5 Hz, 4 H, OCH_2_), and 3.47 (t, *J* = 4.5 Hz, 4 H, NCH_2_); ^13^C-NMR (DMSO-*d_6_*, 100 MHz): δ 148.6 (Cq), 145.6 (Cq), 135.8 (Cq), 133.9 (Cq), 133.7 (Cq), 129.9 (CH), 129.4 (Cq), 128.0 (CH), 127.6 (CH), 126.5 (CH), 124.9 (CH), 121.7 (CH), 116.0 (Cq), 65.8 (2C, OCH_2_), 64.4 (CHCl_2_), and 48.7 (2C, NCH_2_); EIMS: *m/z* (%) 391 (100) [M]^+^, 357 (10) [M − Cl]^+^, 321 (20) [M − 2Cl]^+^, 305 (40) [M − morpholine]^+^, and 177 (65) [benzo[*h*]quinoline]^+^. HRMS (ESI): *m/z* was calculated for C_18_H_15_Cl_2_N_3_O_3_ [M]^+^ 391.0485 and found 391.0490.

*4-(Dichloromethyl)-2-((4-fluorophenyl)sulfinyl)-3-nitrobenzo[h]quinoline* (**12**). Quinoline **10d** (0.200 g, 0.45 mmol) was dissolved in a mixture of chloroform (5 mL) and glacial acetic acid (5 mL). Under ice cooling, aqueous hydrogen peroxide solution (35%, 0.3 mL) was added, followed by stirring for an additional 2 d at r.t. Subsequently, ice (50 g) was added, and the reaction mixture was stirred for 5 min and extracted with chloroform (5 × 60 mL). The organic layer was then washed with water (2 × 50 mL) and dried with sodium sulfate. After the evaporation of the solvent, the obtained solid was purified by column chromatography (petroleum ether/ethyl acetate, 2:1). It was a yellowish solid, with a yield of 0.196 g (95%) and m.p. of 187 °C. IR (ATR): ν_max_ = 1585, 1542, 1353, 1223, 1090, and 754 cm^−1^. ^1^H NMR (CDCl_3_, 400 MHz): δ 9.29 (d, *J* = 9.2 Hz, 1 H), 8.71 (d, *J* = 9.2 Hz, 1 H), 8.11 (d, *J* = 9.2 Hz, 1 H), 8.05 (d, *J* = 8.8 Hz, 1 H), 8.04 (d, *J* = 8.8 Hz, 1 H), 7.99–7.96 (m, 1 H), 7.87–7.85 (m, 2 H), 7.21 (s, 1 H, CHCl_2_), and 7.26–7.20 (m, 2 H); ^13^C NMR (CDCl_3_, 100 MHz): δ 164.9 (CF, ^1^*J*_C,F_ = 253.3 Hz), 153.7 (Cq), 148.2 (Cq), 138.2 (Cq, ^3^*J*_C,F_ = 8.2 Hz), 138.1 (Cq), 136.0 (Cq), 134.0 (Cq), 131.9 (CH), 131.0 (CH), 130.6 (Cq), 128.8 (CH), 128.3 (CH), 128.2 (2 C, CH), 125.8 (CH), 123.3 (Cq, ^4^*J*_C,F_ = 3.0 Hz), 122.0 (CH), 116.8 (CH, ^2^*J*_C,F_ = 22.6 Hz), and 62.5 (CHCl_2_); EIMS: *m/z* (%) 446 (3) [M]^+^, 403 (10) [M − NO_2_]^+^, 368 (20) [M − NO_2_ − Cl]^+^, 333 (8) [M − NO_2_ − Cl]^+^, and 143 (100) [SOPhF]^+^. HRMS (ESI): *m/z* was calculated for C_20_H_11_Cl_2_FN_2_O_3_SNa [M + Na]^+^ 470.9744 and found 470.9749.

*2-((4-(Dichloromethyl)-3-nitrobenzo[h]quinolin-2-yl)thio)acetic acid (***13**). Acetate **5a** (1.500 g, 3.527 mmol) was suspended in conc. sulfuric acid (25 mL) and stirred at 90 °C for 5 h. Subsequently, ice water (100 mL) was added under stirring. The reaction mixture was extracted with chloroform (4 × 50 mL) and washed with water (3 × 70 mL). The organic phase was dried with sodium sulfate, and the solvent removed in vacuo. The product was a yellowish solid, with a yield of 1.379 g (99%) and m.p. of 204 °C. IR (ATR): ν_max_ = 2119, 1705, 1524, 1359, 1221, 968, and 751 cm^−1^. ^1^H NMR (DMSO-*d_6_*, 600 MHz): δ 13.05 (br s, 1 H, OH), 9.13 (d, *J* = 8.0 Hz, 1 H), 8.52 (d, *J* = 9.6 Hz, 1 H), 8.17 (d, *J* = 8.0 Hz, 1 H), 8.09 (d, *J* = 8.0 Hz, 1 H), 8.07 (s, 1 H, CHCl_2_), 7.87 (d, *J* = 8.0 Hz, 1 H), 7.80 (d, *J* = 8.0 Hz, 1 H), and 4.29 (s, 2 H, SCH_2_); ^13^C NMR (DMSO-*d_6_*, 150 MHz): δ 170.1 (C=O), 149.6 (Cq), 147.2 (Cq), 139.8 (Cq), 135.3 (Cq) 134.2 (Cq), 130.9 (CH), 129.9 (Cq), 129.6 (CH), 128.7 (CH), 128.6 (CH), 125.8 (CH), 121.9 (CH), 119.2 (Cq), 64.6 (CHCl_2_), and 34.0 (SCH_2_); EIMS: *m/z* (%) 396 (20) [M]^+^, 303 (25) [M − SCH_2_COOH]^+^, 252 (100) [M − SCH_2_COOH − C_4_H_4_]^+^, and 177 (30) [benzo[*h*]quinoline]^+^. HRMS (ESI): *m/z* was calculated for C_16_H_11_Cl_2_N_2_O_4_S [M + H]^+^ 396.9811 and found 396.9813.

*4-(Dichloromethyl)-3-nitrobenzo[h]quinolin-2(1H)-one* (**14**). To a suspension of acid **13** (0.840 g, 2.120 mmol) in a mixture of chloroform (15 mL) and glacial acetic acid (15 mL), aqueous hydrogen peroxide solution (35%, 1.2 mL) was added and stirred at r.t. for 2 d. Subsequently, water (50 mL) was added, and the reaction mixture was stirred for an additional 30 min. After extraction with chloroform (4 × 50 mL), the organic phase was dried with sodium sulfate, and the solvent removed in vacuo. The product was a yellowish solid, with a yield of 0.475 g (70%) and m.p. of 286 °C. IR (ATR): ν_max_ = 2923, 1654, 1537, 1350, 1216, and 736 cm^−1^. ^1^H NMR (DMSO-*d_6_*, 400 MHz): δ 13.3 (bs, 1H, NH), 8.90 (d, *J* = 8.0 Hz, 1 H), 8.33 (d, *J* = 9.2 Hz, 1 H), 8.01 (d, *J* = 8.0 Hz, 1 H), 7.88 (s, 1 H, CHCl_2_), 7.86 (d, *J* = 8.0 Hz, 1 H), 7.76 (t, *J* = 8.0 Hz, 1 H), and 7.69 (t, *J* = 8.0 Hz, 1 H); ^13^C-NMR (DMSO-*d_6_*, 100 MHz): δ 153.9 (Cq), 136.5 (Cq), 134.0 (Cq), 131.3 (Cq), 130.0 (Cq), 129.7 (CH), 128.4 (CH), 127.4 (CH), 123.9 (Cq), 123.8 (CH), 123.2 (CH), 121.9 (CH), 119.0 (Cq), and 64.0 (CHCl_2_); EIMS: *m/z* (%) 322 (100) [M]^+^, 252 (25) [M − 2Cl]^+^, 229 (75) [M − Cl − NO_2_ − O]^+^, 207 (20) [M − 2Cl − NO_2_]^+^, and 177 (60) [benzo[*h*]quinoline]^+^. HRMS (ESI): *m/z* was calculated for C_14_H_8_Cl_2_N_2_O_3_Na [M + Na]^+^ 344.9804 and found 344.9816.

*4-(Dichloromethyl)-3-nitrobenzo[h]quinolin-2-yl 2,4-dichlorobenzoate* (**15a**). To a solution of quinolin-2(1*H*)-one **14** (0.086 g, 0.269 mmol) and 2,4-dichlorobenzoyl chloride (0.056 g, 0.269 mmol) in anhydrous THF (5 mL) under a nitrogen atmosphere, triethylamine (0.029 g, 0.290 mmol) was added at r.t. and stirred for 1 d. Subsequently, the reaction mixture was diluted with ice water (30 mL) at stirring, extracted with chloroform (3 × 30 mL), washed with water (1 × 50 mL), and saturated aqueous NaHCO_3_ solution (10 mL) and water (30 mL). The organic phase was dried with sodium sulfate, the solvent removed in vacuo, and the residue purified by column chromatography (petroleum ether/ethyl acetate, 3:1). The product was a yellowish solid, with a yield of 0.100 g (77%) and m.p. of 177 °C. IR (ATR): ν_max_ = 2956, 1757, 1535, 1335, 1214, 1150, and 732 cm^−1^. ^1^H NMR (CDCl_3_, 400 MHz): δ 9.09 (d, *J* = 7.8 Hz, 1 H), 8.64 (d, *J* = 9.4 Hz, 1 H), 8.05 (d, *J* = 8.5 Hz, 1 H), 8.02 (d, *J* = 9.4 Hz, 1 H), 7.92 (d, *J* = 7.8 Hz, 1 H), 7.76 (t, *J* = 7.8 Hz, 1 H), 7.70 (t, *J* = 7.8 Hz, 1 H), 7.54 (s, 1 H), 7.36 (d, *J* = 8.5 Hz, 1 H), and 7.13 (s, 1 H, CHCl_2_); ^13^C NMR (CDCl_3_, 100 MHz): δ 161.0 (C=O), 147.1 (Cq), 145.4 (Cq), 140.5 (Cq), 137.7 (Cq), 136.8 (Cq), 134.1 (Cq), 133.7 (CH), 131.8 (CH), 130.6 (Cq), 130.6 (CH), 130.5 (Cq), 130.2 (CH), 128.2 (CH), 128.0 (CH), 127.5 (CH), 126.0 (CH), 125.3 (Cq), 121.9 (CH), 121.8 (Cq), and 62.8 (CHCl_2_); EIMS: *m/z* (%) 493 (3) [M]^+^, and 172 (100) [COC_6_H_3_Cl_2_]^+^. HRMS (ESI): C_21_H_10_Cl_4_N_2_O_4_: *m/z* was calculated for [M]^+^ 493.9389 and found 493.9397.

*4-(Dichloromethyl)-3-nitrobenzo[h]quinolin-2-yl 4-bromobenzoate* (**15b**). The product was prepared according to benzo[*h*]quinoline **15a** from quinoline **14** (0.100 g, 0.31 mmol), 4-bromobenzoyl chloride (0.068 g, 0.31 mmol), and triethylamine (0.034 g, 0.34 mmol). The reaction mixture was stirred for 4 h. The product was a yellow solid, with a yield of 0.122 g (78%) and m.p. of 239 °C. IR (ATR): ν_max_ = 2924, 1754, 1558, 1508, 1398, 1227, 1056, and 752 cm^−1^. ^1^H NMR (CDCl_3_, 400 MHz): δ 9.15 (d, *J* = 8.0 Hz, 1 H), 8.72 (d, *J* = 9.3 Hz, 1 H), 8.09 (d, *J* = 9.3 Hz, 1 H), 8.07 (d, *J* = 8.5 Hz, 2 H), 7.99 (d, *J* = 8.0 Hz, 1 H), 7.82 (ddd, *J* = 8.0, 8.0, 1.5 Hz, 1 H), 7.76 (ddd, *J* = 8.0, 8.0, 1.6 Hz, 1 H), 7.71 (d, *J* = 8.5 Hz, 2 H), and 7.20 (s, 1 H, CHCl_2_); ^13^C NMR (CDCl_3_, 400 MHz): δ 163.0 (C=O), 147.1 (Cq), 145.7 (Cq), 137.6 (Cq), 134.1 (Cq), 132.4 (Cq), 132.3 (2C, CH), 132.2 (2C, CH), 130.6 (CH), 130.5 (Cq), 130.2 (Cq), 130.1 (CH), 128.2 (CH), 128.0 (CH), 126.7 (Cq), 126.0 (CH), 121.9 (CH), 121.7 (Cq), and 62.8 (CHCl_2_); EIMS: *m/z* (%) 505 (75) [M]^+^, and 392 (100) [M − Cl − Br]^+^. HRMS (MS): *m/z* was calculated for C_21_H_11_Cl_2_N_2_O_4_Br [M]^+^ 503.9274 and found 503.9280.

*1,1’-(3,4,4-Trichloro-2-nitrobuta-1,3-diene-1,1-diyl)bis(1H-benzo[d][1,2,3]triazole)* (**16a**). The product was prepared according to the published literature [20] from nitrodiene **1** and 1*H*-benzotriazole. The yield was 76%. All spectral data are in accordance with the literature.

*1,1’-(3,4,4-Trichloro-2-nitrobuta-1,3-diene-1,1-diyl)bis(1H-1,2,4-triazole)* (**16b**). The product was synthesized according to a previously published procedure [21] from nitrodiene **1** and 1*H*-1,2,4-triazole. The yield was 92%. All spectral data are in accordance with the literature.

*1,1’-(3,4,4-Trichloro-2-nitrobuta-1,3-diene-1,1-diyl)bis(1H-pyrazole)* (**16c**). To a solution of 2.72 g (40.00 mmol) of 1*H*-pyrazole in diethyl ether (50 mL) at 0 °C, a solution of nitrodiene **1** (2.71 g, 10.00 mmol) in ether (5 mL) was added. The resulting mixture was stirred at 0 °C for 1 h and at r.t. for an additional 20 h. The solvent was removed, and then cold water (50 mL) was added while stirring. The resulting precipitate was filtered off with suction, washed with water (2 × 20 mL) and cold methanol (5 mL), and dried in vacuo to give **16c**. The product was a yellow solid, with a yield of 3.01 g (90%) and m.p. of 133–134 °C. IR (KBr): ν_max_ = 3102, 1653, 1532 (NO_2_), 1394, 1314 (NO_2_), 957, and 772 cm^−1^. ^1^H NMR (CDCl_3_, 400 MHz): δ 7.93 (d, *J* = 1.3 Hz, 1 H), 7.87 (d, *J* = 1.3 Hz, 1 H), 7.56 (d, *J* = 3.0 Hz, 1 H), 7.51 (d, *J* = 2.8 Hz, 1 H), and 6.63–6.58 (m, 2 H); ^13^C NMR (CDCl_3_, 100 MHz): δ 146.0 (CH), 145.4 (CH), 138.3 (C1), 132.1 (CH), 131.8 (CH), 129.7 (C-NO_2_), 129.0 and 121.1 (C_2_Cl_3_), and 111.0 (2 CH); EIMS: *m/z* (%) 333 (2) [M]^+^, 298 (100) [M − Cl]^+^, 263 (10) [M − 2Cl], 252 (37) [M − Cl − NO_2_]^+^, and 217 (55) [M − 2Cl − NO_2_]^+^. HRMS (ESI): *m/z* calculated for C_10_H_6_Cl_3_N_5_O_2_Na [M + Na]^+^ 355.9479 and found 355.9485.

*(E)-N-(1-(1H-Benzo[d][1,2,3]triazol-1-yl)-3,4,4-trichloro-2-nitrobuta-1,3-dien-1-yl)naphthalen-1-amine* (**17a**). The product was obtained according to a previously published procedure [22] from bisazole **16a** and naphthalen-1-amine. The yield was 90%. All spectral data are in accordance with the literature.

*(E)-N-(3,4,4-Trichloro-2-nitro-1-(1H-1,2,4-triazol-1-yl)buta-1,3-dien-1-yl)naphthalen-1-amine* (**17b**). Naphthalen-1-amine (1.50 g, 10.5 mmol) was added to a suspension of bisazole **16b** (3.37 g, 10.0 mmol) in MeOH (40 mL) at 0 °C within 2 min. The resulting mixture was stirred at 0 °C for 1 h and was then kept at r.t. overnight. Subsequently, the supernatant liquid was concentrated to a volume of about 15 mL, cooled to 10 °C, and then treated with aqueous HCl (5%, 80 mL). The mixture was stirred for an additional 20 min. The formed precipitate was collected on a suction filter, washed with water (2 × 20 mL) and cold MeOH (1 × 10 mL), and then finally dried under reduced pressure. The product was a yellow solid, with a yield of 3.53 g (86%) and m.p. of 114–115 °C. IR (KBr): ν_max_ = 3129, 1620, 1575 (NO_2_), 1334 (NO_2_), 1262, 1179, and 773 cm^−1^. ^1^H NMR (CDCl_3_, 400 MHz): δ 11.73 (br s, 1 H, NH), 8.04 (d, *J* = 8.3 Hz, 1 H), 8.00 (s, 1 H, CHN), 7.89 (d, *J* = 7.3 Hz, 1 H), 7.87 (s, 1 H, CHN), 7.78 (d, *J* = 8.3 Hz, 1 H), 7.72–7.55 (m, 2 H), 7.26 (t, *J* = 7.7 Hz, 1 H), and 6.91 (d, *J* = 7.3 Hz, 1H); ^13^C NMR (CDCl_3_, 100 MHz): δ 153.0 (CHN), 147.8 (C1), 144.6 (CHN), 134.0 (NH-Cq), 130.6 (Cq), 129.2 (CH), 128.9 (Cq), 128.8 (CH), 128.5 (Cq), 128.1 (CH), 127.3 (CH), 125.1 (CH), 122.5 (CH), 121.0 (CH), 120.6 (Cq), and 118.8 (C-NO_2_); EIMS: *m/z* (%) 409 (35) [M]^+^, 374 (1) [M − Cl]^+^, 327 (6) [M − Cl − HNO_2_]^+^, 214 (24), 143 (74), and 133 (100). HRMS (ESI): *m/z* was calculated for C_16_H_11_Cl_3_N_5_O_2_ [M + H]^+^ 409.9973 and found 409.9978.

*(E)-N-(3,4,4-Trichloro-2-nitro-1-(1H-pyrazol-1-yl)buta-1,3-dien-1-yl)naphthalen-1-amine* (**17c**). To a suspension of bisazole **16c** (3.35 g, 10.0 mmol) in diethyl ether (40 mL), naphthalen-1-amine (1.50 g, 10.5 mmol) was added at −30 °C within 5 min. The resulting mixture was stirred at −30 °C for 4 h and then kept at r.t. overnight. The mixture was concentrated to a volume of about 10 mL by means of a rotary evaporator. Subsequently, after the addition of cold water (200 mL), aqueous HCl (37%, 5 mL) was added dropwise. After 20 min stirring, the mixture was extracted with chloroform (3 × 70 mL). The combined organic layers were washed with brine (150 mL) and dried with calcium chloride. After the evaporation of the solvent, the crude product was purified by means of column chromatography (petroleum ether/ethyl acetate, 3:1). The evaporation of all solvents gave nitrodiene **17c** as a light brown solid. The yield was 3.69 g (90%), with m.p. of 177–179 °C. IR (KBr): ν_max_ = 3222, 1617, 1571 (NO_2_), 1485, 1360 (NO_2_), 1084, and 757 cm^−1^. ^1^H NMR (CDCl_3_, 400 MHz): δ 11.91 (br s, 1 H, NH), 8.11 (d, *J* = 8.2 Hz, 1 H), 7.89 (d, *J* = 8.1 Hz, 1 H), 7.75 (d, *J* = 8.3 Hz, 1 H), 7.67 (ddd, *J* = 8.2, 7.2, 1.1 Hz, 1 H), 7.59 (ddd, *J* = 8.2, 7.0, 1.1 Hz, 1 H), 7.58 (d, *J* = 2.0 Hz 1 H, CH-N), 7.37 (d, *J* = 2.4 Hz, 1 H, CHN), 7.24 (t, *J* = 7.8 Hz, 1 H), 6.78 (d, *J* = 7.3 Hz, 1 H), and 6.23 (dd, *J* = 2.4, 2.0 Hz, 1 H); ^13^C NMR (CDCl_3_, 100 MHz): δ 150.6 (NCN), 143.3 (CHN), 134.0 (NH-Cq), 131.6 (Cq), 131.4 (CH), 128.7 (CH), 128.3 (CH), 128.2 (Cq), 127.9 (CH), 127.7 (Cq), 127.1 (CH), 125.2 (CH), 122.1 (Cq), 121.44 (CH), 121.2 (CH), 118.1 (C-NO_2_), and 109.1 (CH); EIMS: *m/z* (%) 408 (2) [M]^+^, 372 (4) [M − HCl]^+^, 362 (5) [M − NO_2_]^+^, 327 (3) [M − NO_2_ - HCl]^+^, (7), 292 (4) [M − 2Cl − NO_2_]^+^, and 169 (100). HRMS (ESI): *m/z* was calculated for C_17_H_11_Cl_3_N_4_O_2_Na [M + Na]^+^ 430.9840 and found 430.9845.

*2-(1H-Benzo[d][1,2,3]triazol-1-yl)-4-(dichloromethyl)-3-nitrobenzo[h]quinoline* (**18a**). Triethylamine (202 mg, 2.00 mmol) was added to a solution of nitrodiene **17a** (461 mg, 1.00 mmol) in anhydrous chloroform (20 mL) at 0 °C within 2 min. The resulting mixture was stirred at 0 °C for 1 h and then kept at r.t. overnight. Subsequently, the supernatant liquid was evaporated in vacuo, and MeOH (10 mL) was added to the residue. The mixture was stirred for 10 min; the formed precipitate was then filtered off with suction; washed successively with aqueous HCl (5%, 1 × 10 mL), water (2 × 10 mL), and MeOH (1 × 5 mL), and then dried under reduced pressure. The product was a yellowish solid, with a yield of 369 mg (87%) and m.p. of 260–262 °C. IR (KBr): ν_max_ = 3007, 1578, 1542 (NO_2_), 1459, 1359 (NO_2_), 1205, and 741 cm^−1^. ^1^H NMR (DMSO-*d_6_*, 600 MHz): δ 9.08 (d, *J* = 7.9 Hz, 1 H), 8.72 (d, *J* = 9.3 Hz, 1 H), 8.53 (d, *J* = 8.3 Hz, 1 H), 8.39 (d, *J* = 9.4 Hz, 1 H), 8.33 (ddd, *J* = 8.3, 0.8, 0.8 Hz, 1 H), 8.22 (dd, *J* = 7.3, 1.2 Hz, 1 H), 8.17 (br s, 1 H, CHCl_2_), 7.96 (ddd, *J* = 7.3, 7.3, 1.4 Hz, 1 H), 7.95–7.91 (m, 2H), and 7.69 (ddd, *J* = 8.3, 7.1, 1.0 Hz, 1 H); ^13^C NMR (DMSO-*d_6_*, 150 MHz): δ 146.0 (Cq), 145.5 (Cq), 137.7 (Cq), 137.5 (Cq), 134.6 (C-NO_2_), 134.0 (Cq), 131.8 (Cq), 131.2 (CH), 131.1 (CH), 130.7 (CH), 129.9 (Cq), 129.1 (CH), 128.7 (CH), 126.4 (CH), 125.1 (CH), 121.6 (CH), 121.5 (Cq), 120.3 (CH), 113.4 (CH), and 64.1 (CHCl_2_); EIMS: *m/z* (%) 423 (12) [M]^+^, 395 (4) [M − N_2_] ^+^, 349 (7) [M − N_2_ − NO_2_] ^+^, 302 (20), 279 (10), 266 (10), 240 (12), and 92 (100). HRMS (ESI): *m/z* was calculated for C_20_H_11_Cl_2_N_5_O_2_Na [M + Na]^+^ 446.0182 and found 446.0187.

*4-(Dichloromethyl)-3-nitro-2-(1H-1,2,4-triazol-1-yl)benzo[h]quinoline* (**18b**). The product was prepared according to quinoline **18a** from nitrodiene **17b** (411 mg, 1.00 mmol) and triethylamine (202 mg, 2.00 mmol). The product was a beige solid, with a yield of 135 mg (36%) and m.p. of 247–249 °C. IR (KBr): ν_max_ = 3008, 1547 (NO_2_), 1501, 1364 (NO_2_), 1198, 1009, and 732 cm^−1^. ^1^H NMR (DMSO-*d_6_*, 600 MHz): δ 9.89 (s, 1 H, ^1^*J*_C,H_ = 222 Hz, CHN), 9.27 (d, *J* = 8.1 Hz, 1 H), 8.63 (d, *J* = 9.3 Hz, 1 H), 8.44 (s, 1 H, ^1^*J*_C,H_ = 209 Hz, CHN), 8.32 (d, *J* = 9.3 Hz, 1 H), 8.15 (d, *J* = 8.3 Hz, 1 H), 8.05 (br s, 1 H, CHCl_2_), 7.92 (ddd, *J* = 7.8, 7.1, 1.0 Hz, 1 H), and 7.87 (ddd, *J* = 8.1, 7.1, 1.1 Hz, 1 H); ^13^C NMR (DMSO-*d_6_*, 150 MHz): δ 153.9 (CHN), 145.6 (Cq), 145.5 (CHN), 137.2 (Cq), 136.5 (Cq), 133.9 (Cq), 132.9 (C-NO_2_), 131.04 (CH), 130.98 (CH), 129.6 (Cq), 128.7 (CH), 128.5 (CH), 125.8 (CH), 121.6 (Cq), 121.5 (CH), and 64.0 (CHCl_2_); EIMS: *m/z* (%) 373 (100) [M]^+^, 338 (2) [M − Cl]^+^, 327 (7) [M − NO_2_]^+^, 303 (20), 292 (10) [M − NO_2_ − Cl]^+^, 280 (45), 269 (15), and 253 (25). HRMS (ESI): *m/z* was calculated for C_16_H_10_Cl_2_N_5_O_2_ [M + H]^+^ 374.0206 and found 374.0211.

*4-(Dichloromethyl)-3-nitro-2-(1H-pyrazol-1-yl)benzo[h]quinoline* (**18c**). The product was prepared according to quinoline **18a** from nitrodiene **17c** (410 mg, 1.00 mmol) and triethylamine (202 mg, 2.00 mmol). It was a light brown solid, with a yield of 291mg (78%) and m.p. of 197–199 °C. IR (KBr): ν_max_ = 3006, 1545 (NO_2_), 1502, 1395, 1359 (NO_2_), 1260, and 739 cm^−1^. ^1^H NMR (CDCl_3_, 400 MHz): δ 9.14–9.09 (m, 1 H), 8.77 (dd, *J* = 2.7, 0.6 Hz, 1 H, ^1^*J*_C,H_ = 194 Hz, CH-N), 8.69 (d, *J* = 9.3 Hz, 1 H), 7.99 (d, *J* = 9.3 Hz, 1 H), 7.96–7.92 (m, 1 H), 7.81 (dd, *J* = 1.6, 0.6 Hz, 1 H, CH=N), 7.79 (ddd, *J* = 7.2, 7.1, 1.7 Hz, 1 H), 7.79 (ddd, *J* = 7.3, 7.3, 1.6 Hz, 1 H), 7.11 (s, ^1^*J*_C,H_ = 179 Hz, 1 H, CHCl_2_), and 6.60 (dd, *J* = 2.7, 1.6 Hz, ^1^*J*_C,H_ = 179 Hz, 1 H); ^13^C NMR (CDCl_3_, 100 MHz): δ 0.1 (Cq), 143.7 (CH=N), 138.3 (Cq), 136.8 (Cq), 134.0 (Cq), 132.8 (C-NO_2_), 130.3 (Cq), 130.2 (CH), 129.3 (CH), 129.0 (CH), 128.1 (CH), 128.0 (CH), 125.2 (CH), 122.2 (CH), 120.7 (Cq), 108.8 (CH), and 63.2 (CHCl_2_); EIMS: *m/z* (%) 372 (100) [M]^+^, 355 (2) [M − OH]^+^, 326 (10) [M − NO_2_]^+^, 291 (24) [M − NO_2_ − Cl]^+^, 279 (23), 256 (25), and 243 (12) [M − NO_2_ − CHCl_2_]^+^. HRMS (ESI): *m/z* was calculated for C_17_H_10_Cl_2_N_4_O_2_Na [M + Na]^+^ 395.0073 and found 395.0079.

#### 3.1.1. Crystal Data

X-ray single-crystal structure analysis for ethyl (*E*)-2-((1,3,4,4-tetrachloro-2-nitrobuta-1,3-dien-1-yl)thio)acetate C_8_H_7_Cl_4_NO_4_S (**3a**)**,** M = 355.01 g mol^−1^: A suitable single crystal of the title compound was selected under a polarization microscope and mounted in a glass capillary (d = 0.3 mm). The crystal structure was determined by X-ray diffraction analysis using graphite monochromated Mo-K_α_ radiation (0.71073 Å) [T = 223(2) K], whereas the scattering intensities were collected using a single-crystal diffractometer STOE IPDS II (Stoe & Cie GmbH, Darmstadt, Germany). The crystal structure was solved by the Direct Methods using SHELXS and refined using alternating cycles of least-squares refinements against F^2^ (SHELXL). All non-H atoms were located in the Difference Fourier maps and were refined with anisotropic displacement parameters. The H positions were determined by a final Difference Fourier Synthesis [23].

C_8_H_7_Cl_4_NO_4_S (**3a**) crystallized in the triclinic space group P1 (no. 2), with lattice parameters a = 7.685(1) Å, b = 8.189(2) Å, c = 12.236(2) Å, α = 77.66(1)°, β = 76.20(1)°, γ = 68.33(1)°, V = 688.2(2) Å^3^, Z = 2, d_calc._ = 1.713 g cm^−3^, and F(000) = 356 as determined using 2558 independent reflections and 191 parameters. R1 = 0.0568 [I > 2σ(I)], wR2 = 0.1527 [I > 2σ(I)], goodness of fit on F^2^ = 1.043, and residual electron density 1.145 and −1.098 e Å^−3^. Further details of the crystal structure investigations have been deposited with the Cambridge Crystallographic Data Center, CCDC 1583680. The following supporting information can be found in Supplementary Materials, checkCIF **3a**.

X-ray single-crystal structure analysis for ethyl 2-((4-(dichloromethyl)-3-nitrobenzo[*h*]quinolin-2-yl)thio)acetate C_18_H_14_Cl_2_N_2_O_4_S (**5a**), M = 425.27 g mol^−1^: A suitable single crystal of the title compound was selected under a polarization microscope and mounted in a glass capillary (d = 0.3 mm). The crystal structure was determined by X-ray diffraction analysis using graphite monochromated Mo-K_α_ radiation (0.71073 Å) [T = 223(2) K], whereas the scattering intensities were collected using a single-crystal diffractometer (STOE IPDS II). The crystal structure was solved by the Direct Methods using SHELXS and refined using alternating cycles of least-squares refinements against F^2^ (SHELXL). All non-H atoms were located in the Difference Fourier maps and were refined with anisotropic displacement parameters. The H positions were determined by a final Difference Fourier Synthesis [23].

C_18_H_14_Cl_2_N_2_O_4_S (**5a**) crystallized in the monoclinc space group P2_1_/n (no. 14), with lattice parameters a = 11.504(2) Å, b = 11.520(2) Å, c = 14.139(2) Å, β = 101.17(1)°, V = 1838.3(5) Å^3^, Z = 4, d_calc._ = 1.537 g cm^−3^, and F(000) = 872 as determined using 3257 independent reflections and 295 parameters. R1 = 0.0642 [I > 2σ(I)], wR2 = 0.1298 [I > 2σ(I)], goodness of fit on F^2^ = 1.032, and residual electron density 1.123 and −1.042 e Å^−3^. Further details of the crystal structure investigations have been deposited with the Cambridge Crystallographic Data Center, CCDC 1583675. The following supporting information can be found in Supplementary Materials, checkCIF **5a**.

X-ray single-crystal structure analysis for 4-(4-(dichloromethyl)-3-nitrobenzo[*h*]quinolin-2-yl)morpholine C_18_H_15_Cl_2_N_3_O_3_ (**11b**), M = 392.23 g mol^−1^: A suitable single crystal of the title compound was selected under a polarization microscope and mounted in a glass capillary (d = 0.3 mm). The crystal structure was determined by X-ray diffraction analysis using graphite monochromated Mo-K_α_ radiation (0.71073 Å) [T = 223(2) K], whereas the scattering intensities were collected using a single-crystal diffractometer (STOE IPDS II). The crystal structure was solved by the Direct Methods using SHELXS and refined using alternating cycles of least-squares refinements against F^2^ (SHELXL). All non-H atoms were located in the Difference Fourier maps and were refined with anisotropic displacement parameters. The H positions were determined by a final Difference Fourier Synthesis [23].

C_18_H_15_Cl_2_N_3_O_3_ (**11b**) crystallized in the monoclinc space group P2_1_/c (no. 14), with lattice parameters a = 7.1031(9) Å, b = 22.053(2) Å, c = 11.362(1) Å, β = 99.70(1)°, V = 1754.3(4) Å^3^, Z = 4, d_calc._ = 1.485 g cm^−3^, and F(000) = 808 as determined using 3328 independent reflections and 295 parameters. R1 = 0.0528 [I > 2σ(I)], wR2 = 0.1378 [I > 2σ(I)], goodness of fit on F^2^ = 1.075, and residual electron density 0.335 and −0.577 e Å^−3^. Further details of the crystal structure investigations have been deposited with the Cambridge Crystallographic Data Center, CCDC 1583679. The following supporting information can be found in Supplementary Materials, checkCIF **11b**.

#### 3.1.2. Antibacterial Assays

Overnight cultures of the bacteria were grown aerobically at 37 °C in Müller–Hinton broth with added 1% glucose and a pH of 7.2 for Gram-negative strains, or with a Trypticase soy yeast extract medium (TSY—30 g/L Trypticase soy broth, 3 g/L yeast extract, pH 7.2) for Gram-positive strains. The cultures were adjusted to an OD600 nm of 0.001, which resulted in a final start OD600 nm of 0.0005 in the test. A total of 25 μL of test culture was added to 25 μL of a serial dilution of the test compounds in the appropriate medium for the different strains in accordance with standardized procedures in 384-well plates. For screening purposes, the residual absorbance in % was tested at the compound concentrations of 5 and 50 µM. For the selected compounds, the concentration-dependent growth inhibition curves were recorded from the stock solutions in DMSO at the final concentrations of 100, 50, 25, 12.5, 6,25, 3.125, 1.56, 0.78, 0.39, and 0.2 µM. As the positive control compounds, Linezolid (both MRSA strains) Ciprofloxacin (*E. faecium, E. coli*), and Amikacin (*P. aeruginosa*) were applied. The highest DMSO concentration in the assay was 1%, which had no apparent effect on the growth of the bacteria. After an incubation time of 18 h at 37 °C under moist conditions, the optical density at 600 nm was measured with a Fusion Universal Microplate Analyzer (Perkin–Elmer, Waltham, MA, USA). The lowest concentration that completely suppressed growth defined the MIC values. The following bacterial strains were used: Gram-negative: *Escherichia coli* (DSM 1116) and *Pseudomonas aeruginosa* PA7 (DSM 24068), and Gram-positive: *Staphylococcus aureus* MRSA (clinical isolate, RKI 11-02670) and *Staphylococcus aureus* MRSA (DSM 11822). The MIC values were determined by curve fitting with Sigma Plot.

#### 3.1.3. Antiproliferative Assays

The effects of the compounds on cell viability were probed with a WST-1 test using the procedure of Ishiyama et al. [24] as modified by Sasse et al. [25]. The following cell lines were used: mouse fibroblast cell line L929 (DSM ACC 2), human cervical carcinoma cell line KB-3-1 (DSM ACC 158), and human breast cancer cell line MCF-7 (DSM ACC 115). In addition, the conditional immortalized human fibroblast cell line FS4-LTM (InScreenex, Braunschweig, Germany) was used without doxycyclin to induce primary cell-like behavior (Pub. No.: US2011/0189142 A2). Briefly, the subconfluent cells were washed with Earle’s Balanced Salt Solution (Gibco) without Ca and Mg ions, trypsinized, and re-suspended in Dulbecco’s modified eagle’s medium that contained 5% fetal bovine serum (FBS; L929, KB-3-1, FS4-LTM) or Roswell Park Memorial Institute medium that contained 5% FBS, 0.5% Minimum Essential Medium Non-Essential Amino Acids, Gibco (MEM NEAA), 0.5% GlutaMAX (Gibco), and insulin at 5 μg/mL (MCF-7). A total of 25 µL of serial dilutions of the test compounds (100–0.2 µM), which were made with a pipetting robot (epMotion, Eppendorf, Hamburg, Germany), was added to 25 µL aliquots of a cell suspension (1500 cells for KB3-1 and L929, 3000 cells for MCF-7, and 7500 cells for FS4-LTM) in 384-well microtiter plates. The blank and solvent controls were incubated under identical conditions. After an incubation period of 5 days (for L929, KB-3-1, and MCF-7) or 24 h (for FS4-LTM), 3 μL of WST-1 (ready-to-use solution by Roche) was added. The incubation time of the plates at 37 °C varied between the cell lines from 20 min for KB-3-1, 30 min for L929, 1 h for FS4-LTM, to 2 h for MCF-7, before measuring absorbance at 450 nm (reference 600 nm) with an Infinite 200 PRO plate reader (Tecan, Männedorf, Switzerland). As the positive control compounds, Auranofin and Staurosporin were applied. The absorbance of the solvent control was set to 100%. The EC_50_ values were determined with Sigma Plot.

## 4. Conclusions

Starting from the versatile building block, pentachloro-2-nitro-1,3-butadiene (**1**), a synthetic protocol for the efficient preparation of new 2,3,4-trisubstituted benzo[*h*]quinolines **5**,**6** was developed via the intermediate **4** in a two-step process. Various modifications on quinoline **5,** such as oxidations and nucleophilic substitutions, were investigated. In the process, new benzo[*h*]quinolines **8**, **9**, **11**–**15,** and **18** with a unique substitution pattern consisting of a nucleophilic *S-*, *N-*, or *O*-unit on position 2, a nitro group on position 3, and a dichloromethyl group on position 4 of the pyridine ring were synthetized.

The phenotypic cellular assays demonstrated that some of the synthesized benzo[*h*]quinolines possess high bioactivities against mammalian cell lines and the bacterial pathogen MRSA. Although the underlying molecular mechanism or target is unknown so far, the fact that the activity is not ubiquitous across the whole series, but depends on the distinct substitution patterns found in some analogs, suggests that it is not due to unspecific effects. In addition, the overlapping, but non-parallel effects against bacteria and mammalian cells imply that it might be possible to find compounds that selectively target bacteria vs. eucaryotic cells. These findings encourage further exploration of benzo[*h*]quinolines as scaffolds in compound collections assembled for bioactivity testing. The synthetic procedure reported in this study significantly facilitates the generation of such collections.

## Figures and Tables

**Table 1:** Antibacterial and antiproliferative activities of selected benzo[*h*]quinolines.

	Antibacterial Assays		Antiproliferative Assays ^c^			
Compound	MRSA ^a^ % growth	MRSA ^b^ MIC	L929	KB-3-1	MCF-7	FS4-LTM
**6**	112.4	n.t.	n.t.	n.t.	n.t.	n.t.
**8**	1.0	17.5 (7.7)	2.5	3.8	3.5	3.4
**10d**	98.4	n.t.	>50	<0.1	>50	>50
**11a**	95.6	n.t.	>50	>50	19.0	>50
**11b**	123.3	n.t.	27.0	10.0	50.0	>50
**12**	2.0	1.2 (0.5)	3.3	3.1	>50	4.4
**13**	1.0	8.4 (3.3)	45.0	6.9	17.0	15.7
**16c**	125.6	n.t.	n.t.	n.t.	n.t.	n.t.
**17a**	119.6	n.t.	n.t.	n.t.	n.t.	n.t.
**17b**	112.0	n.t.	n.t.	n.t.	n.t.	n.t.
**17c**	101.1	n.t.	n.t.	n.t.	n.t.	n.t.
**18a**	95.1	n.t.	n.t.	n.t.	n.t.	n.t.
**18b**	107.0	n.t.	n.t.	n.t.	n.t.	n.t.
**18c**	110.5	n.t.	n.t.	n.t.	n.t.	n.t.
**Linezolid**	1.0	0.4	n.t.	n.t.	n.t.	n.t.
**Staurosporine**	n.t.	n.t.	<0.05	<0.05	<0.14	0.44
**Auranofin**	n.t.	n.t.	0.4	0.6	3.5	1.3

^a^ Strain DSM 11822, % growth at a compound concentration of 5 µM; ^b^ strain RKI 11-02670, minimal inhibitory concentrations in µM (µg/mL values in brackets); ^c^ EC_50_ values in µM.

## Data Availability

Not applicable.

## Supplementary Materials

The following supporting information can be downloaded at https://www.mdpi.com/article/10.3390/molecules28062479/s1, Figures S1–S27: ^1^H-NMR and ^13^C-NMR; Figures S28–S52: HR-MS; Figures S53–S64: X-ray–Supplementary Material.

## Sample Availability

Samples of all compounds are available from the authors (V.A.Z.).
