# Supplementary material for: A New Way to 2,3,4-Trisubstituted Benzo[h]quinolines: Synthesis, Consecutive Reactions and Cellular Activities
Source: Molecules. 2023 Mar 8;28(6):2479. doi: 10.3390/molecules28062479 (PMC10058827; doi:10.3390/molecules28062479)

Supplementary Material

Contents

1. Copies of ^{1}H-NMR and ^{13}C-NMR spectra for newly synthesized compounds (Figures S1 – S27)....…………….…………………………………………………………………..S2
2. Copies of HR-MS spectra for newly synthesized compounds (Figures S28 – S52) ……………………………………………..……………………………..S29
3. X-Ray - Supplementary Material (Figures S53 – S64)……………..…………………..…………………..………………………….. S54

Figure S1. ^{1}H and ^{13}C NMR spectra of compound **4a**

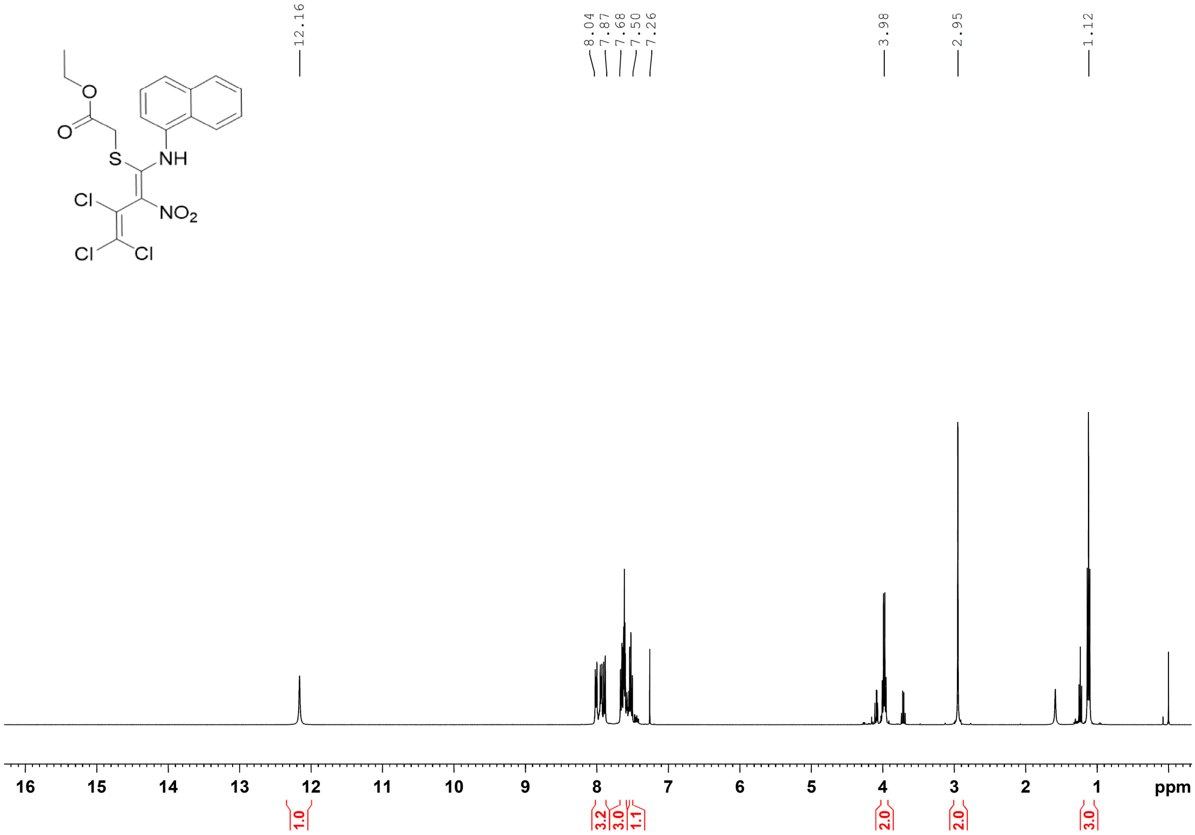

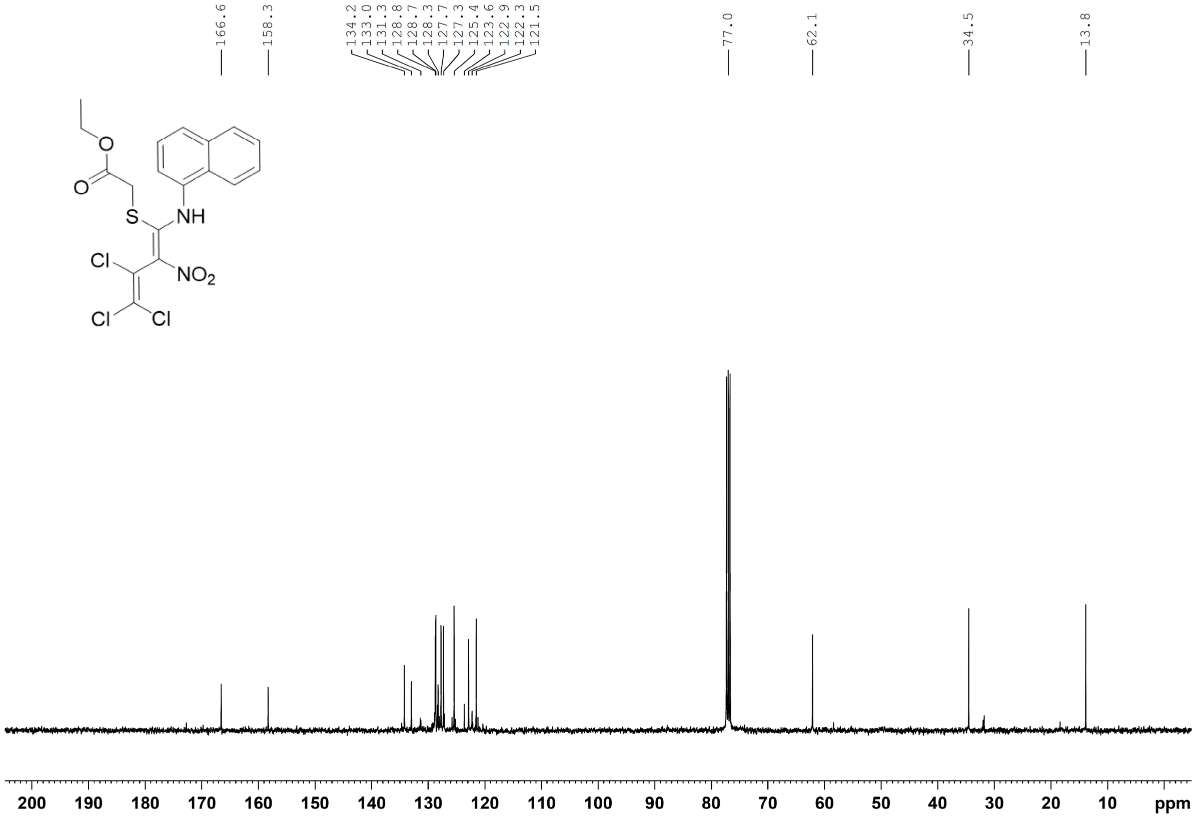

Figure S2. ^{1}H and ^{13}C NMR spectra of compound **4b**

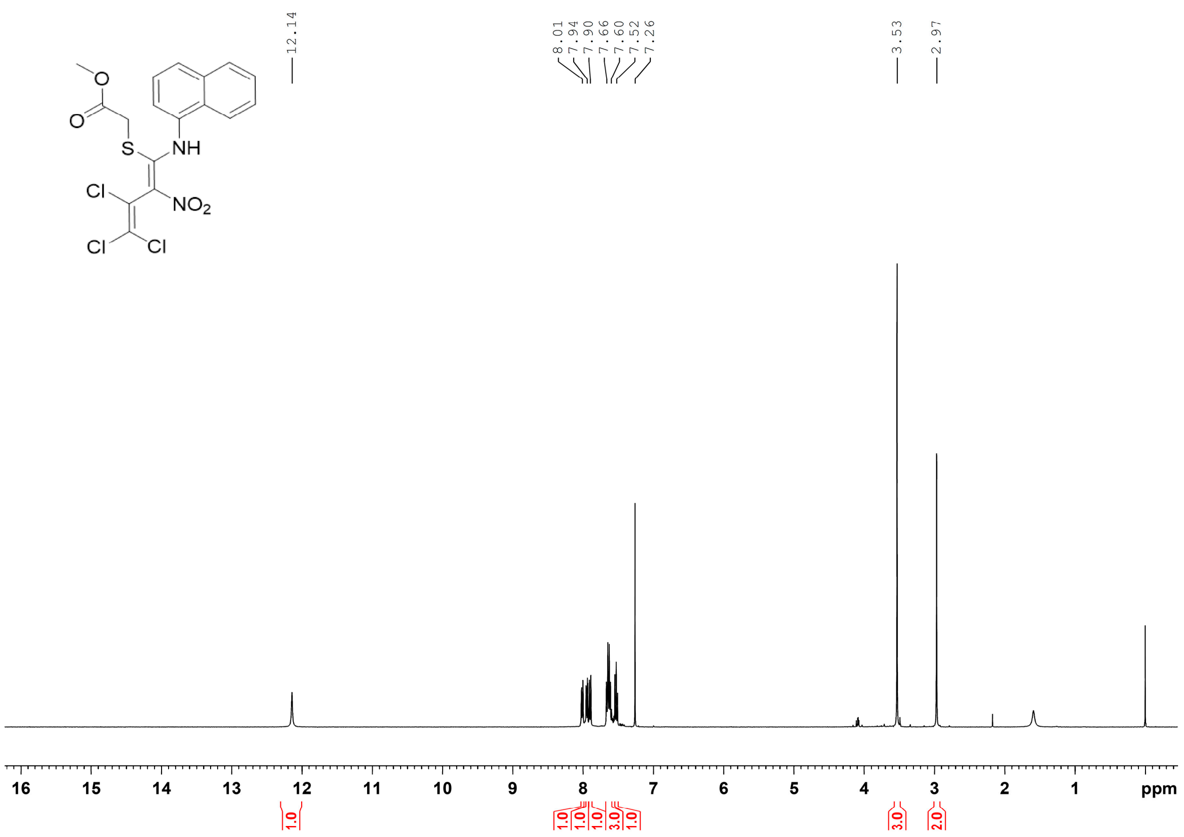

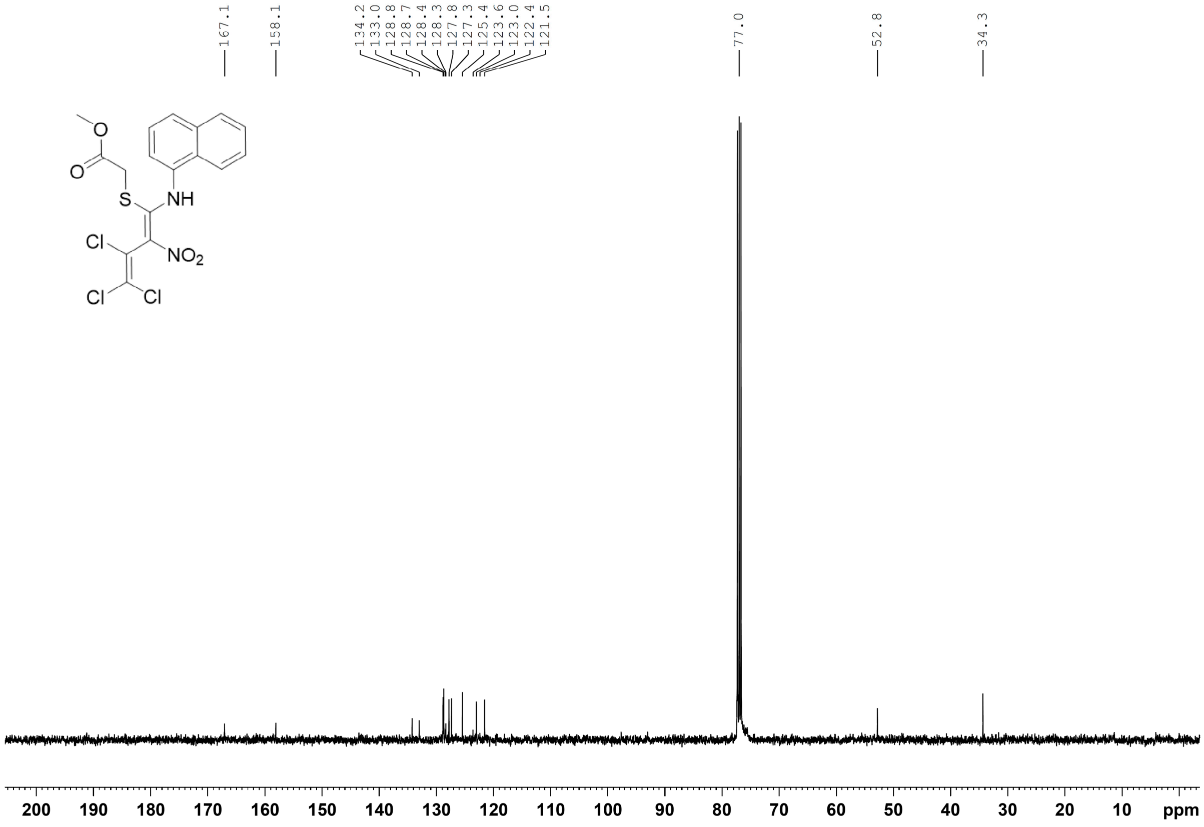

Figure S3. ^{1}H and ^{13}C NMR spectra of compound **5a**

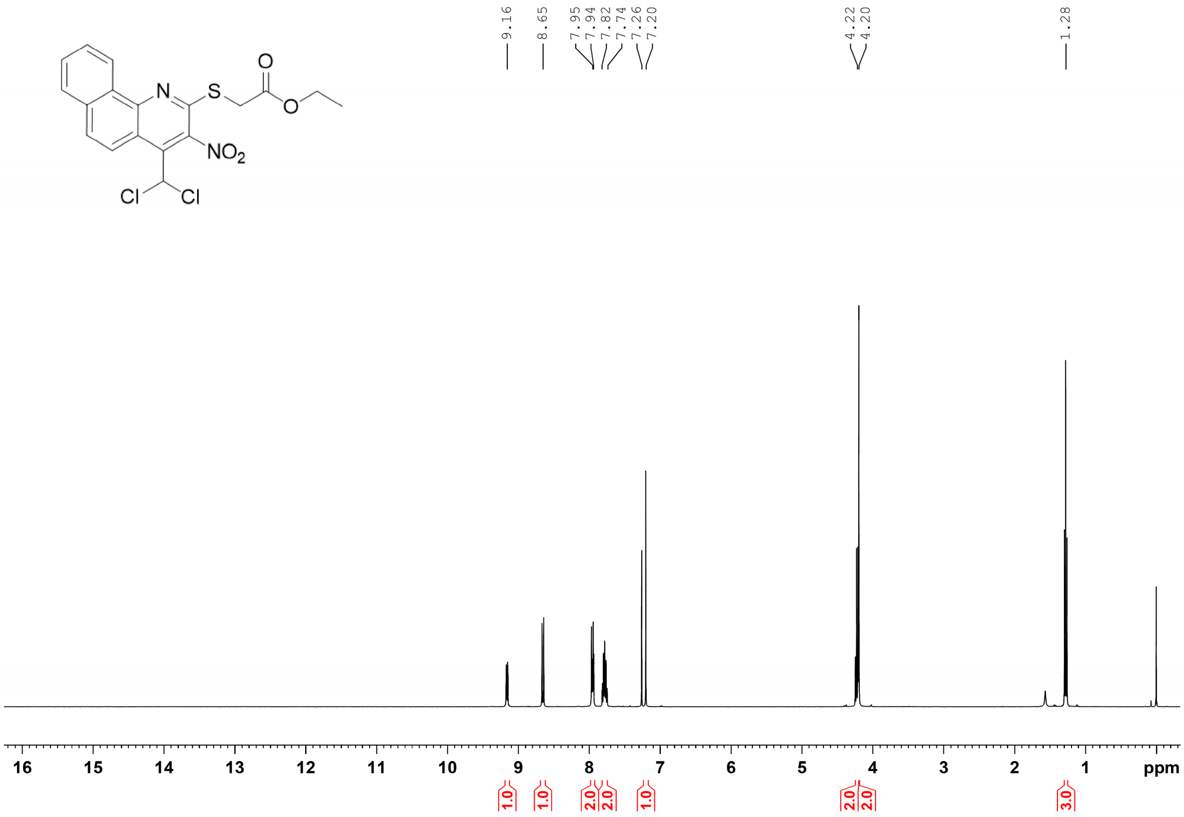

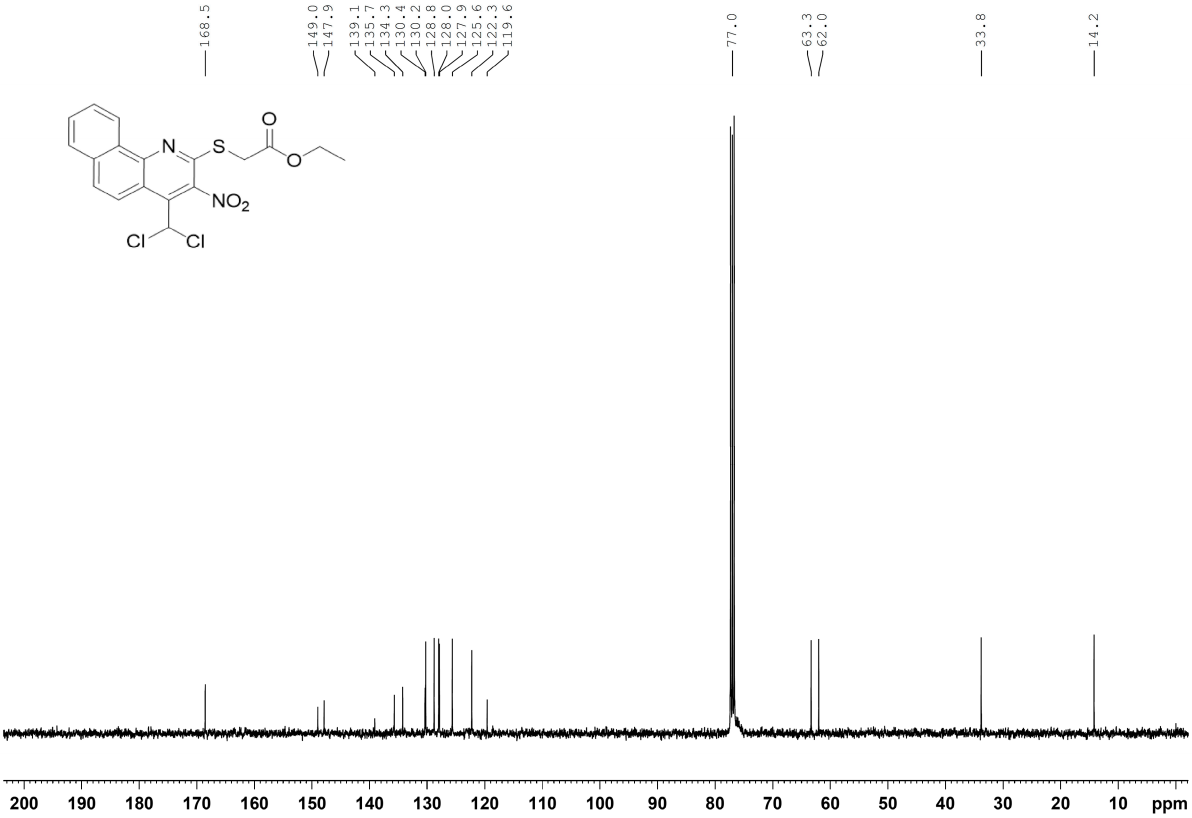

Figure S4. ^{1}H and ^{13}C NMR spectra of compound **5b**

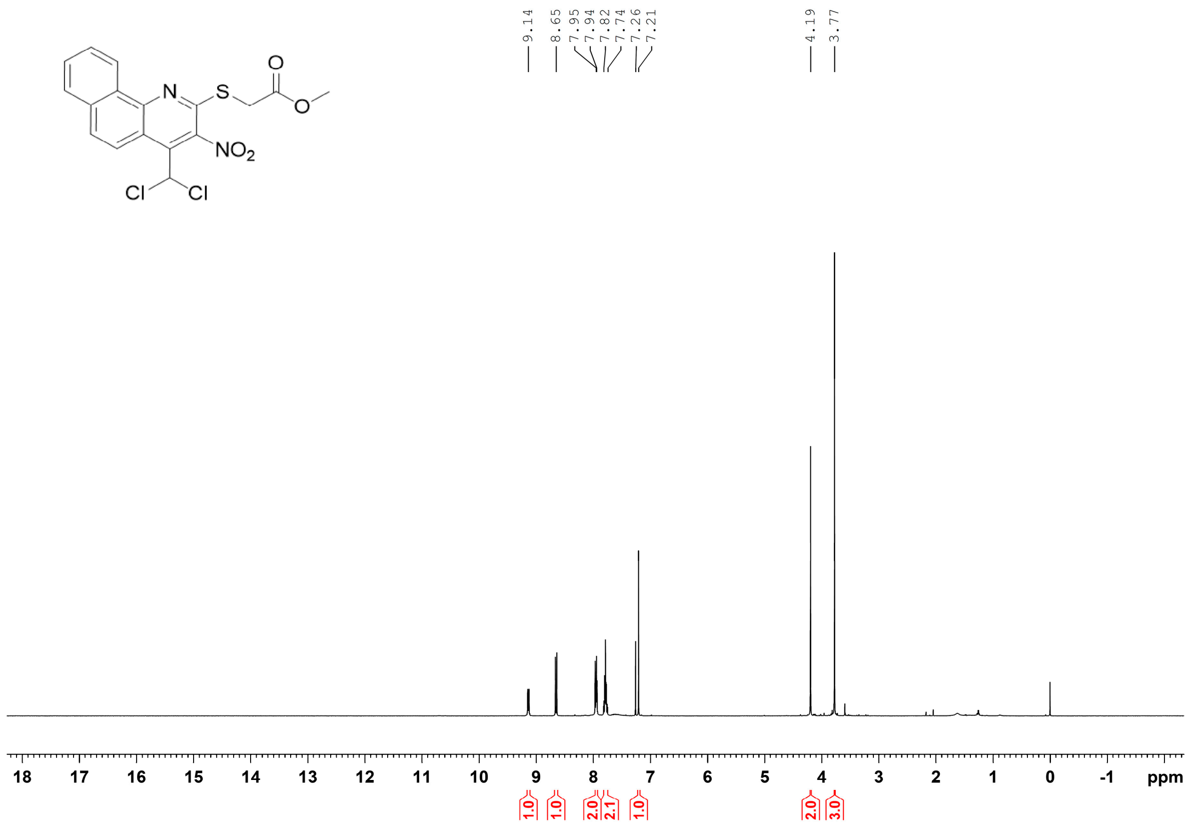

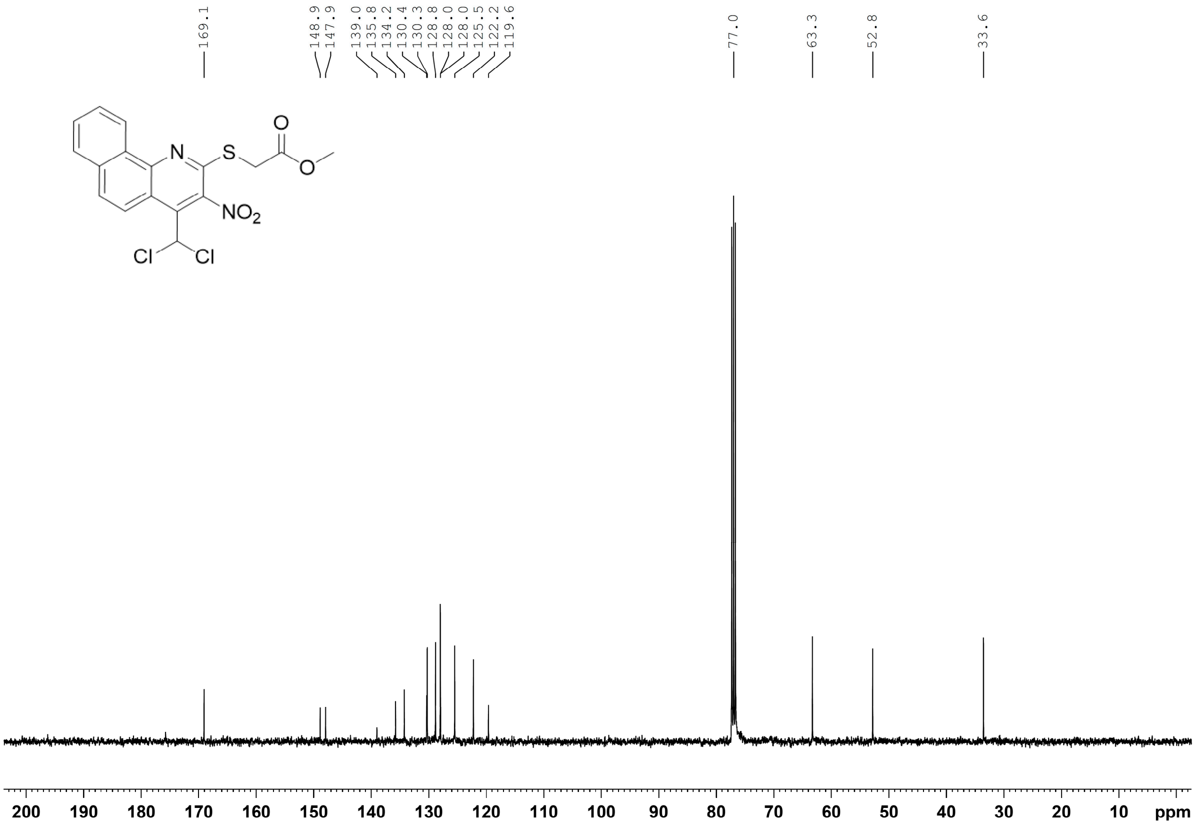

Figure S5. ^{1}H and ^{13}C NMR spectra of compound **6**

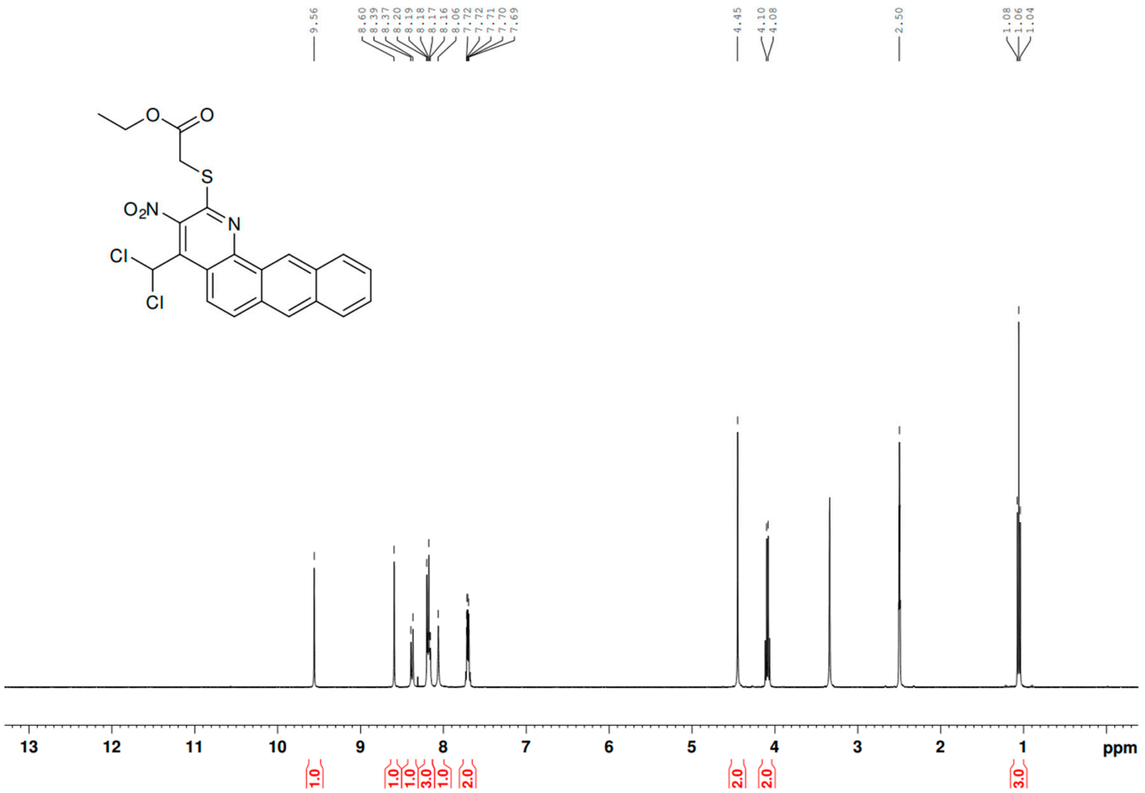

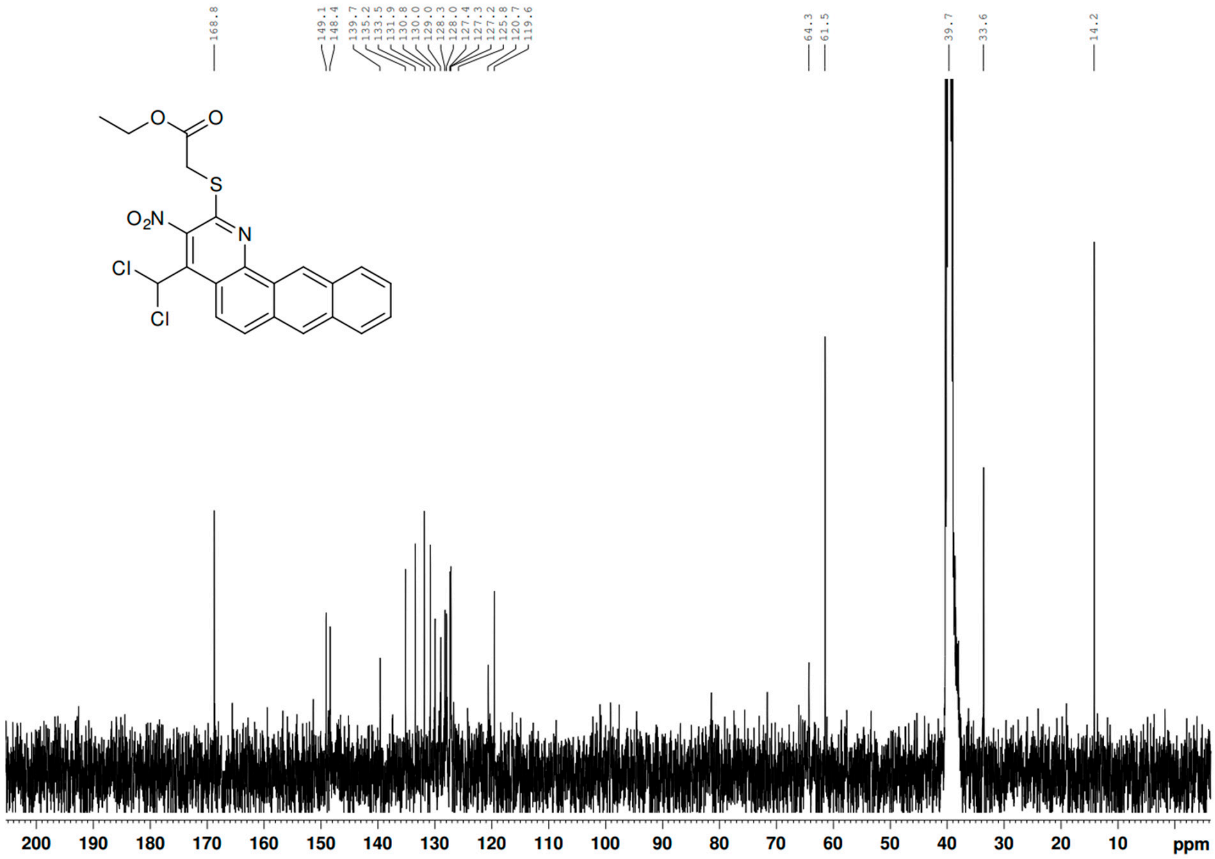

Figure S6. ^{1}H and ^{13}C NMR spectra of compound **7a**

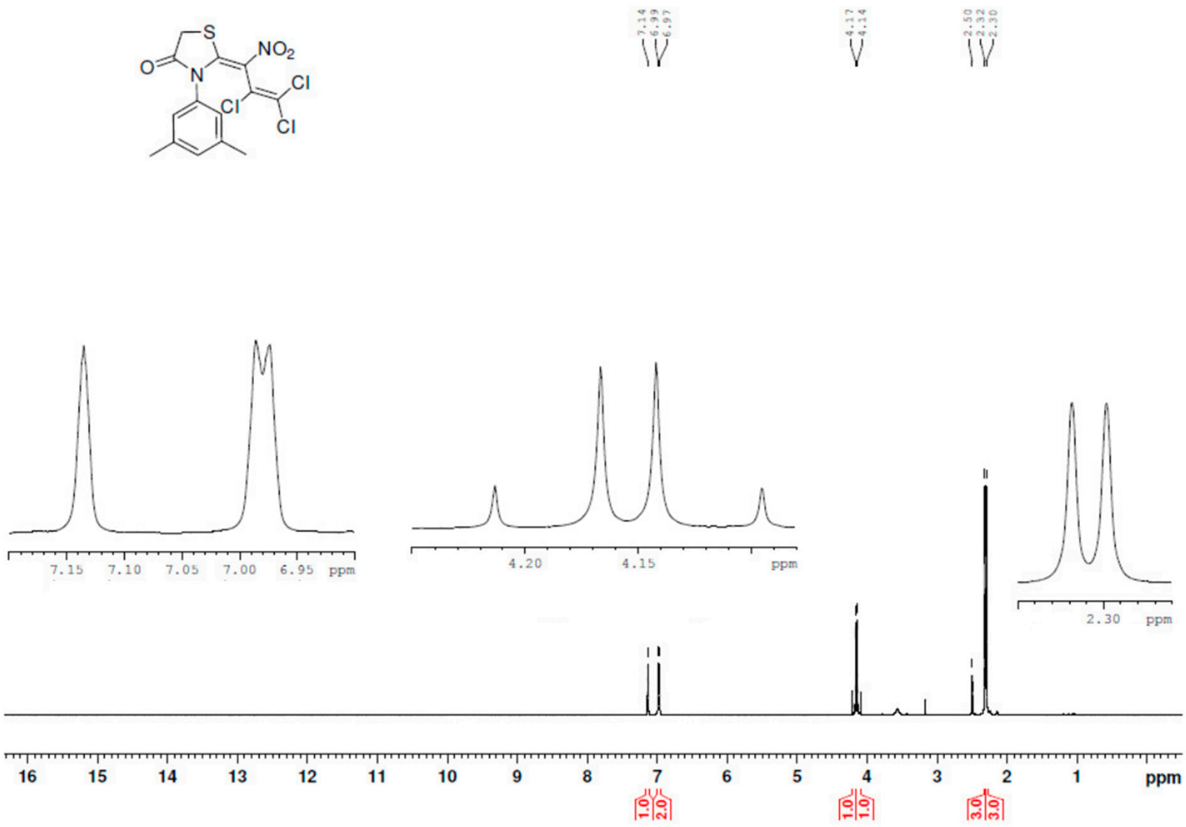

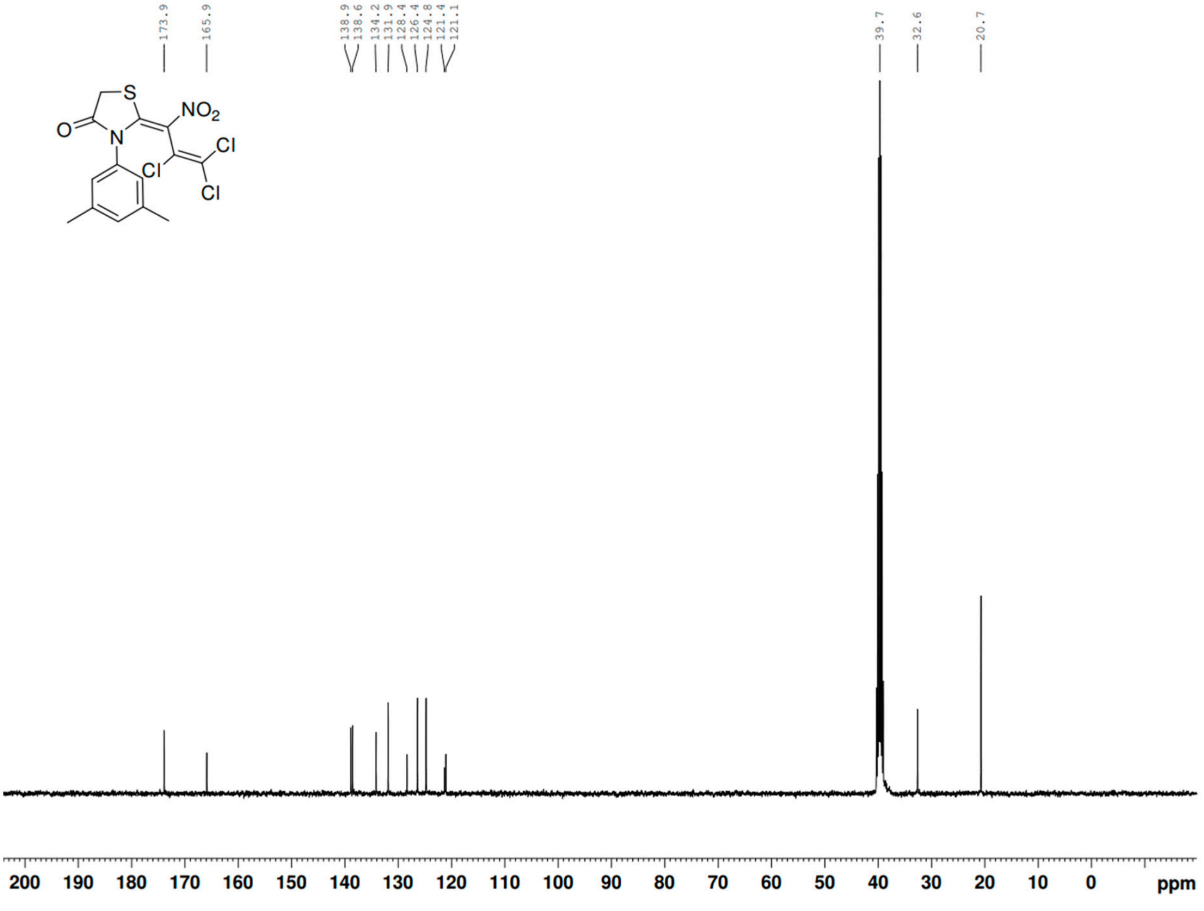

Figure S7. ^{1}H and ^{13}C NMR spectra of compound **7b**

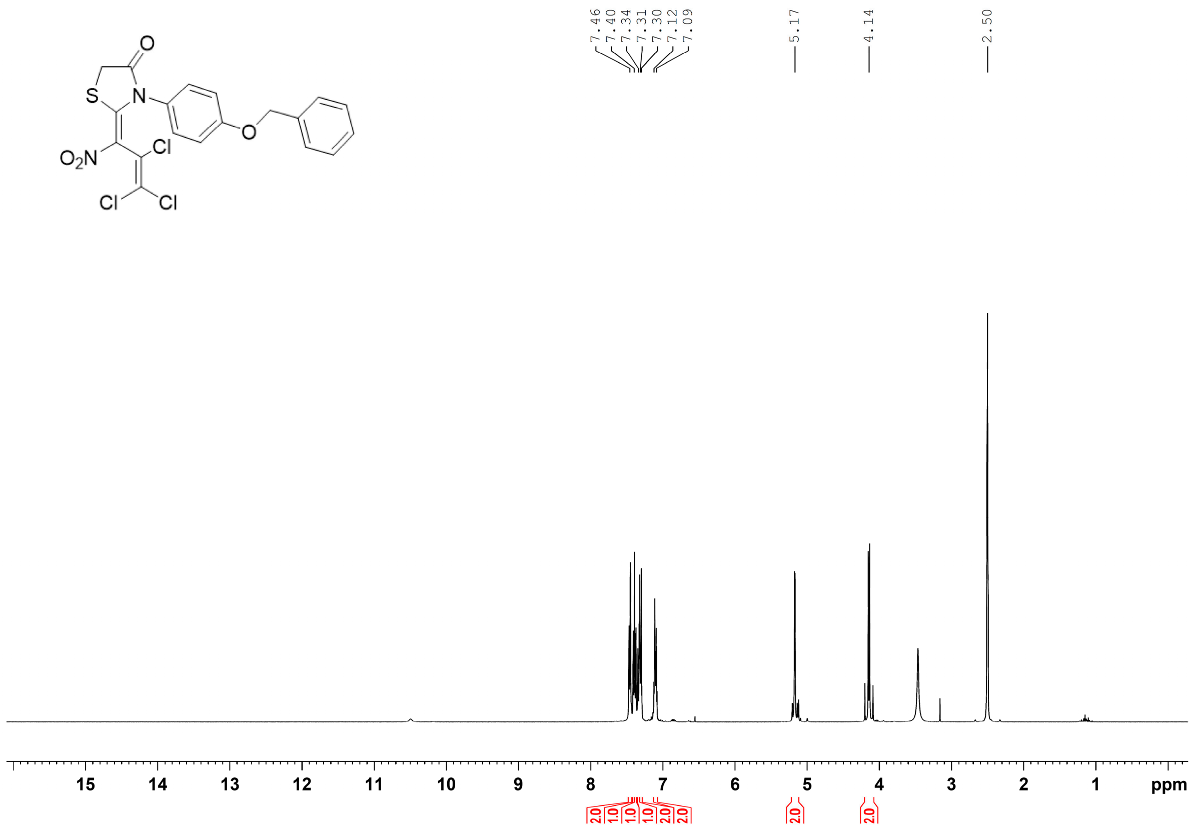

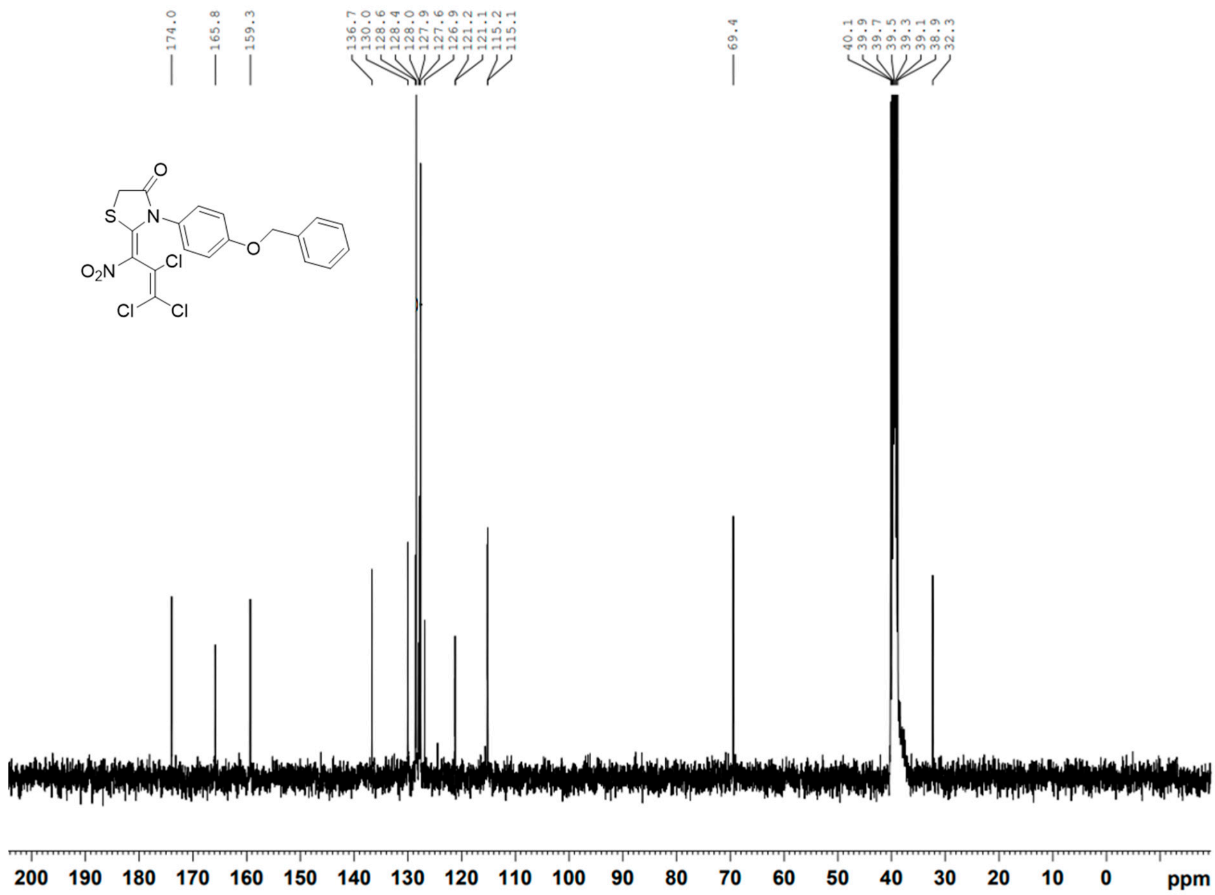

Figure S8. ^{1}H and ^{13}C NMR spectra of compound **7c**

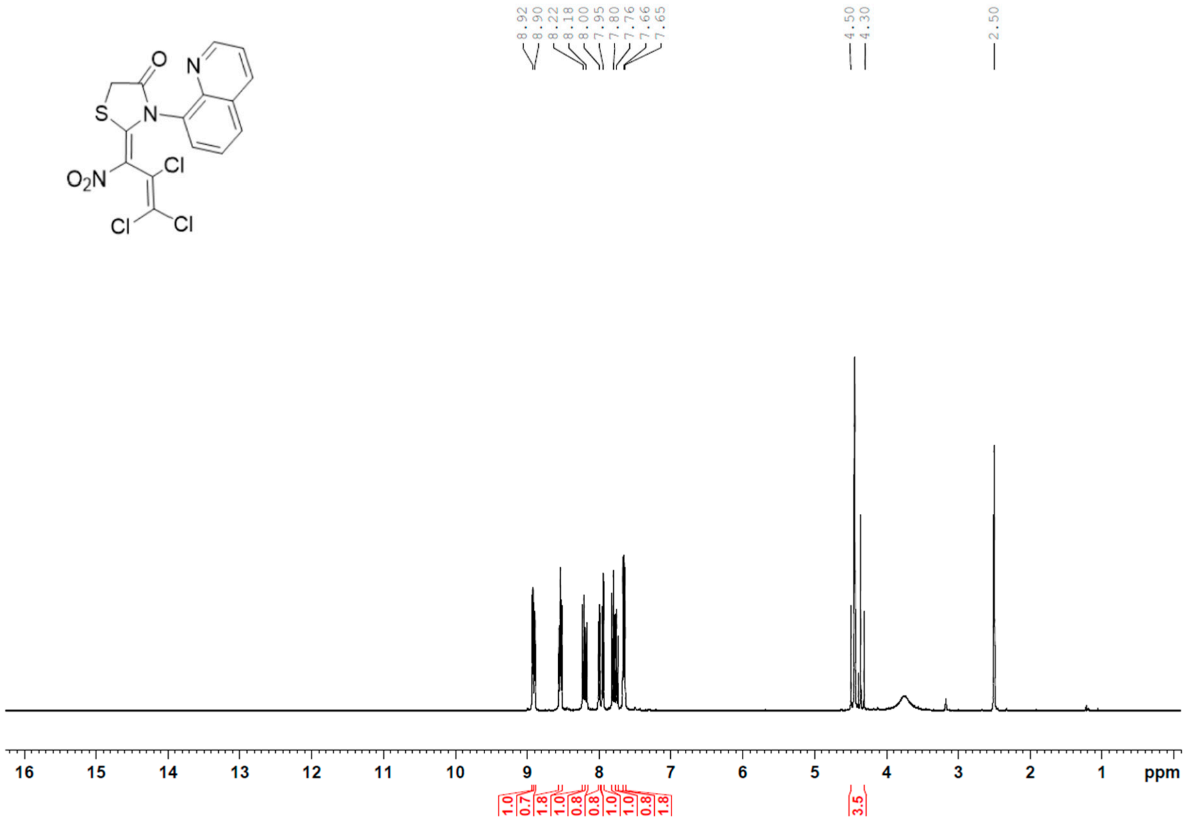

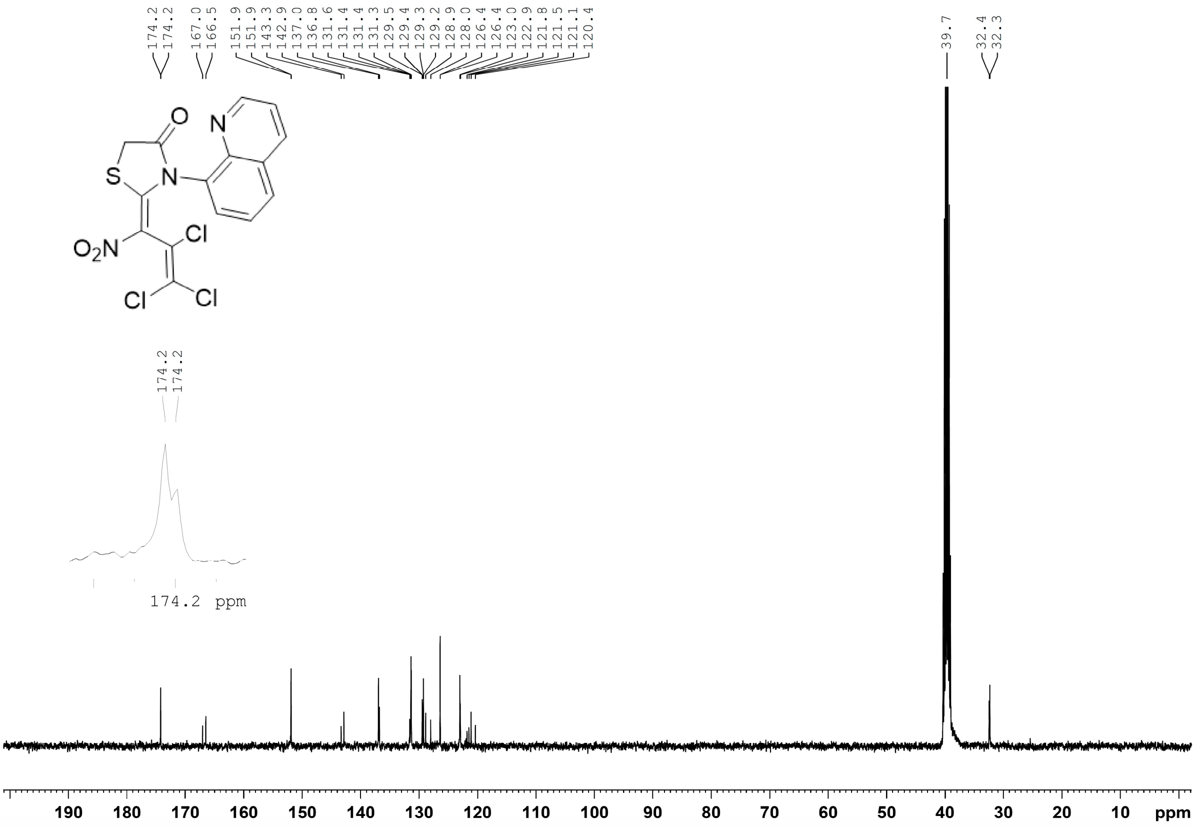

Figure S9. ^{1}H and ^{13}C NMR spectra of compound **8**

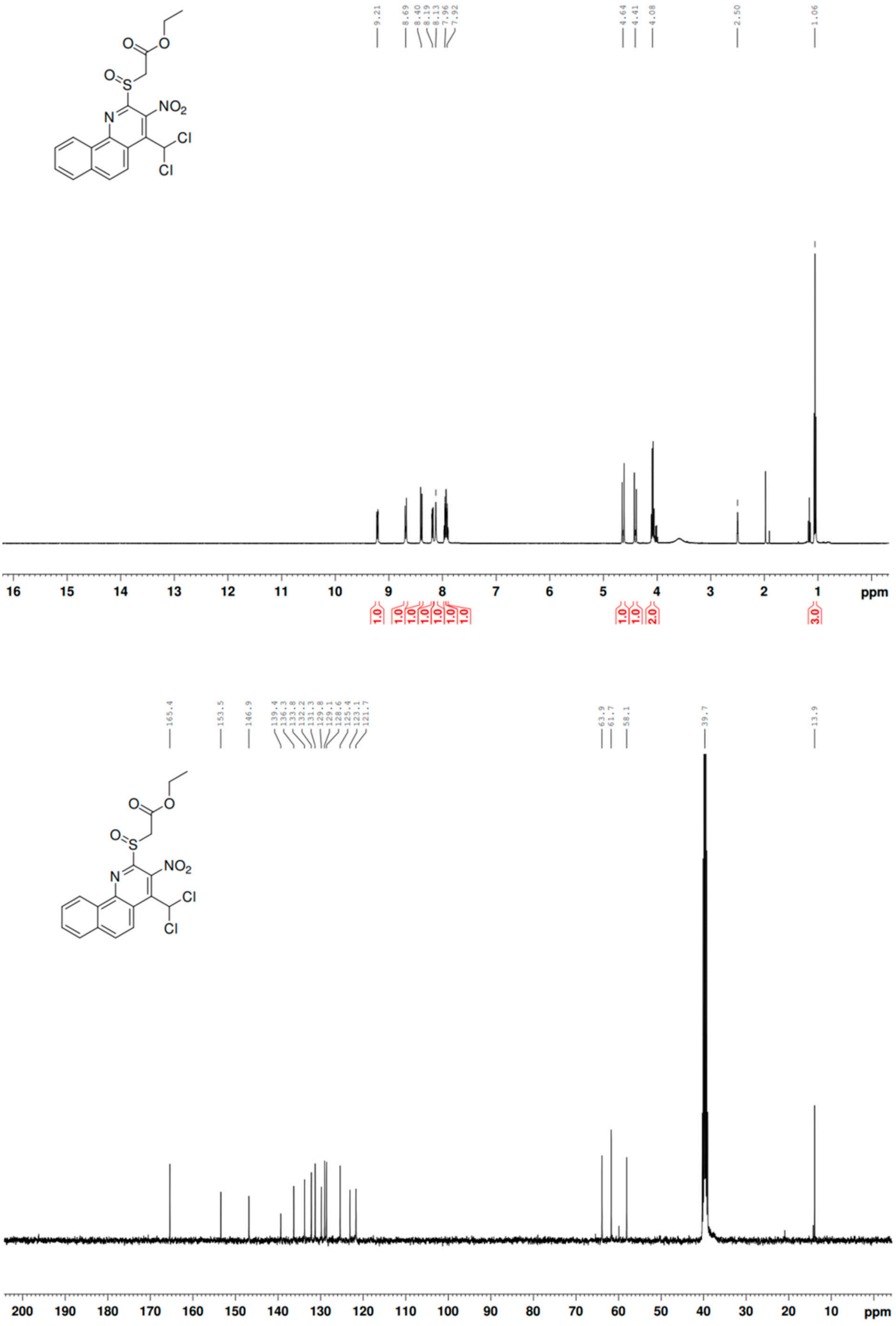

Figure S10. ^{1}H and ^{13}C NMR spectra of compound **9**

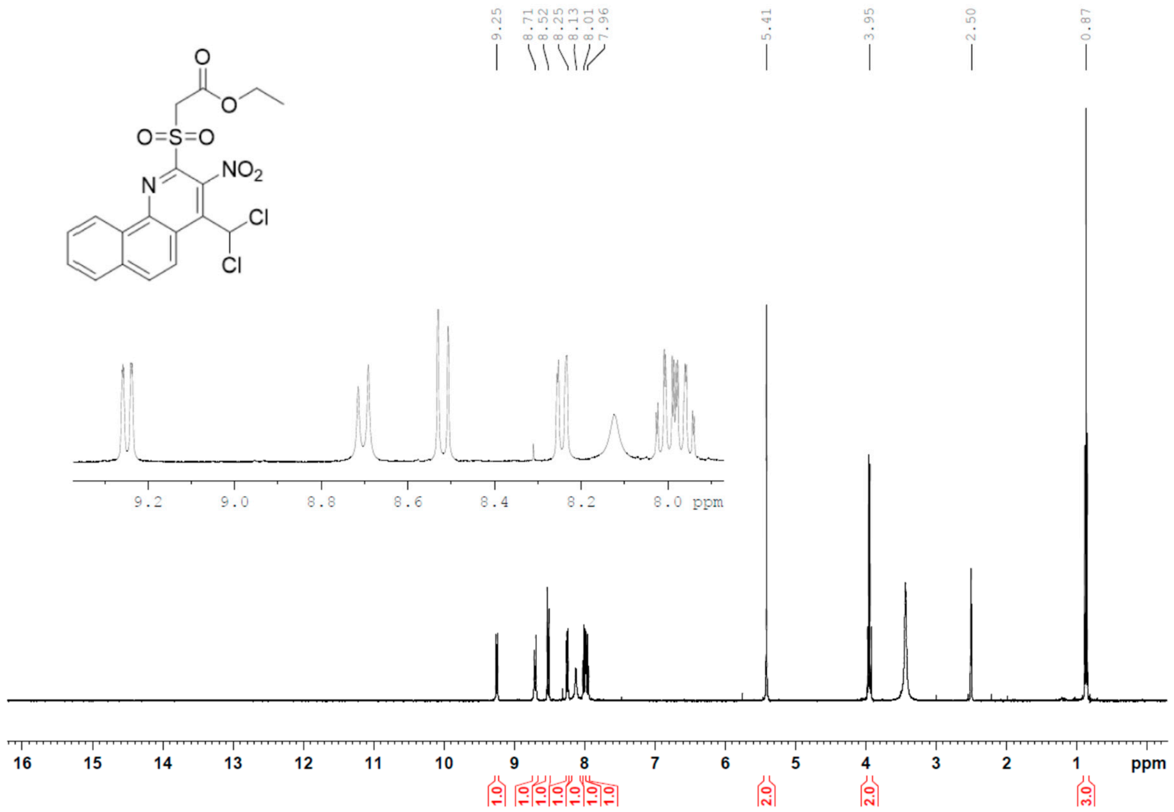

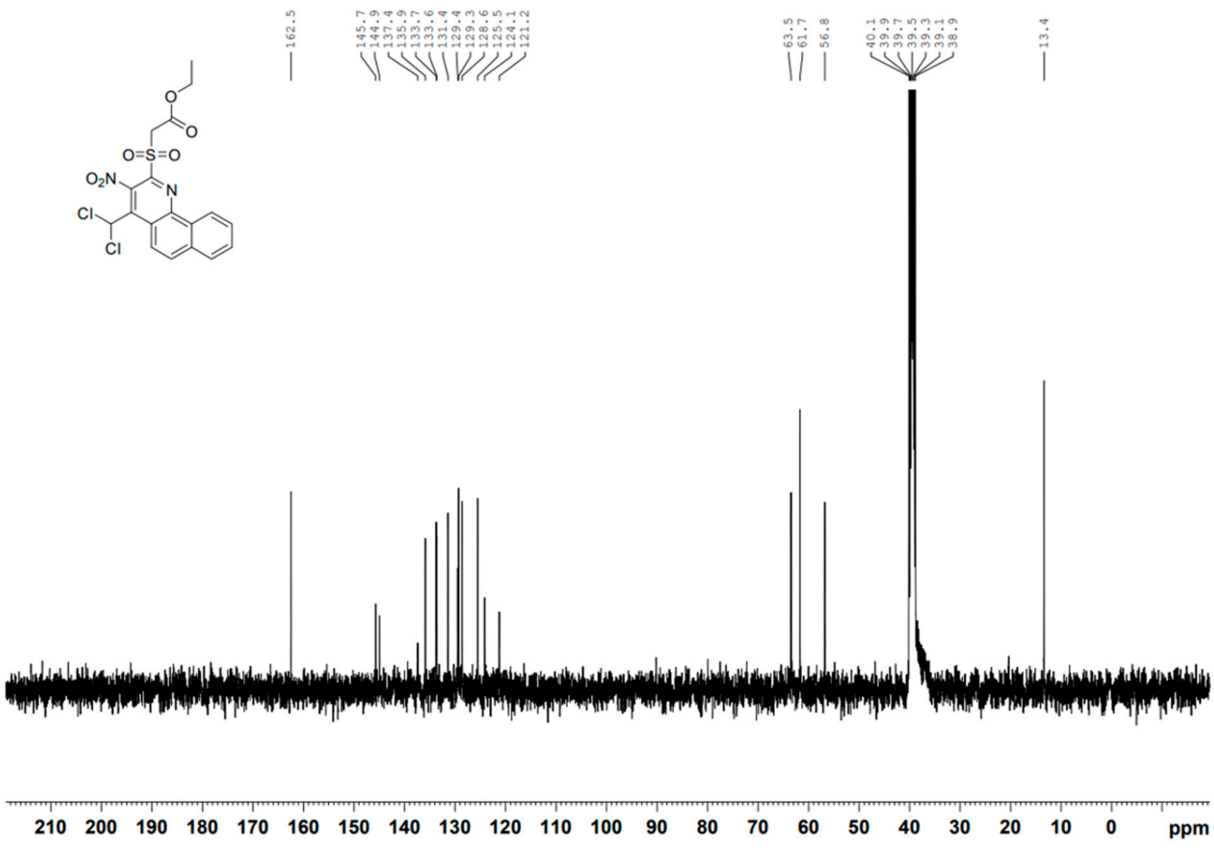

Figure S11. ^{1}H and ^{13}C NMR spectra of compound **10a**

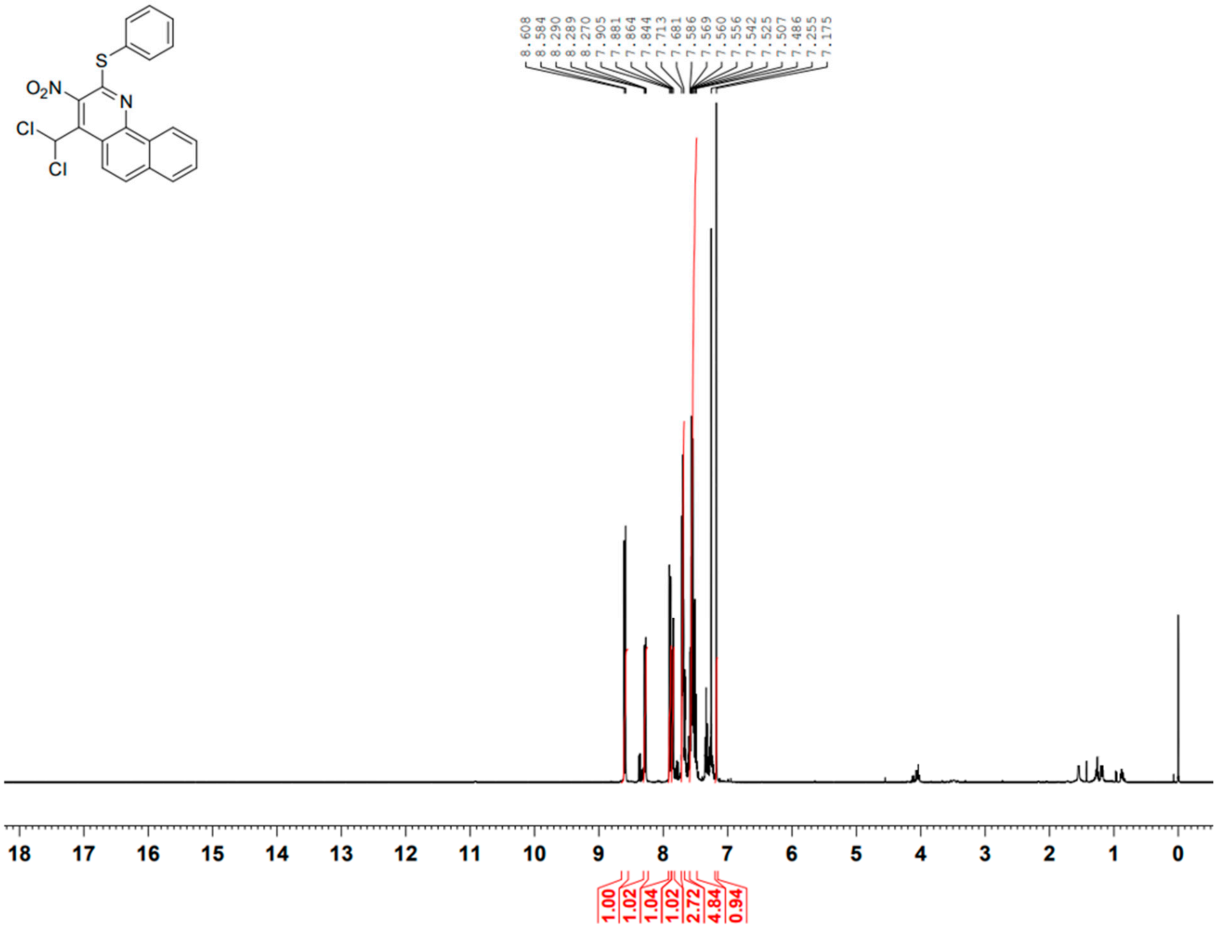

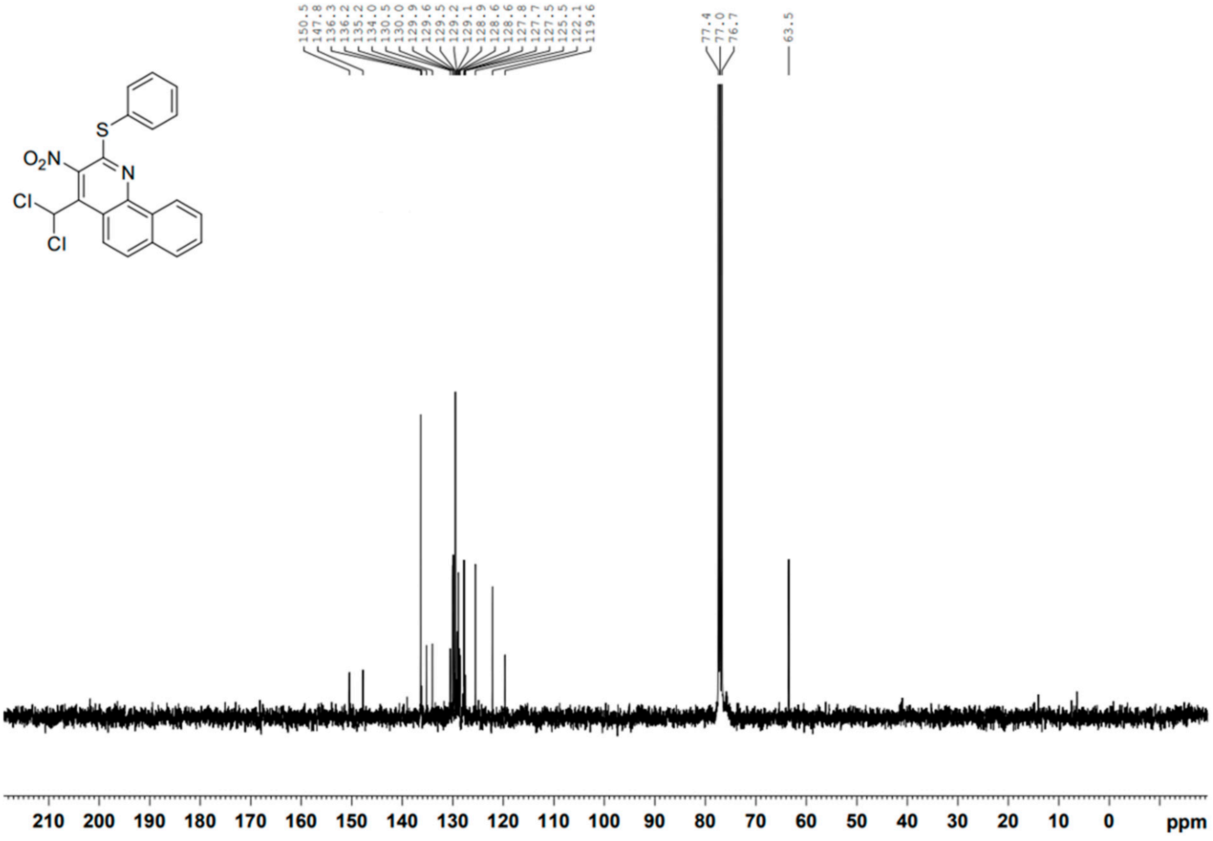

Figure S12. ^{1}H and ^{13}C NMR spectra of compound **10b**

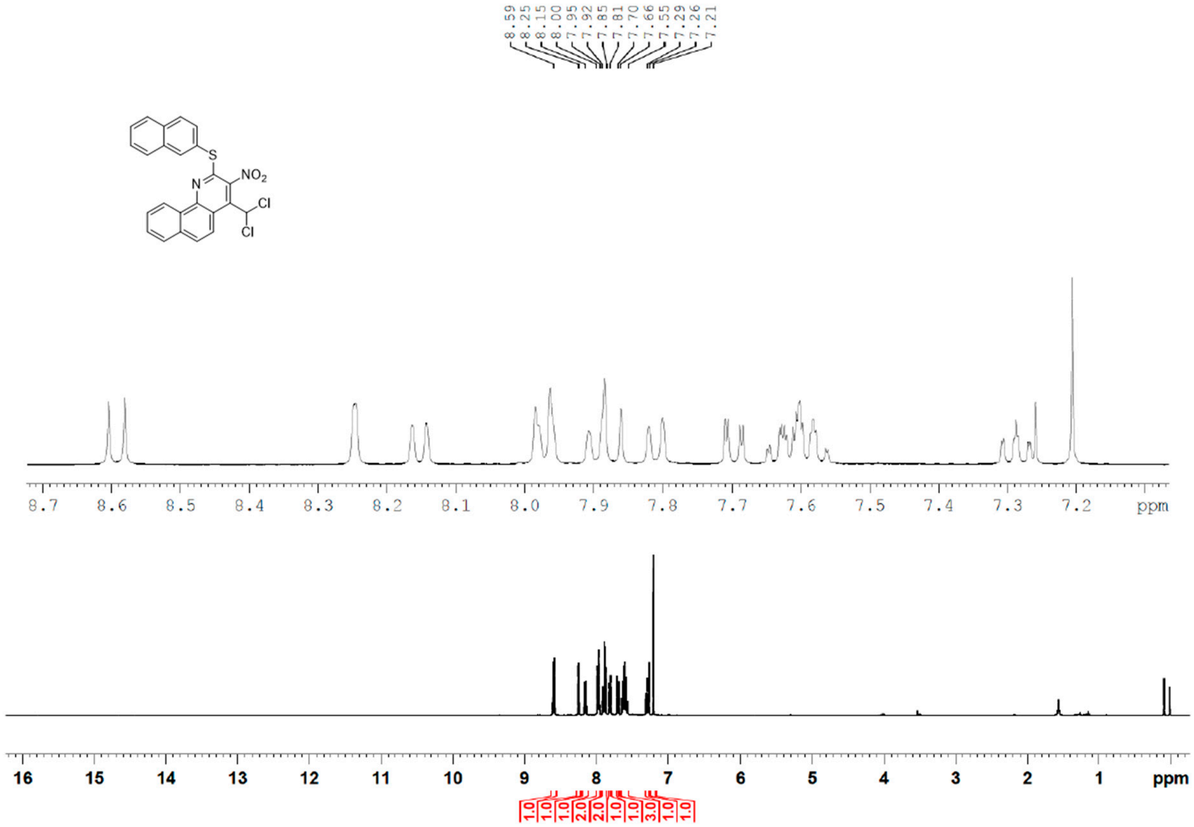
8.59
8.25
8.15
8.00
7.95
7.92
7.85
7.81
7.70
7.66
7.55
7.29
7.26
7.21
S
NO2
N
Cl
Cl
ppm

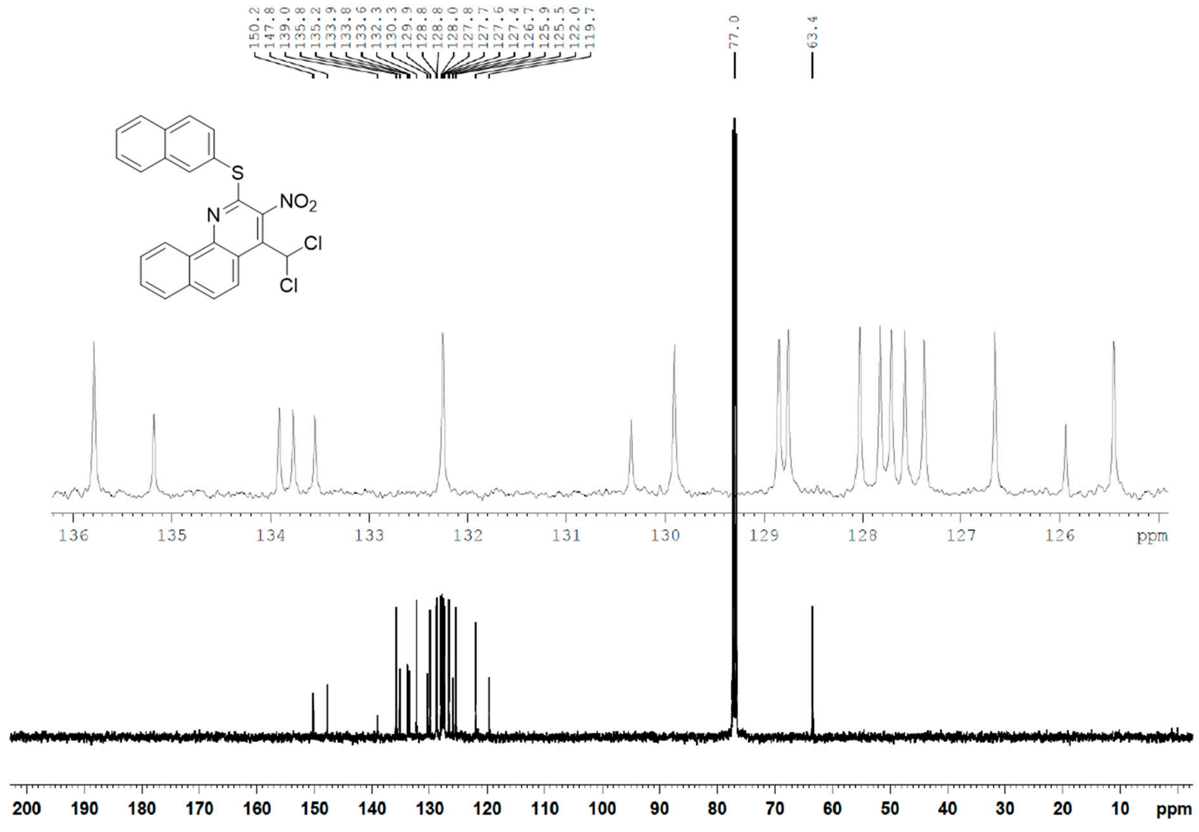
150.2
147.8
139.0
135.8
135.2
133.9
133.8
133.6
132.3
130.3
129.9
128.8
128.8
128.0
127.8
127.7
127.6
127.4
126.7
125.9
125.5
122.0
119.7
77.0
63.4
S
NO2
N
Cl
Cl
ppm

Figure S13. ^{1}H and ^{13}C NMR spectra of compound **10c**

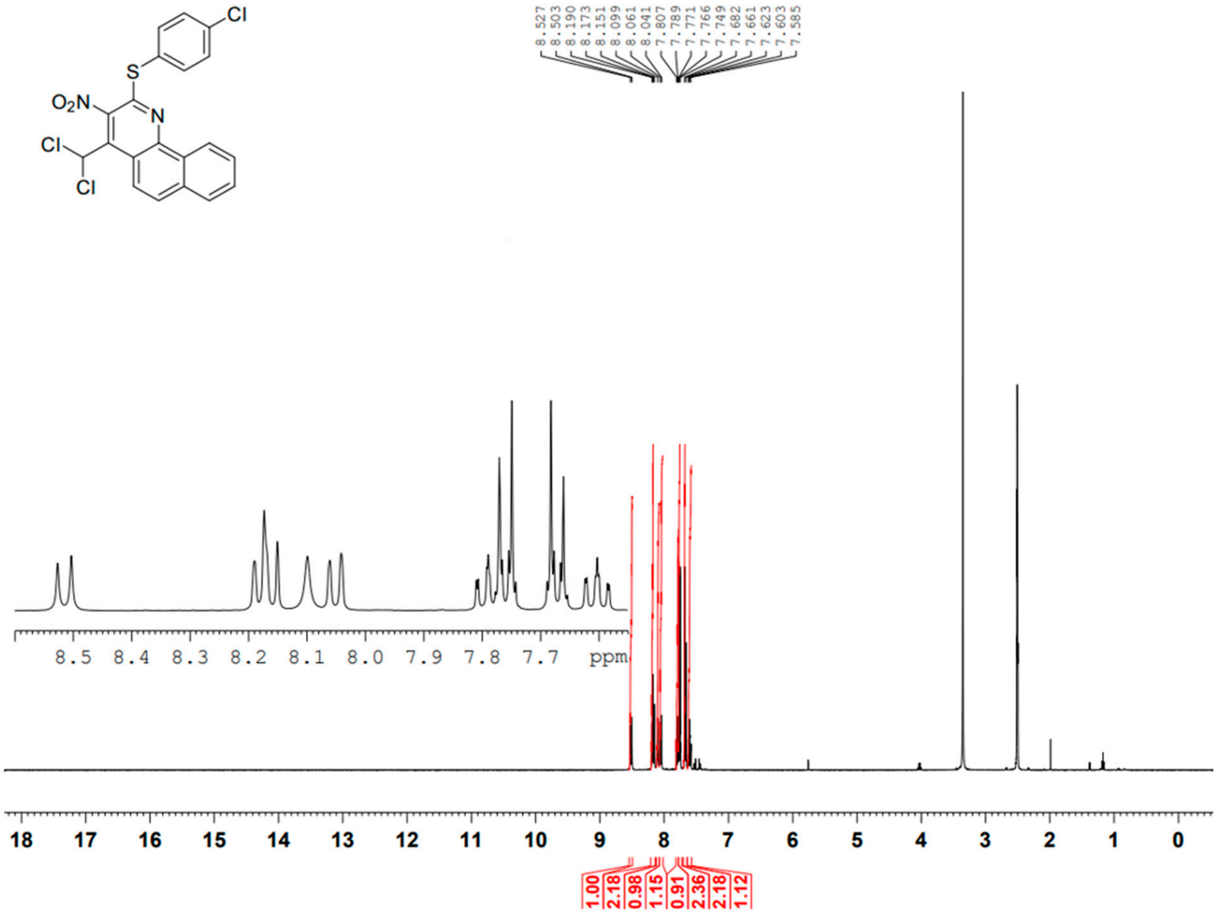

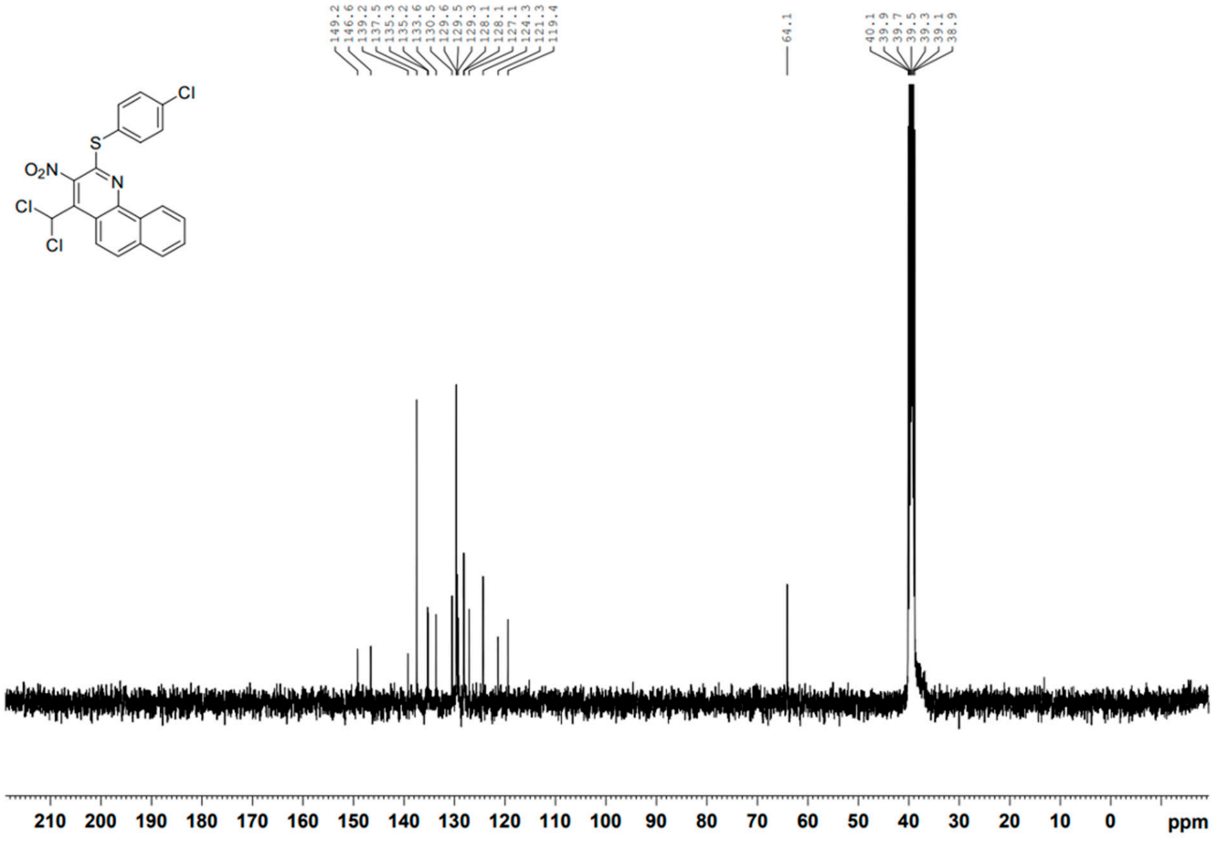

Figure S14. ^{1}H and ^{13}C NMR spectra of compound **10d**

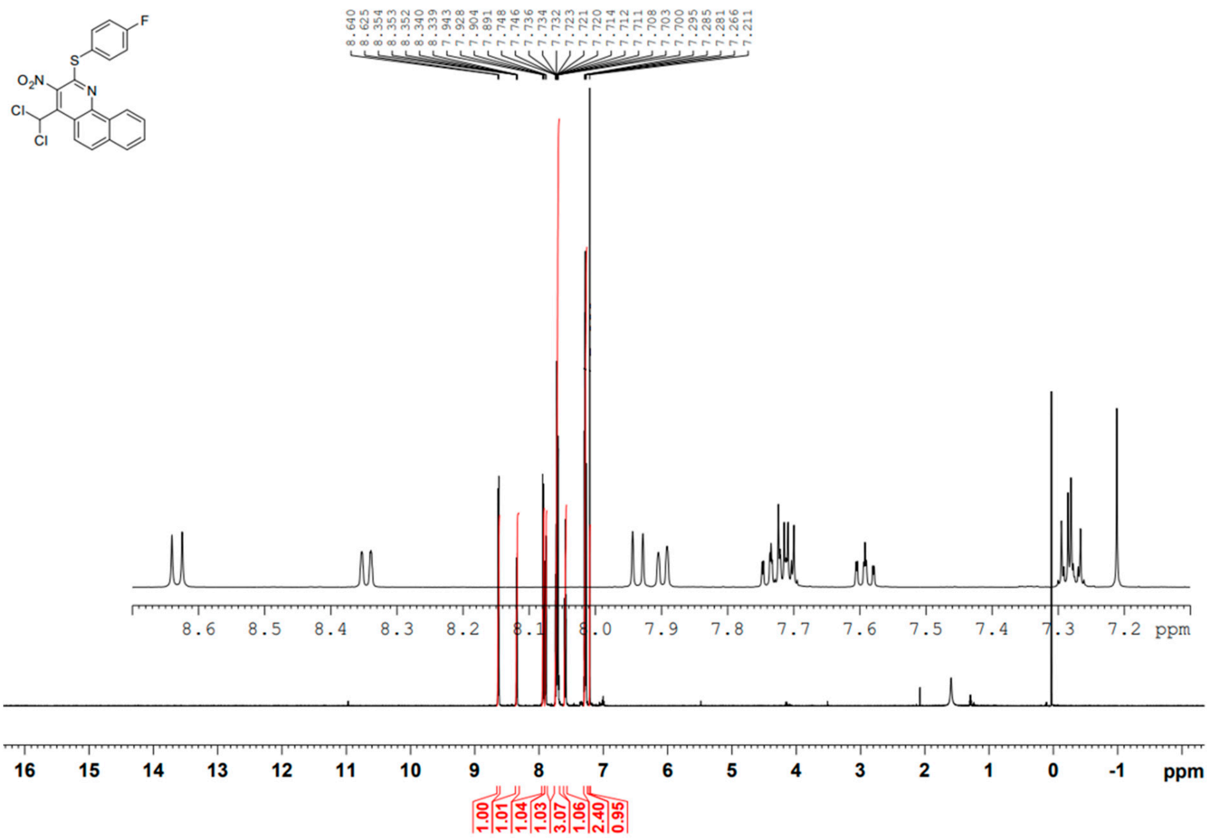

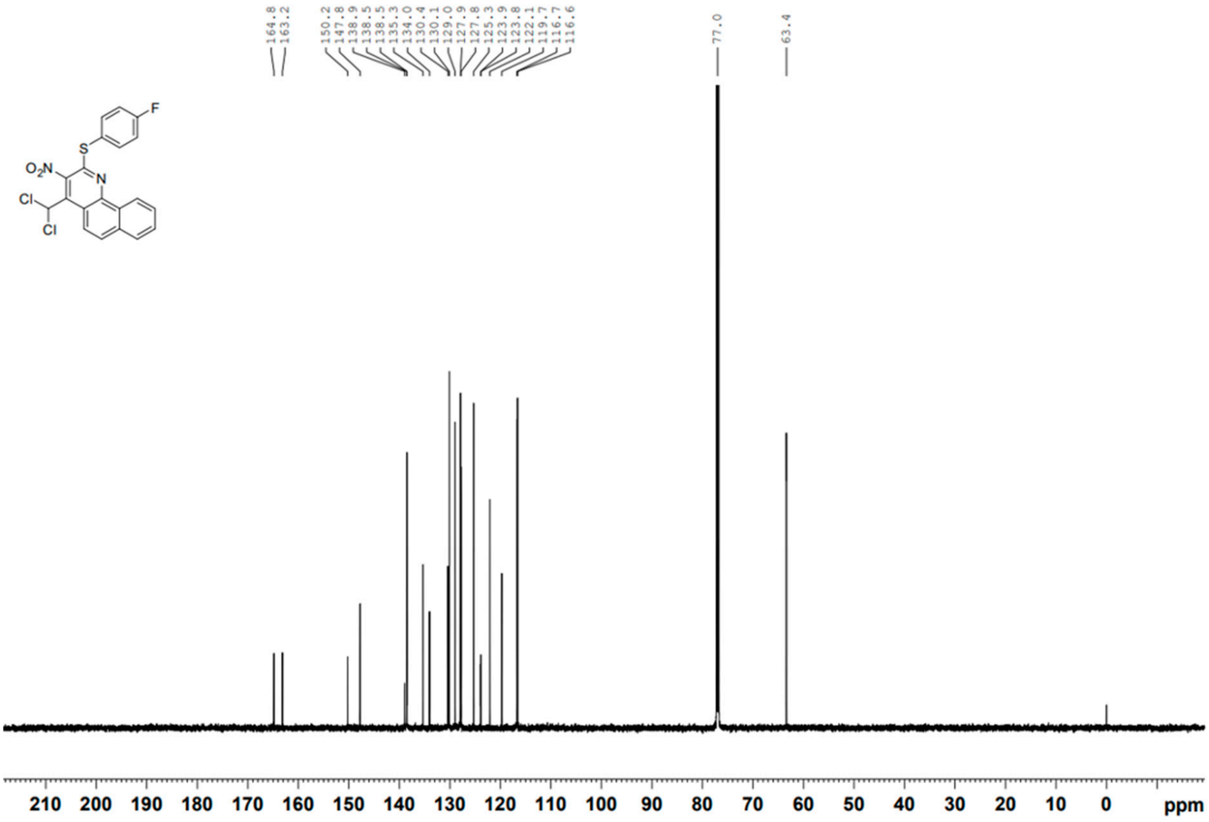

Figure S15. ^{1}H and ^{13}C NMR spectra of compound **11a**

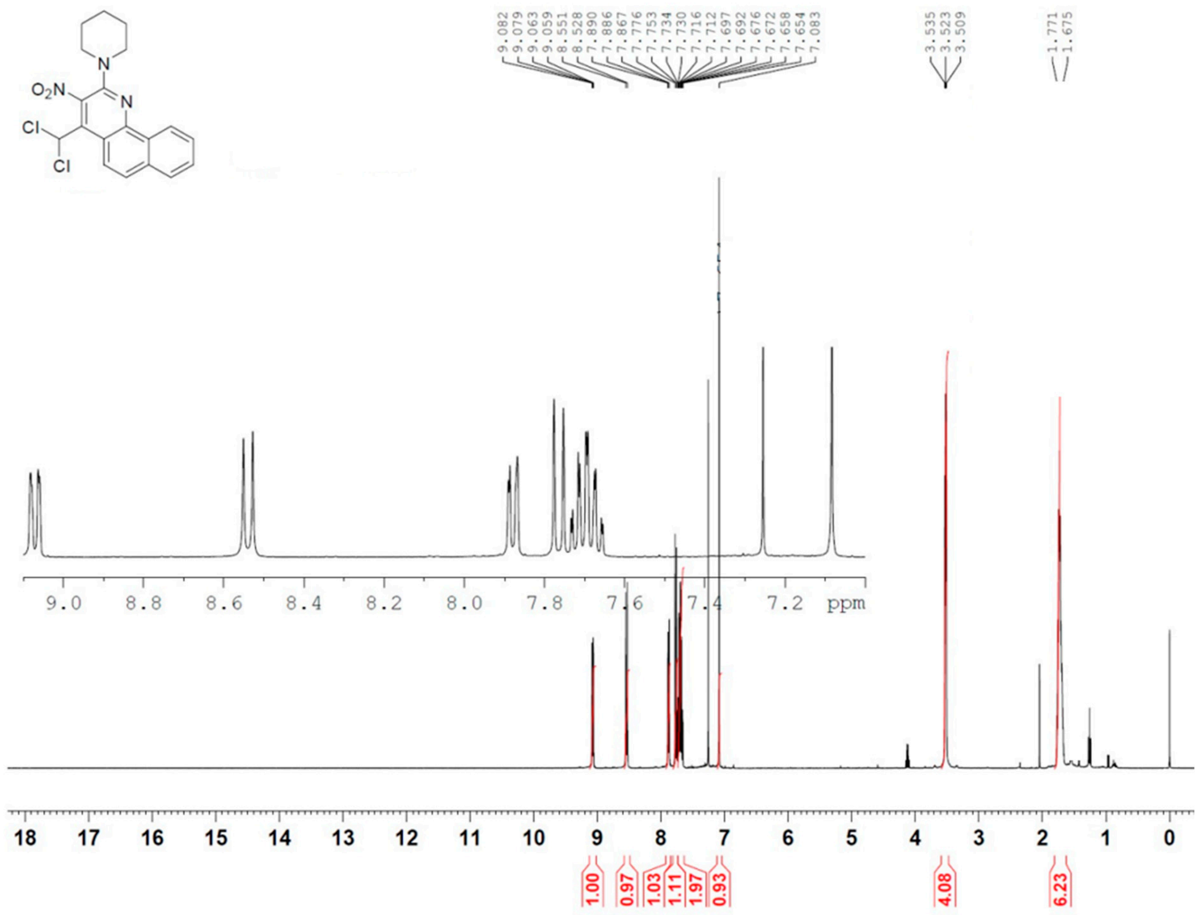

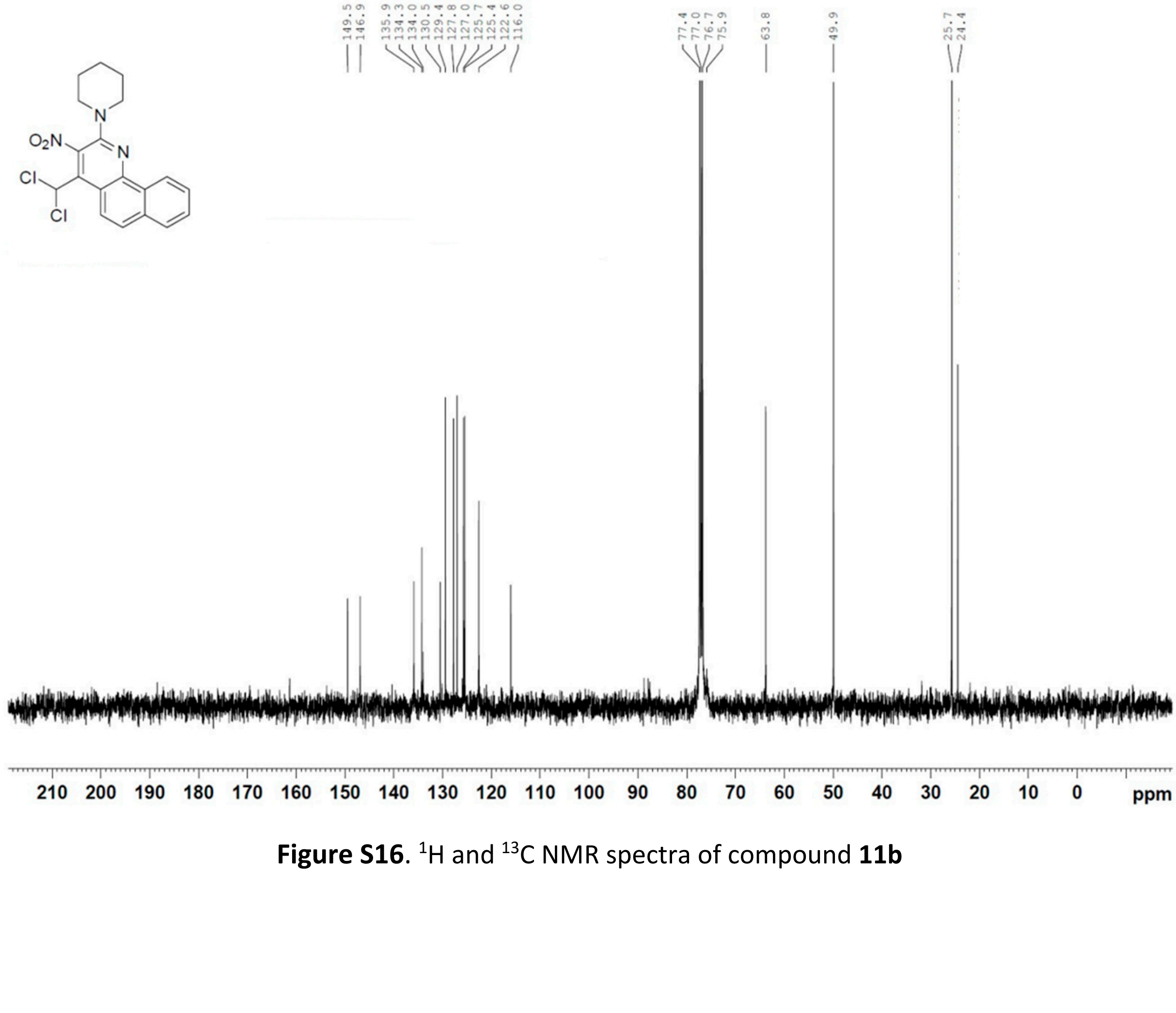

Figure S16. ^{1}H and ^{13}C NMR spectra of compound **11b**

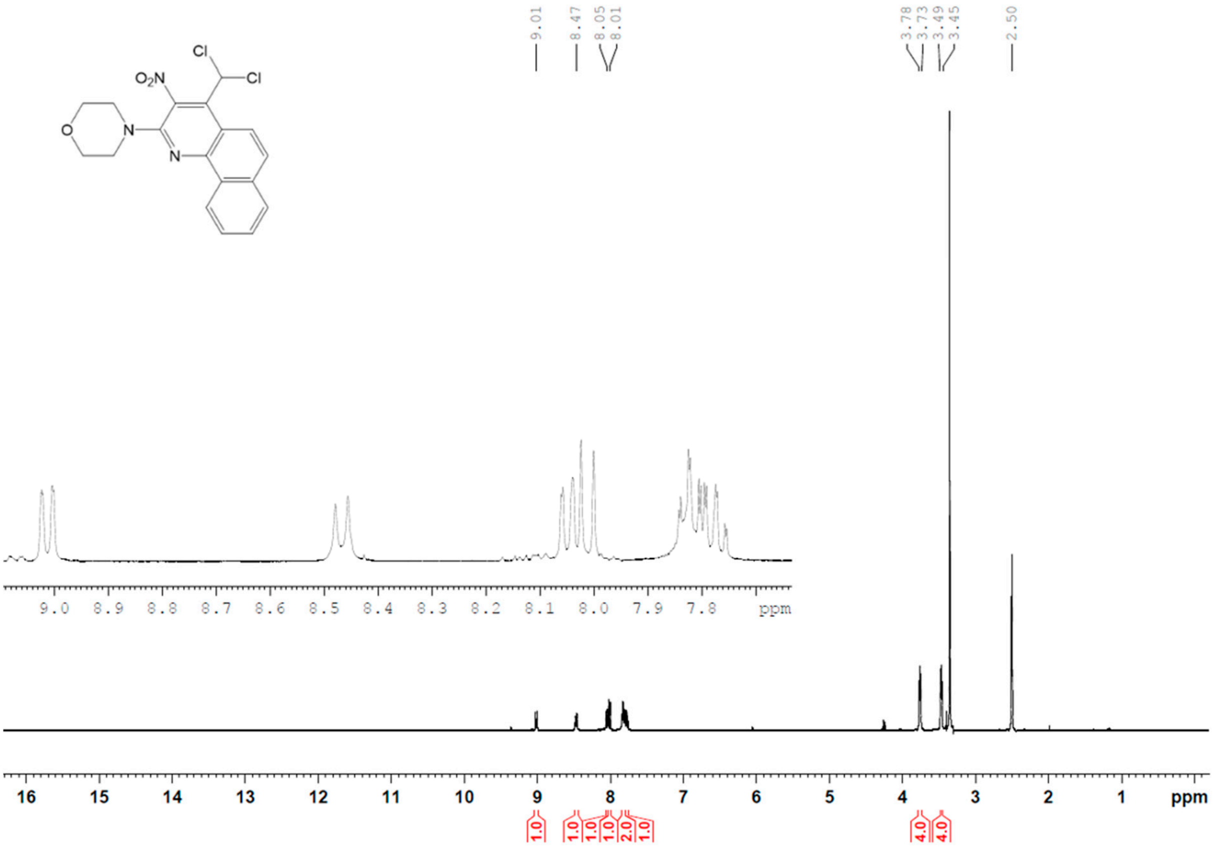

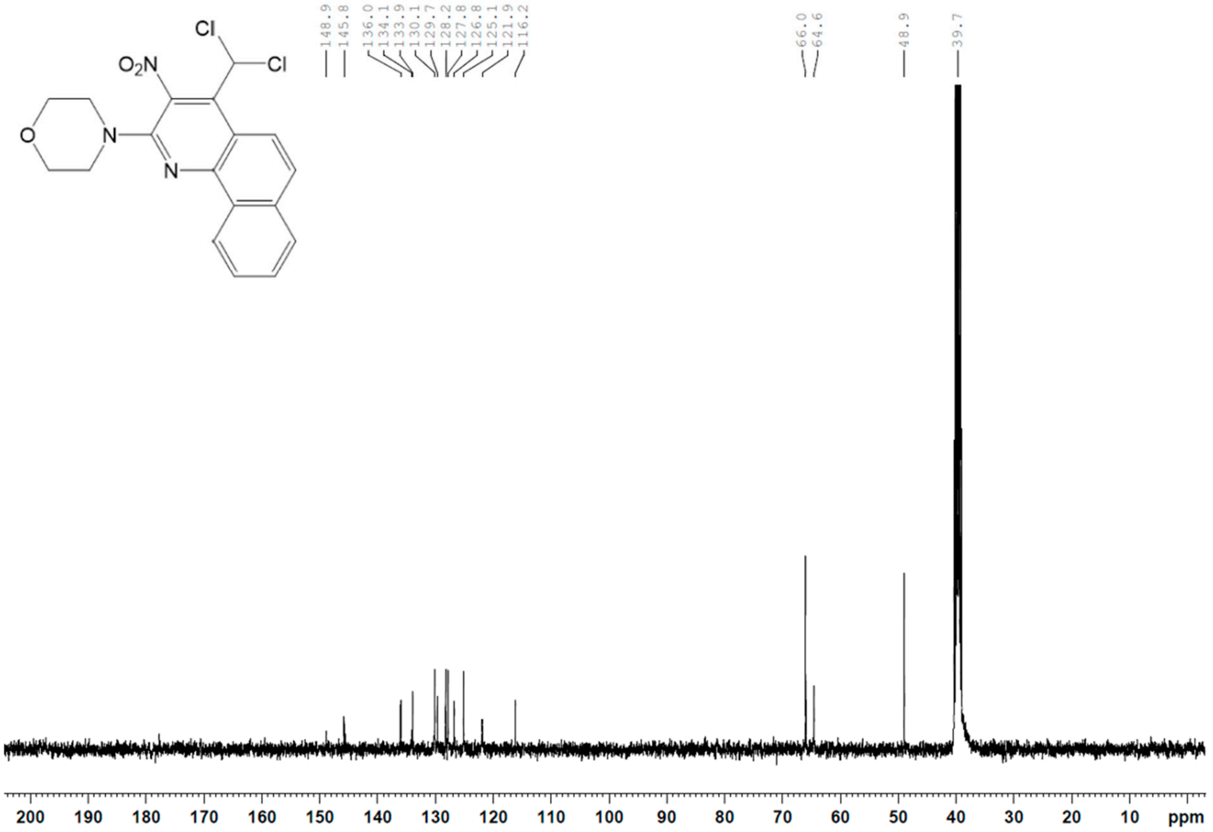

Figure S17. ^{1}H and ^{13}C NMR spectra of compound **12**

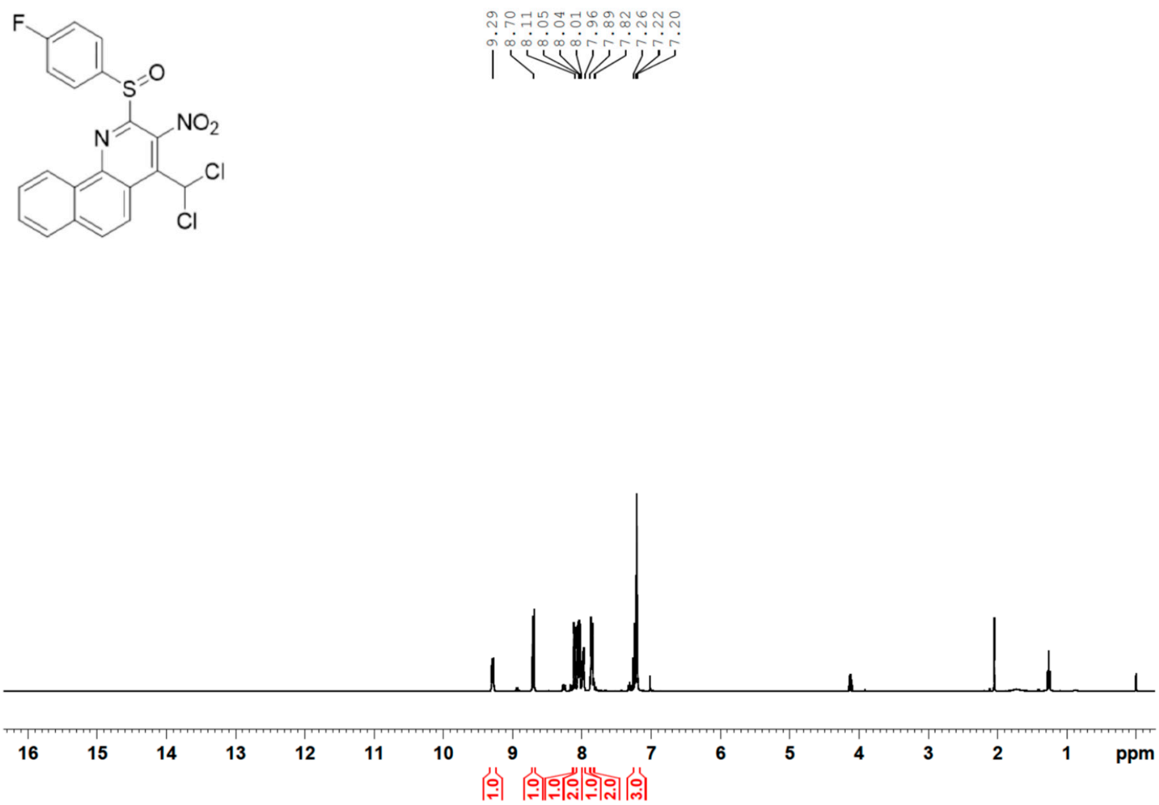
F
S=O
NO2
N
Cl
Cl
9.29 8.70 8.11 8.05 8.04 8.01 7.96 7.89 7.82 7.26 7.22 7.20
16 15 14 13 12 11 10 9 8 7 6 5 4 3 2 1 ppm
1.0 1.0 1.0 2.0 1.0 2.0 3.0

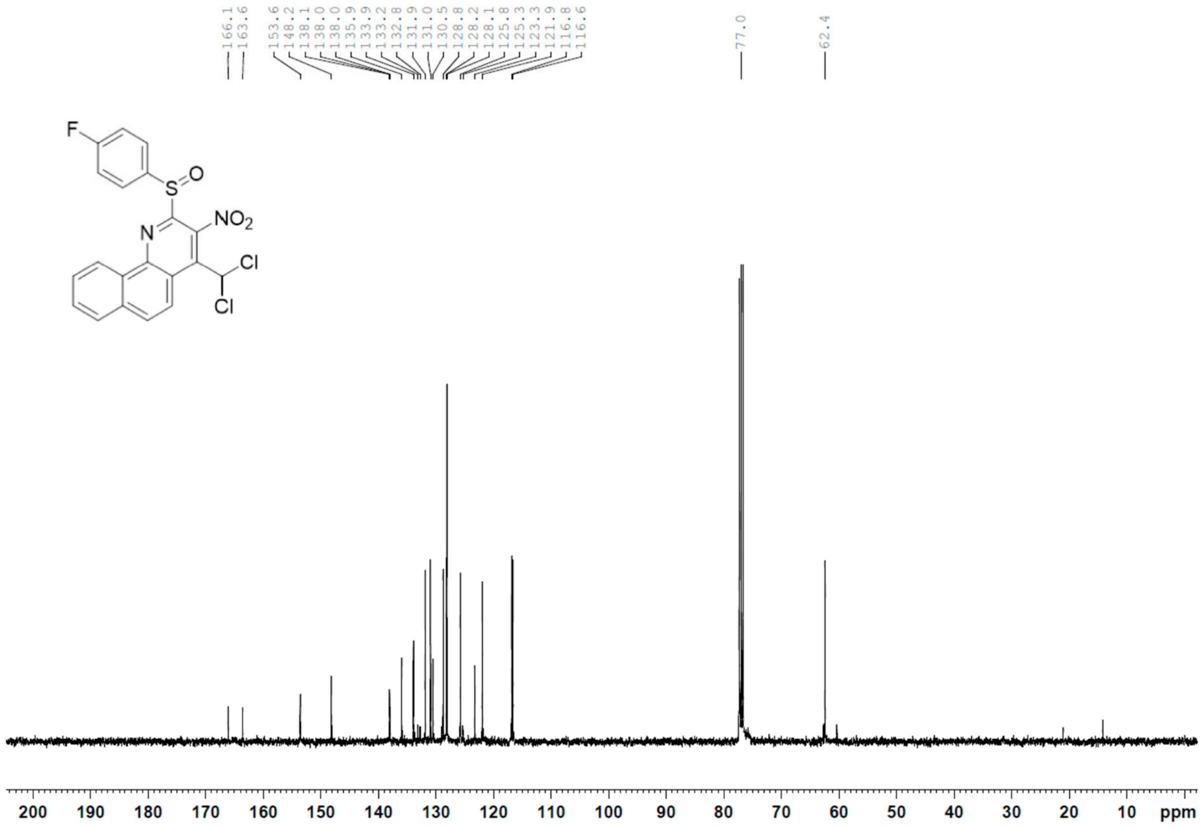
F
S=O
NO2
N
Cl
Cl
166.1 163.6 153.6 148.2 138.1 138.0 135.9 133.9 133.2 132.8 131.9 131.0 130.5 128.8 128.2 128.1 125.8 125.3 123.3 121.9 116.8 116.6 77.0 62.4
200 190 180 170 160 150 140 130 120 110 100 90 80 70 60 50 40 30 20 10 ppm

Figure S18. ^{1}H and ^{13}C NMR spectra of compound **13**

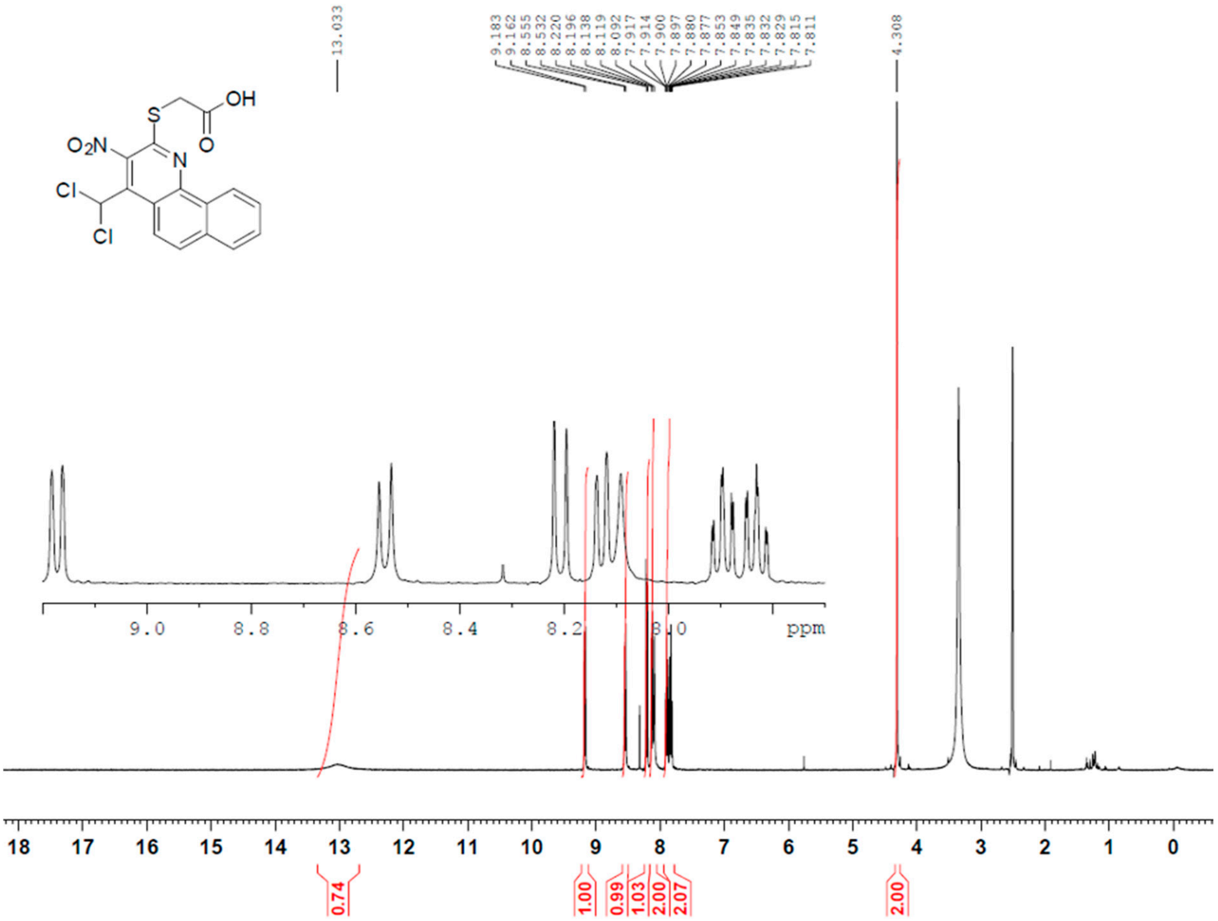

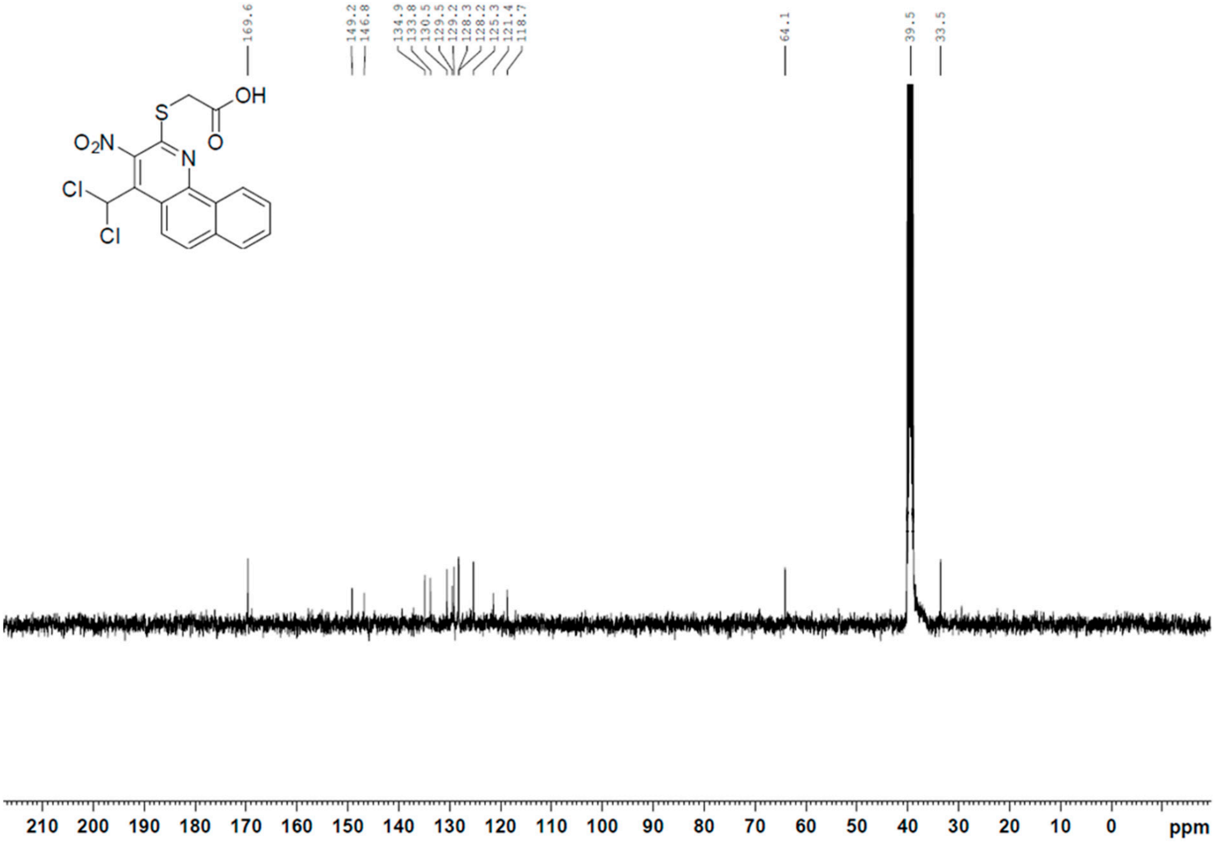

Figure S19. ^{1}H and ^{13}C NMR spectra of compound **14**

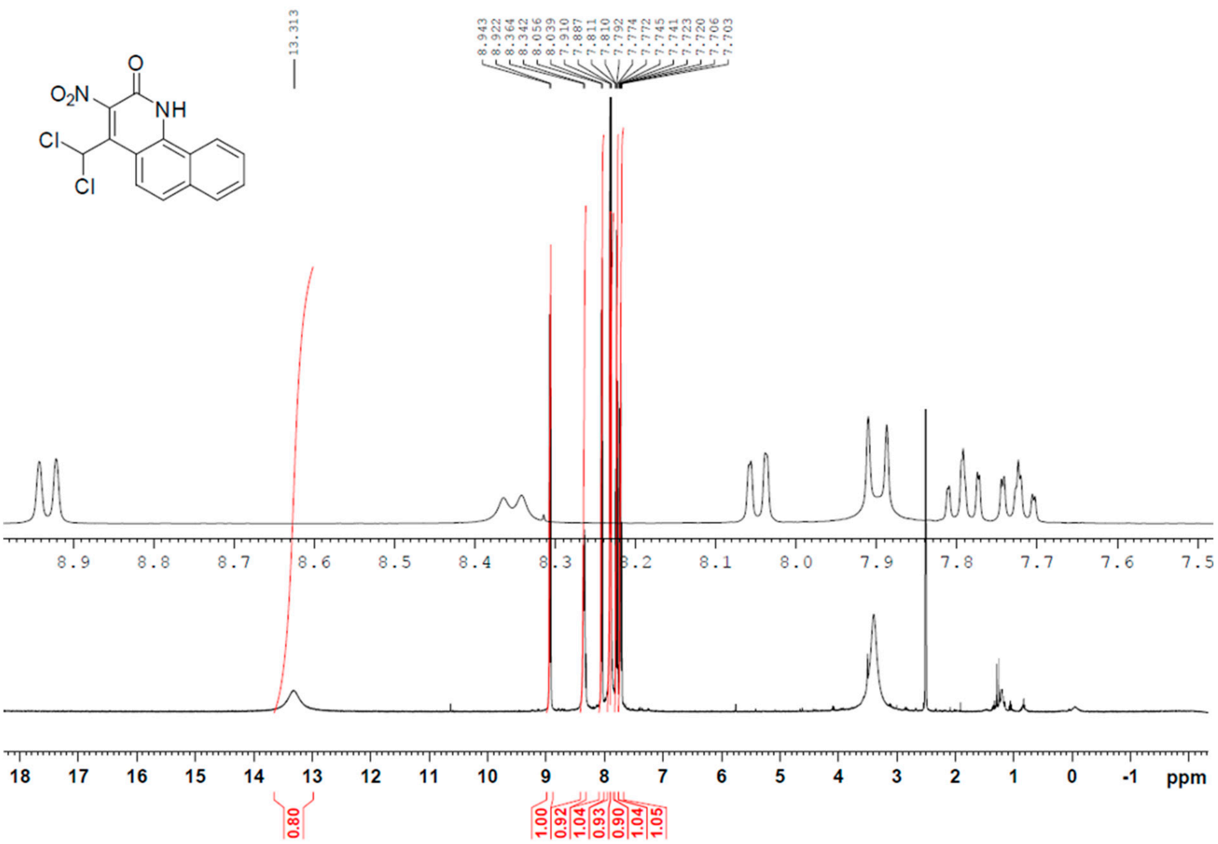

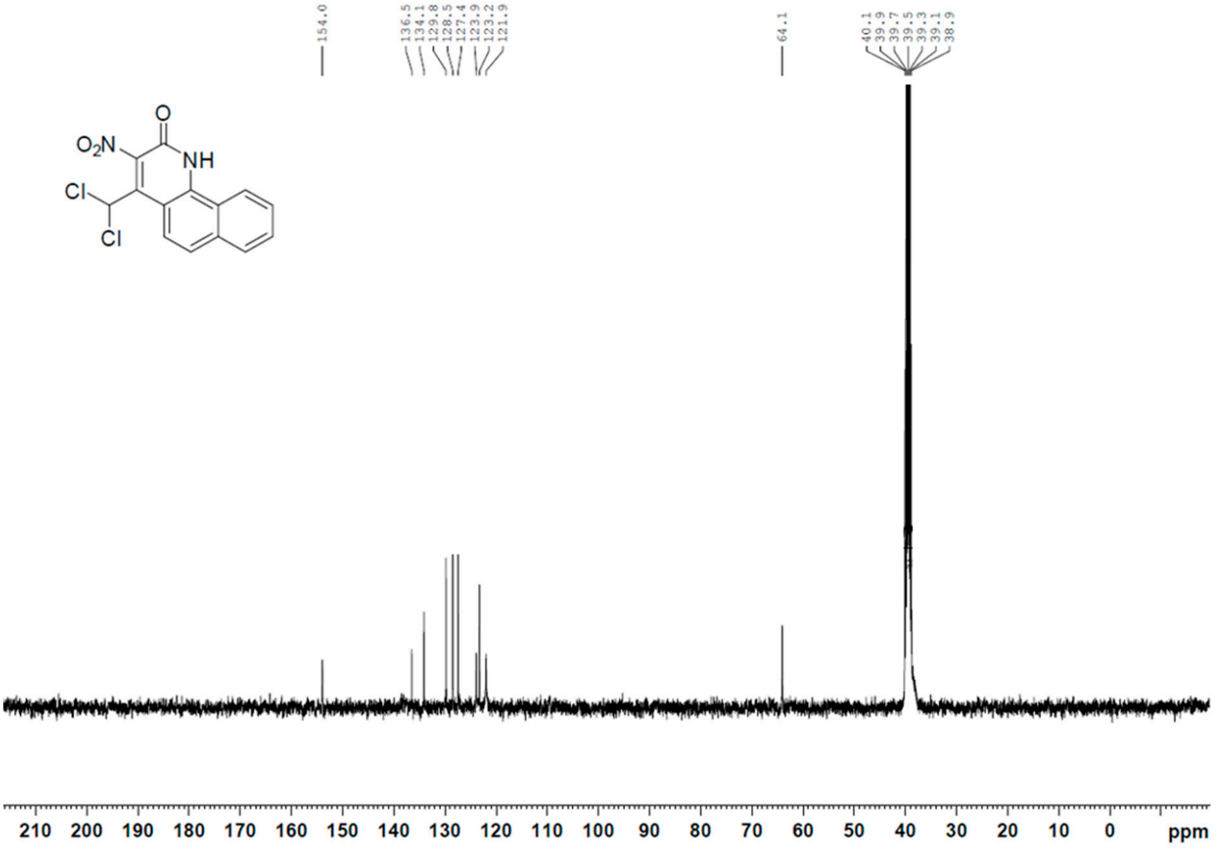

Figure S20. ^{1}H and ^{13}C NMR spectra of compound **15a**

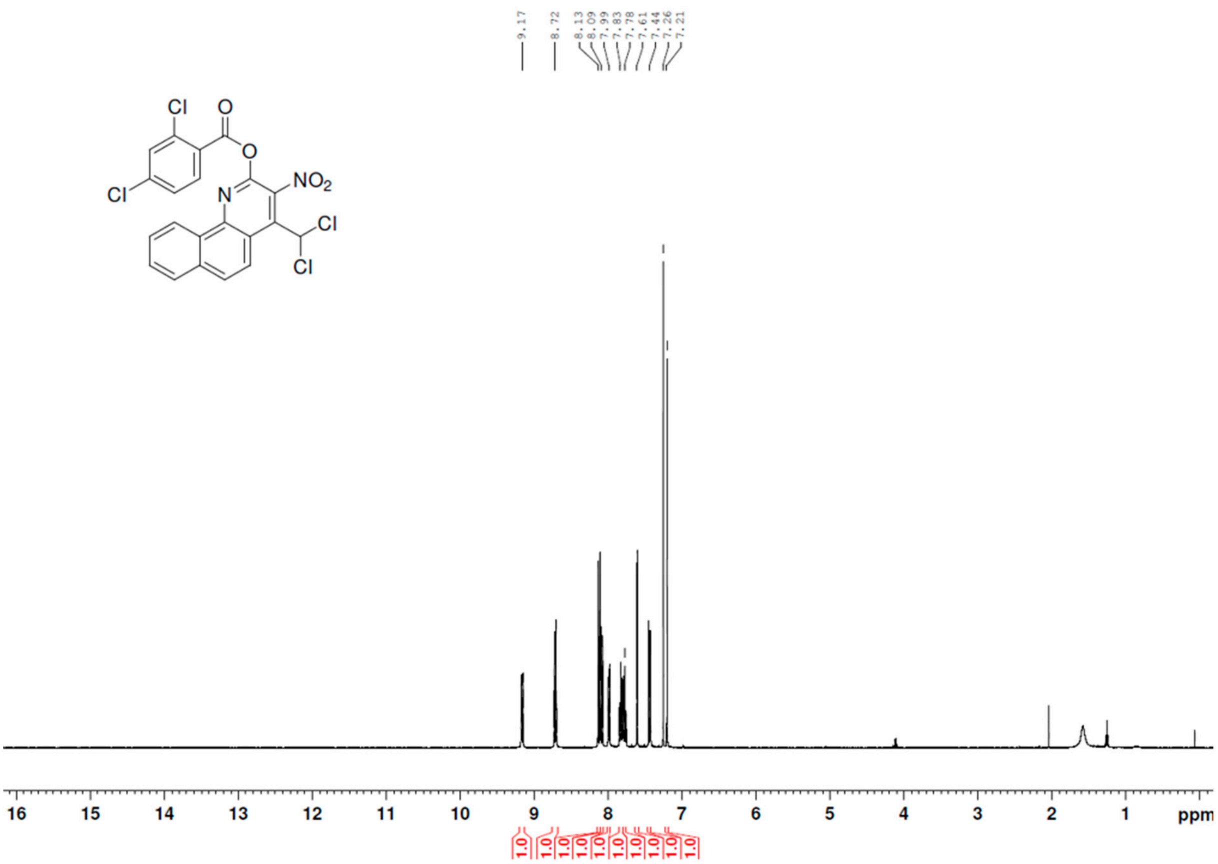

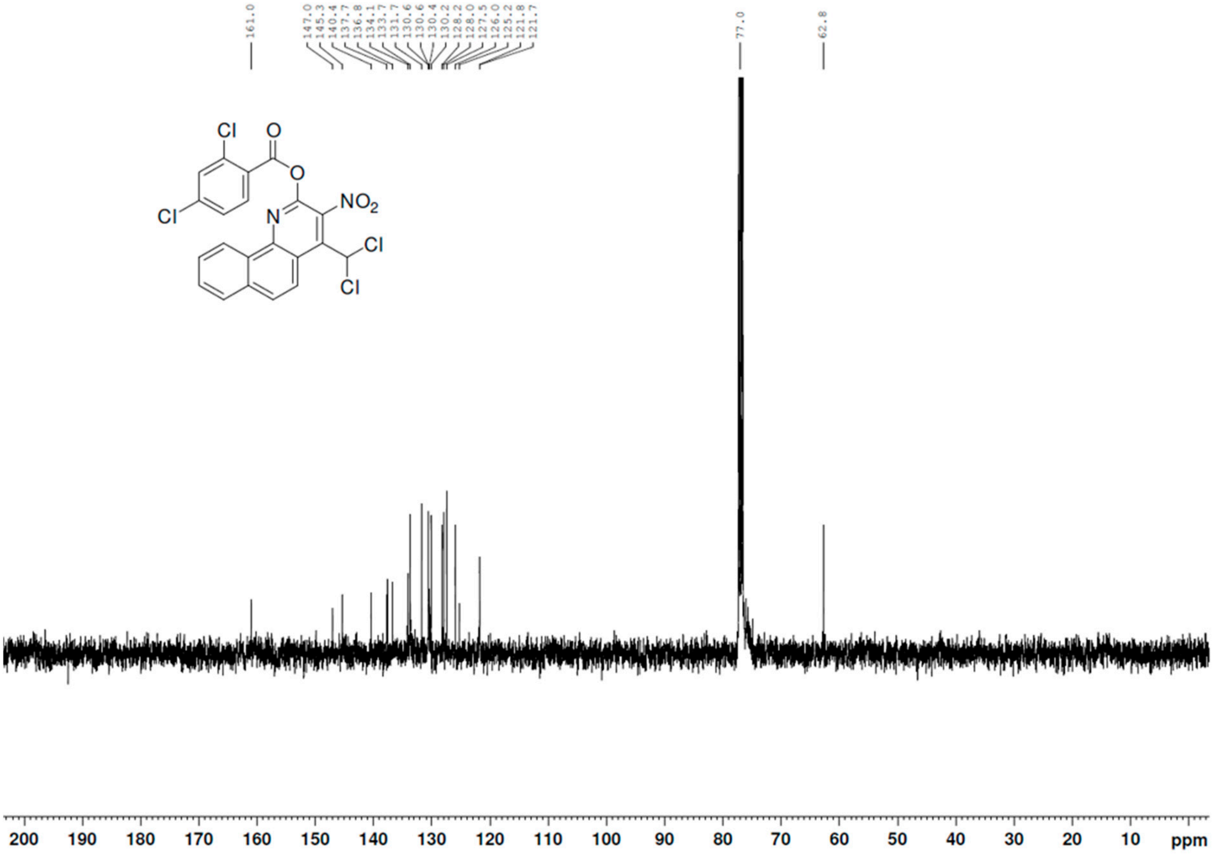

Figure S21. ^{1}H and ^{13}C NMR spectra of compound **15b**

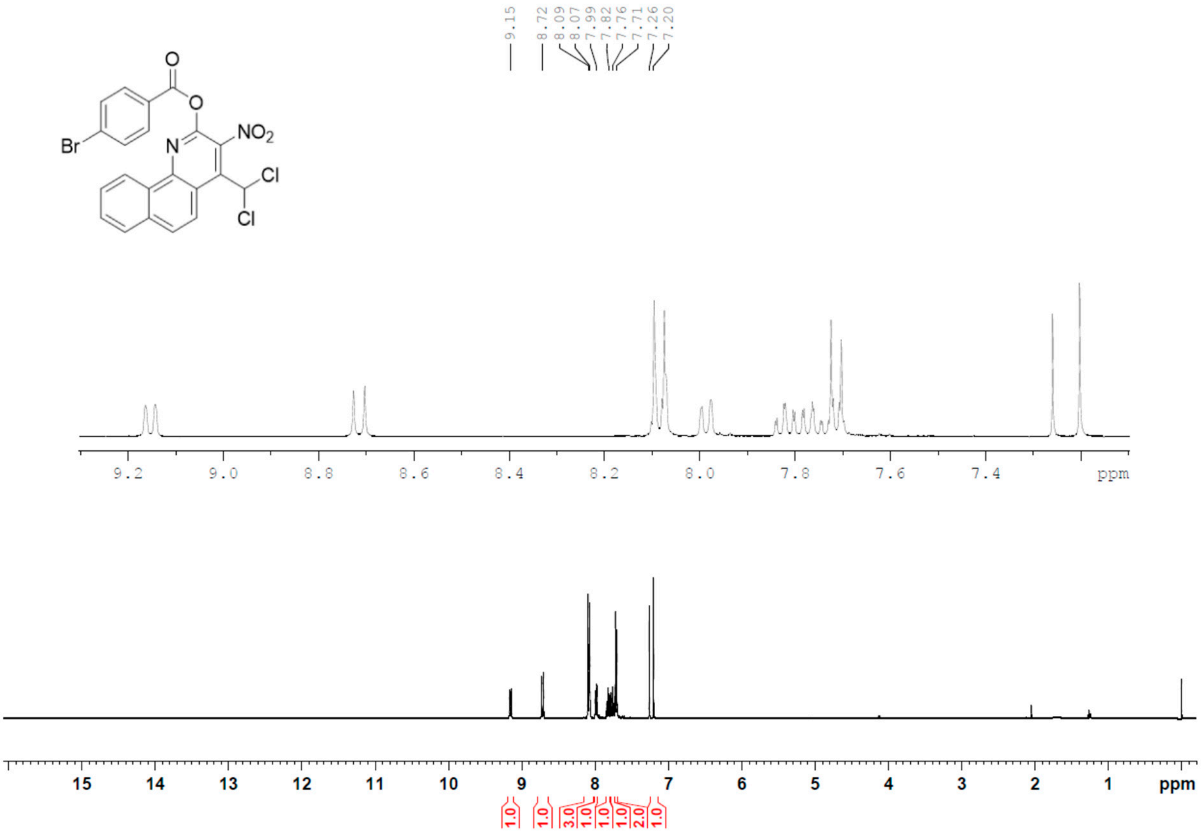
Br
O
NO2
N
Cl
Cl
9.15
8.72
8.09
8.07
7.99
7.82
7.76
7.71
7.26
7.20
9.2
9.0
8.8
8.6
8.4
8.2
8.0
7.8
7.6
7.4
ppm
15
14
13
12
11
10
9
8
7
6
5
4
3
2
1
ppm
1.0
1.0
3.0
1.0
1.0
1.0
2.0
1.0
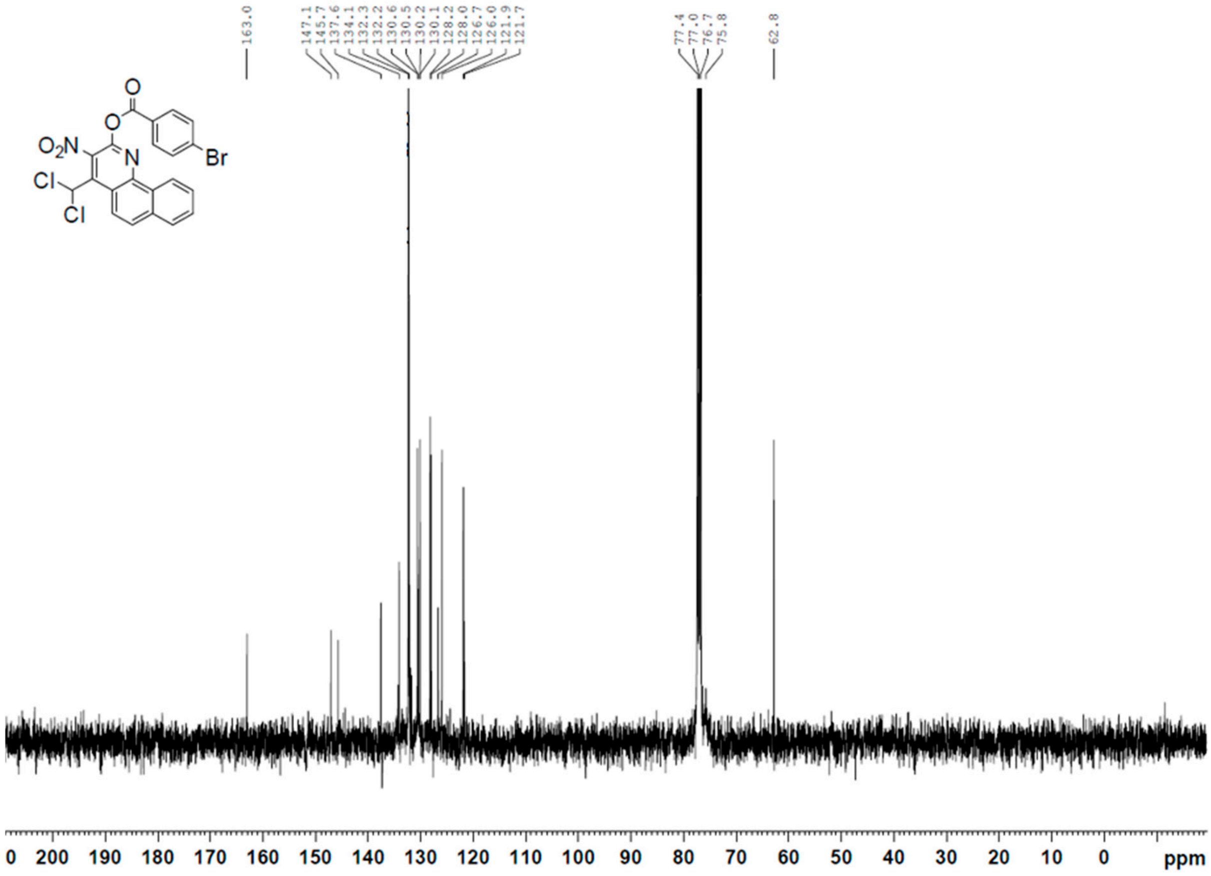
O
O2N
N
Br
Cl
Cl
163.0
147.1
145.7
137.6
134.1
132.3
132.2
130.6
130.5
130.2
130.1
128.2
128.0
126.7
126.0
121.9
121.7
77.4
77.0
76.7
75.8
62.8
0
200
190
180
170
160
150
140
130
120
110
100
90
80
70
60
50
40
30
20
10
0
ppm

Figure S22. ^{1}H and ^{13}C NMR spectra of compound **16c**

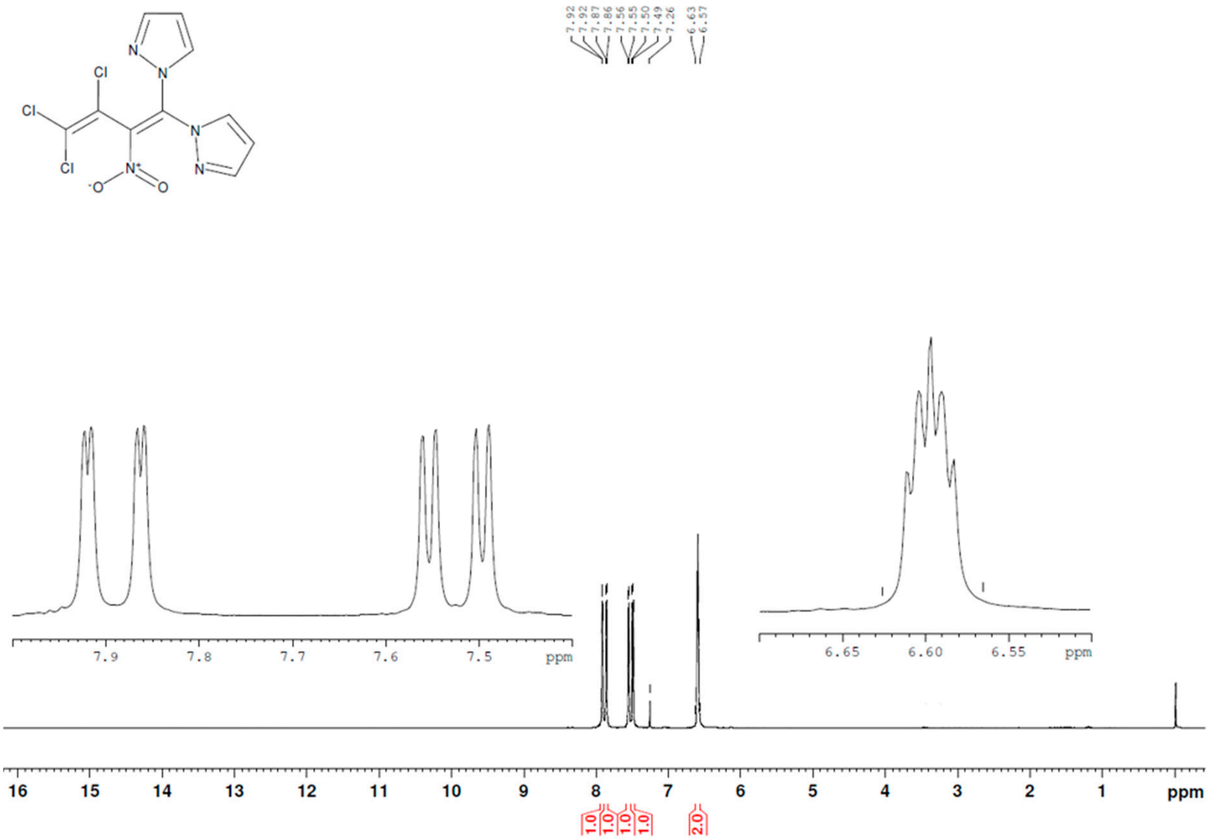

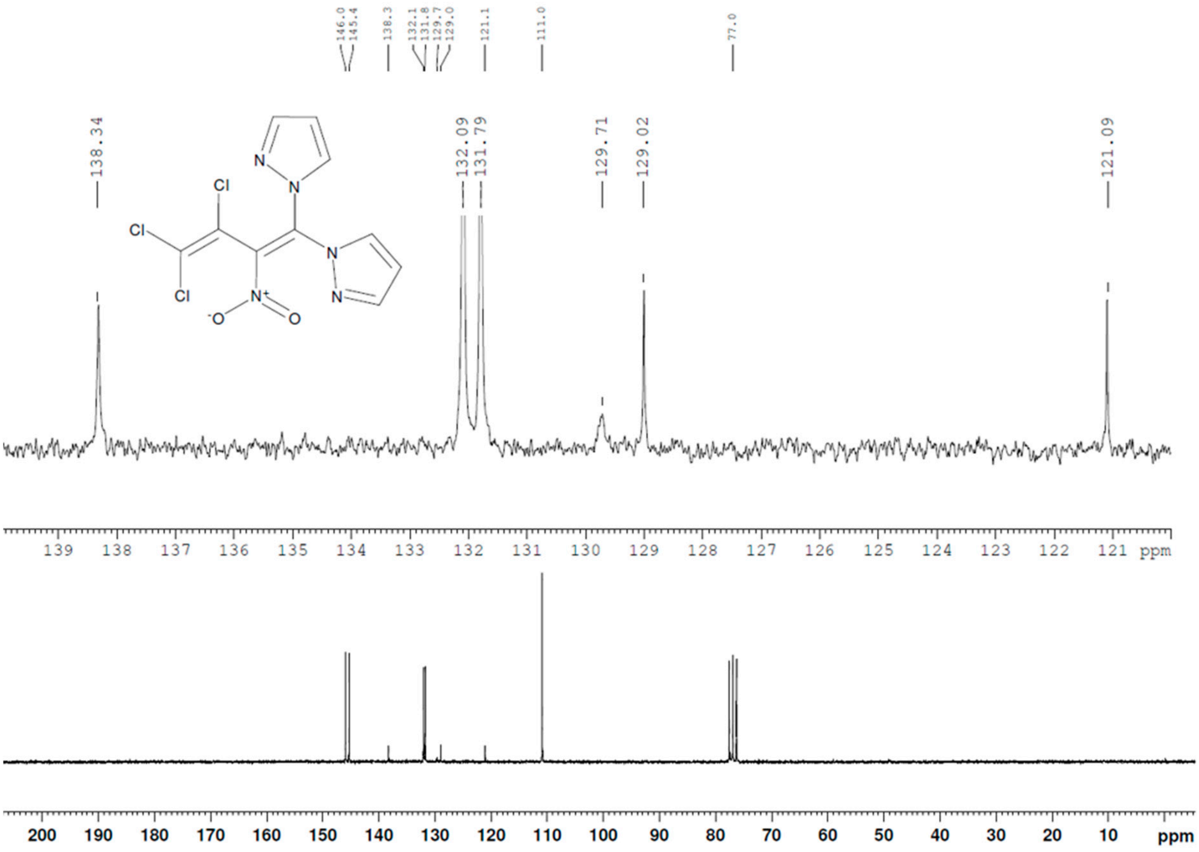

Figure S23. ^{1}H and ^{13}C NMR spectra of compound **17b**

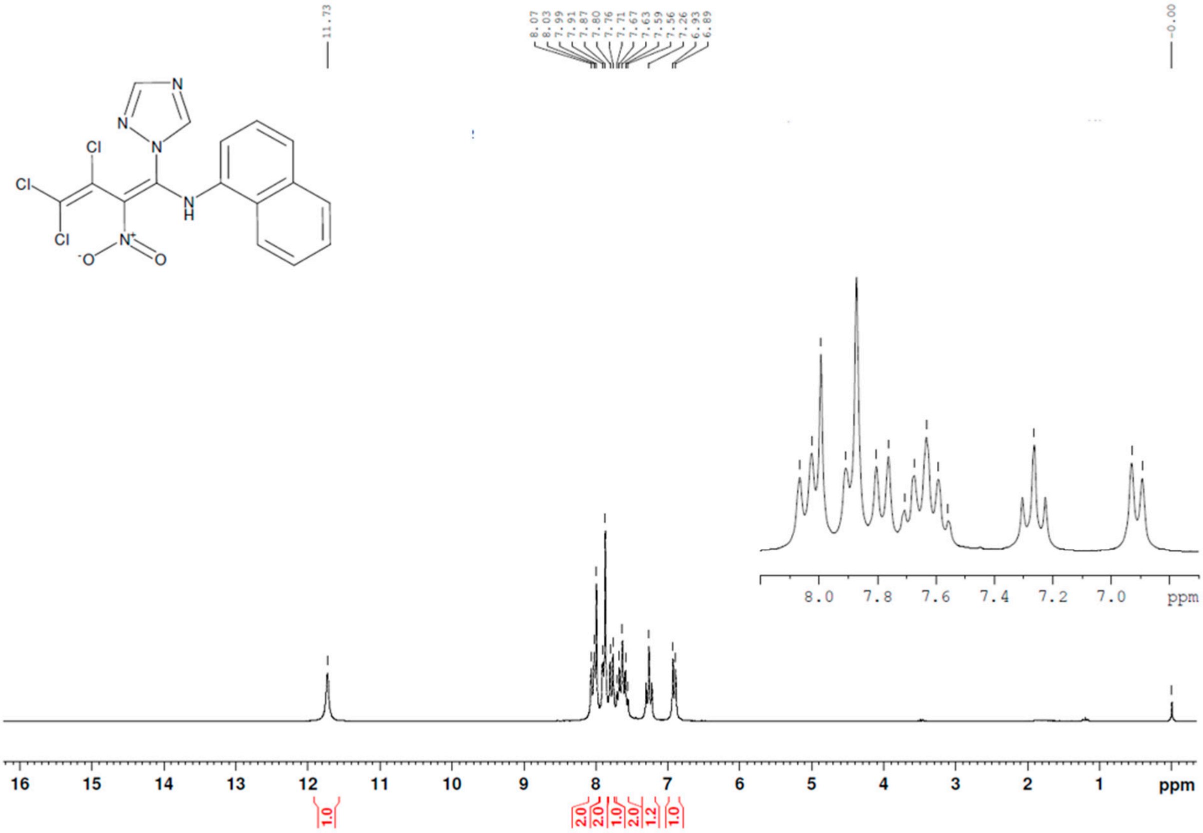

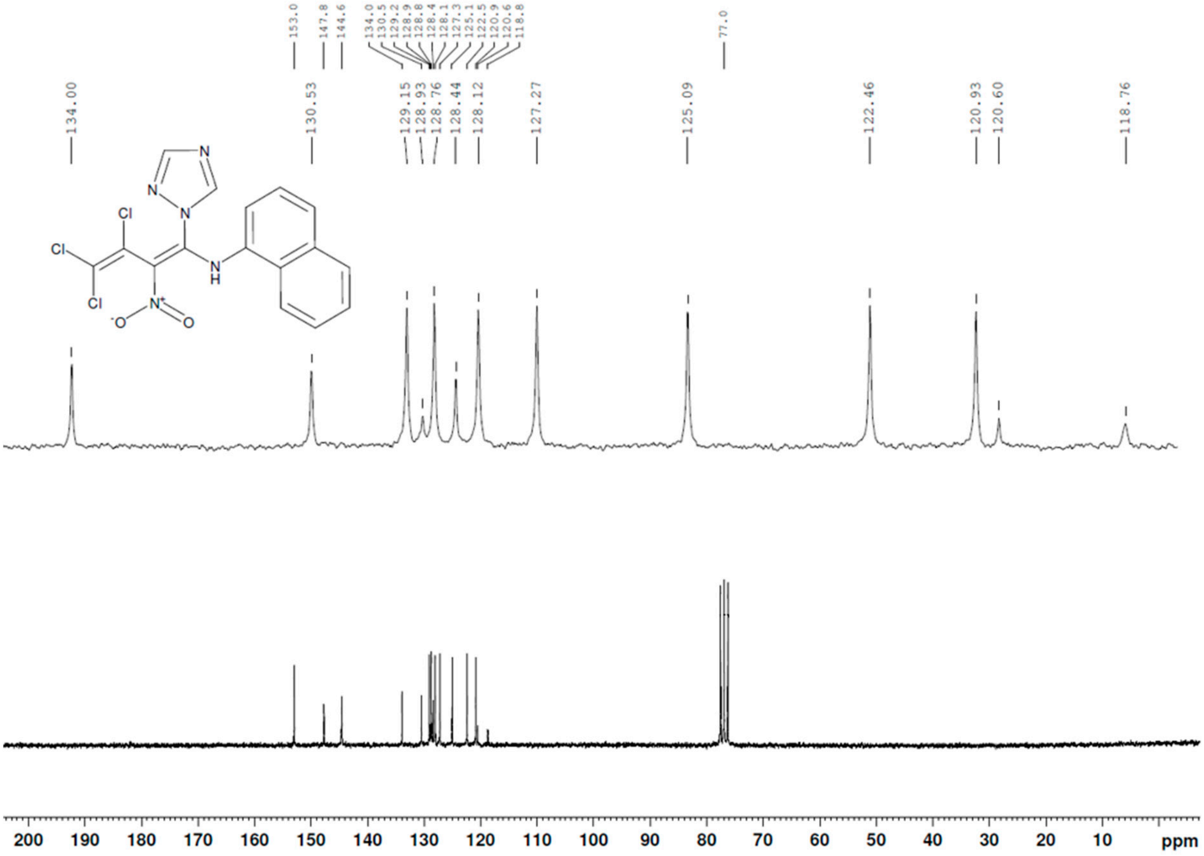

Figure S24. ^{1}H and ^{13}C NMR spectra of compound **17c**

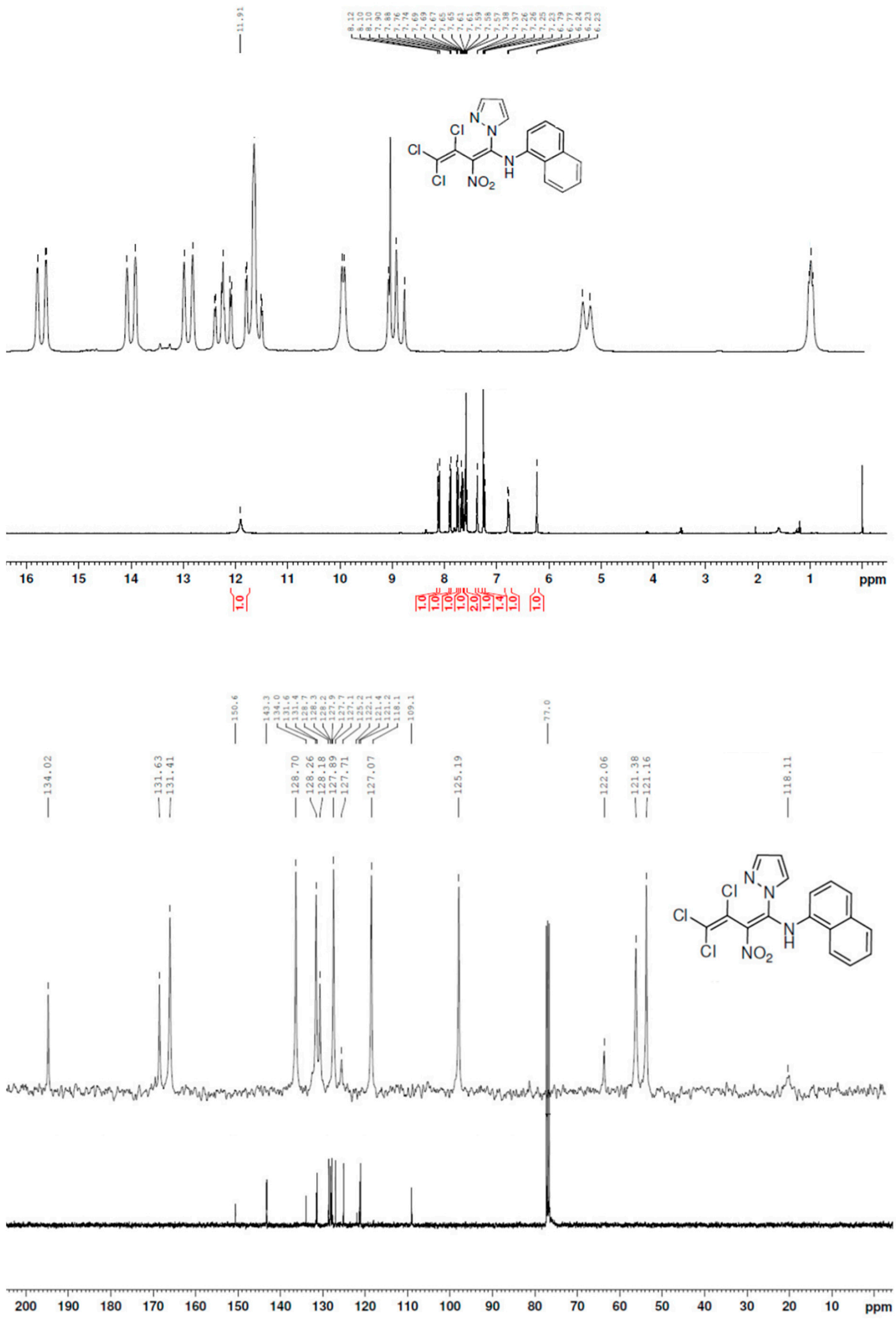

Figure S25. ^{1}H and ^{13}C NMR spectra of compound **18a**

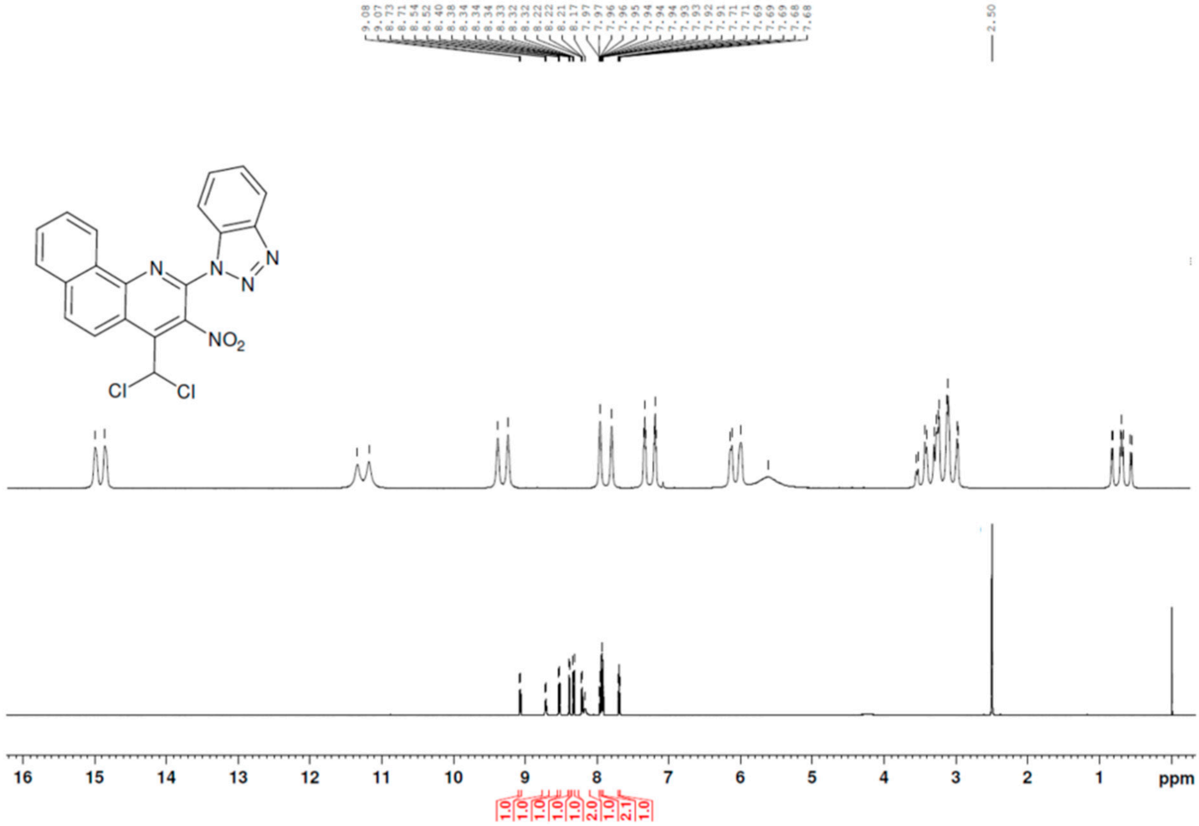

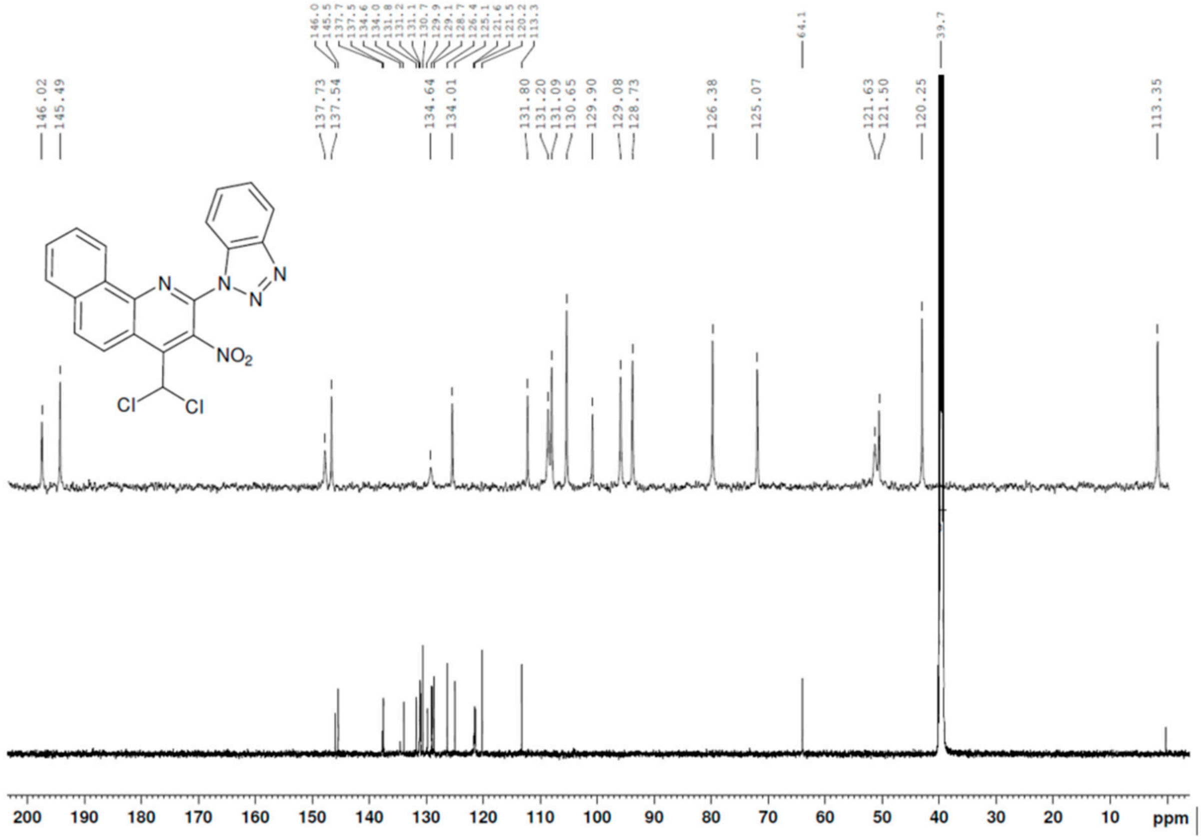

Figure S26. ^{1}H and ^{13}C NMR spectra of compound **18b**

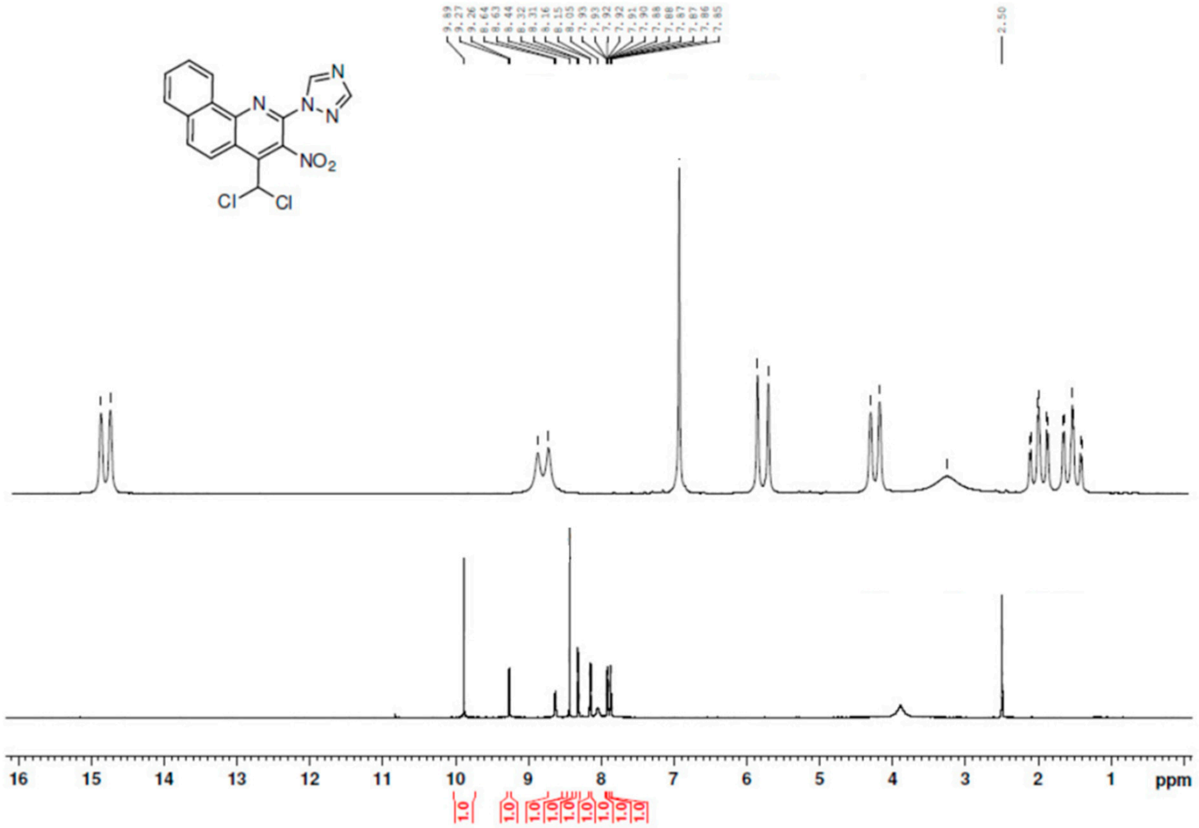

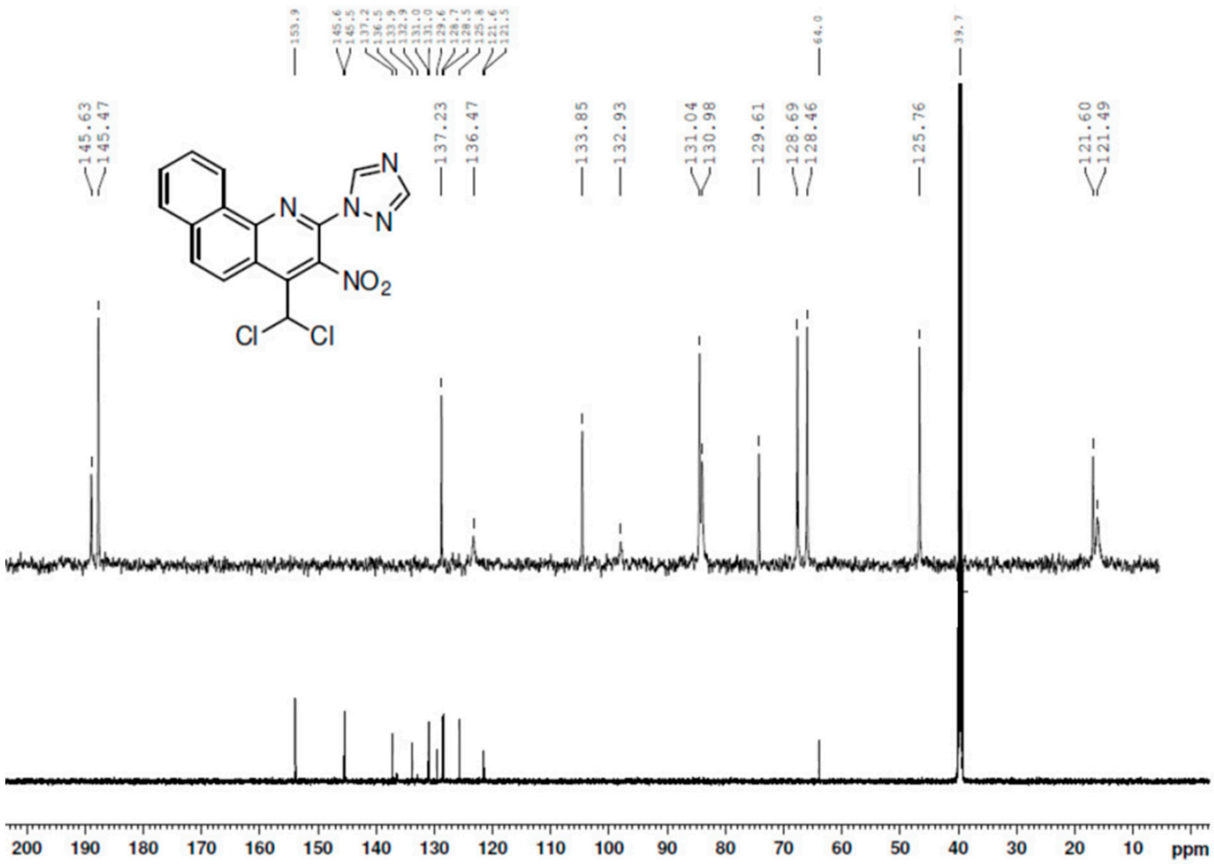

Figure S27. ^{1}H and ^{13}C NMR spectra of compound **18c**

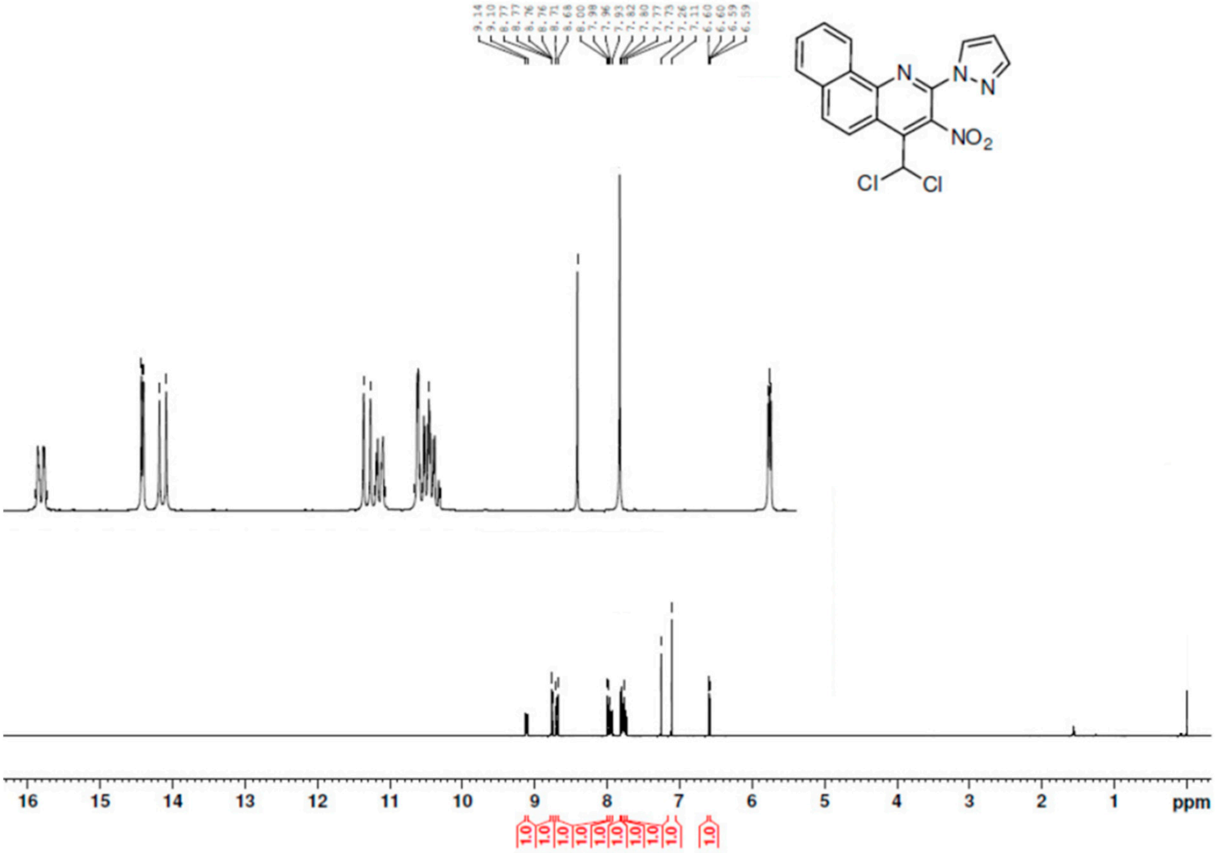

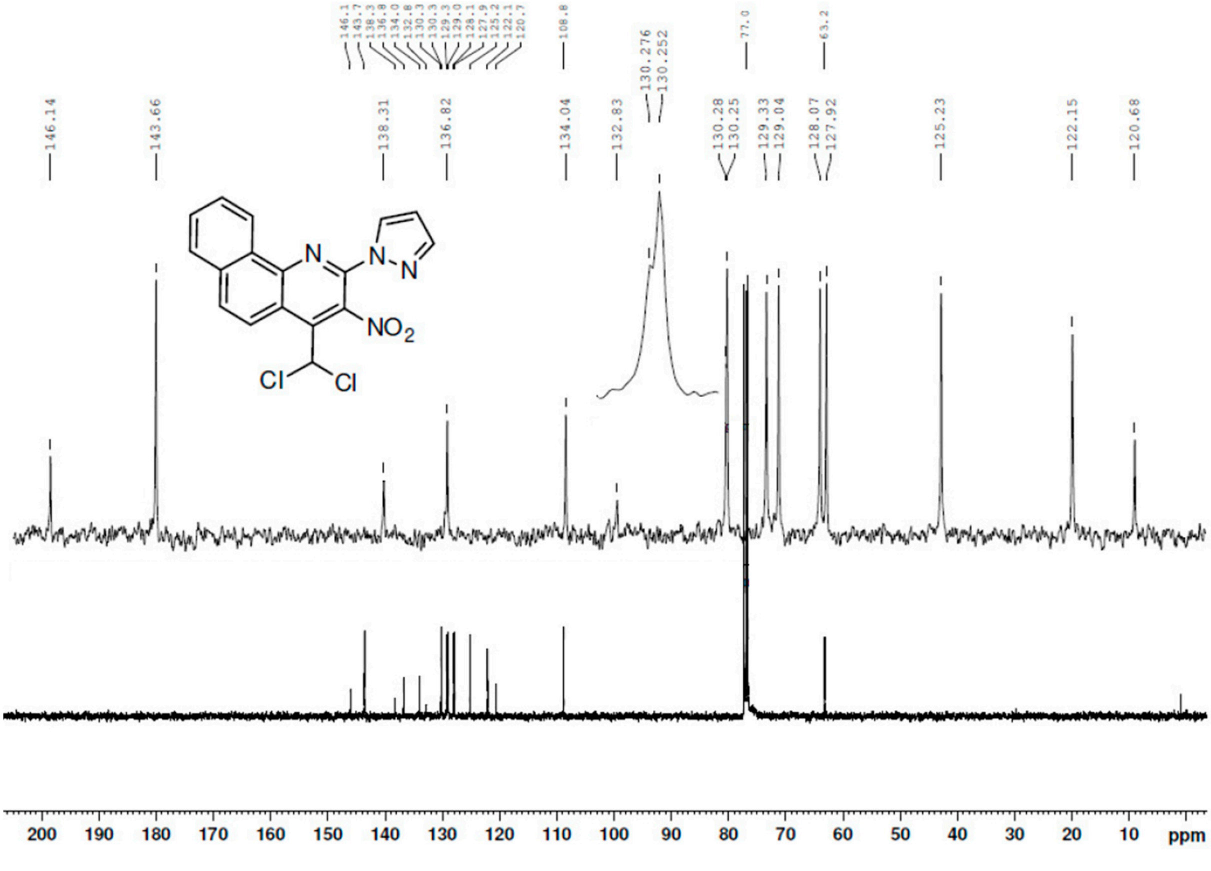

Figure S28. HRMS spectrum of **4a**

Elemental Composition Report

4a

Single Mass Analysis

Tolerance = 20.0 PPM / DBE: min = -1.5, max = 50.0
Element prediction: Off
Number of isotope peaks used for i-FIT = 3

Monoisotopic Mass, Odd and Even Electron Ions
532 formula(e) evaluated with 19 results within limits (up to 50 closest results for each mass)
Elements Used:
C: 0-20 H: 0-18 N: 0-3 O: 0-8 Na: 0-1 S: 0-1 Cl: 0-3
Kaul LCT 04-May-2012
SK16 54 (0.612) AM (Cen,5, 70.00, Ar,5000.0,490.89,1.00,LS 3) 1: TOF MS ES+
2.10e+002

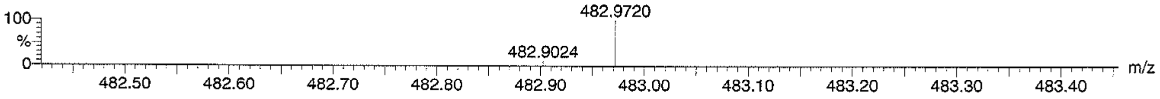

Mass	Calc. Mass	mDa	PPM	DBE	i-FIT	Formula
Minimum:				-1.5		
Maximum:		6.0	20.0	50.0		
482.9720	482.9716	0.4	0.8	10.5	n/a	C18 H15 N2 O4 Na S Cl3
	482.9713	0.7	1.4	9.0	n/a	C17 H16 N O7 S Cl3
	482.9708	1.2	2.5	13.5	n/a	C20 H13 O8 S Cl2
	482.9740	-2.0	-4.1	13.5	n/a	C20 H14 N2 O4 S Cl3
	482.9695	2.5	5.2	14.0	n/a	C18 H11 N3 O7 S Cl2
	482.9689	3.1	6.4	6.0	n/a	C15 H17 N O7 Na S Cl3
	482.9684	3.6	7.5	10.5	n/a	C18 H14 O8 Na S Cl2
	482.9679	4.1	8.5	14.0	n/a	C20 H12 N O7 Cl3
	482.9763	-4.3	-8.9	15.5	n/a	C20 H10 N2 O7 Na Cl2
	482.9768	-4.8	-9.9	11.0	n/a	C17 H13 N3 O6 Na Cl3
	482.9671	4.9	10.1	11.0	n/a	C16 H12 N3 O7 Na S Cl2
	482.9666	5.4	11.2	15.5	n/a	C19 H9 N2 O8 Na S Cl
	482.9781	-6.1	-12.6	10.5	n/a	C19 H15 O7 Na Cl3
	482.9655	6.5	13.5	11.0	n/a	C18 H13 N O7 Na Cl3
	482.9792	-7.2	-14.9	15.0	n/a	C20 H11 N O8 Na S Cl
	482.9792	-7.2	-14.9	14.0	n/a	C19 H12 N3 O6 Cl3
	482.9796	-7.6	-15.7	10.5	n/a	C17 H14 N2 O7 Na S Cl2
	482.9801	-8.1	-16.8	6.0	n/a	C14 H17 N3 O6 Na S Cl3
	482.9637	8.3	17.2	16.0	n/a	C19 H8 N3 O7 Na Cl2

Figure S29. HRMS spectrum of **4b**

4 [illegible]

Elemental Composition Report

Single Mass Analysis (displaying only valid results)

Tolerance = 7.0 PPM / DBE: min = -1.5, max = 50.0
Selected filters: None

Monoisotopic Mass, Even Electron Ions
4996 formula(e) evaluated with 30 results within limits (up to 80 closest results for each mass)
Elements Used:
C: 0-50 H: 0-60 N: 0-5 O: 0-7 Na: 0-1 S: 0-2 Cl: 0-3
Kaul LCT Premier KD070
SK 381 18 (0.404) AM (Cen,4, 95.00, Ar,11000.0,556.28,0.70,LS 5) 1: TOF MS ES+ 1.39e3

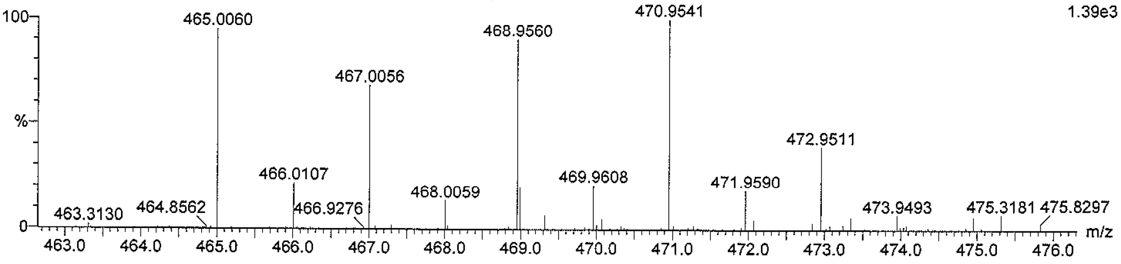

Mass	Calc. Mass	mDa	PPM	DBE	i-FIT	Formula
Minimum:				-1.5		
Maximum:		5.0	7.0	50.0		
468.9560	468.9559	0.1	0.2	10.5	2.2	C17 H13 N2 O4 Na S Cl3
	468.9563	-0.3	-0.6	24.5	274.6	C24 H3 N4 O2 Na S Cl
	468.9555	0.5	1.1	27.5	591.4	C26 H N2 O6 S
	468.9565	-0.5	-1.1	18.5	66.5	C20 H7 N4 O4 S Cl2
	468.9565	-0.5	-1.1	19.5	539.1	C21 H6 N2 O6 Na S2
	468.9566	-0.6	-1.3	19.5	13.3	C25 H9 O2 Na Cl3
	468.9553	0.7	1.5	1.5	22.6	C9 H17 N4 O6 Na S2 Cl3
	468.9550	1.0	2.1	18.5	7.3	C22 H8 N2 O4 Cl3
	468.9550	1.0	2.1	19.5	260.6	C23 H7 O6 Na S Cl
	468.9549	1.1	2.3	26.5	263.0	C29 H6 O S2 Cl
	468.9548	1.2	2.6	24.5	103.8	C26 H4 N2 O2 Na Cl2
	468.9572	-1.2	-2.6	28.5	598.6	C29 H2 O4 Na S
	468.9572	-1.2	-2.6	27.5	114.4	C28 H3 N2 O2 Cl2
	468.9574	-1.4	-3.0	22.5	267.5	C25 H6 O6 S Cl
	468.9575	-1.5	-3.2	10.5	46.9	C15 H12 N4 O4 Na S2 Cl2
	468.9543	1.7	3.6	9.5	6.1	C14 H12 N4 O6 S Cl3
	468.9577	-1.7	-3.6	4.5	12.9	C11 H16 N4 O6 S2 Cl3
	468.9541	1.9	4.1	15.5	63.0	C18 H8 N4 O4 Na S Cl2
	468.9540	2.0	4.3	27.5	314.1	C28 H2 O6 Cl
	468.9581	-2.1	-4.5	19.5	75.5	C23 H8 N2 O2 Na S Cl2
	468.9583	-2.3	-4.9	14.5	219.7	C20 H11 O6 Na S2 Cl
	468.9583	-2.3	-4.9	13.5	1.8	C19 H12 N2 O4 S Cl3
	468.9587	-2.7	-5.8	27.5	283.4	C26 H2 N4 O2 S Cl
	468.9588	-2.8	-6.0	28.5	130.7	C31 H4 Na Cl2
	468.9588	-2.8	-6.0	20.5	292.5	C20 H3 N4 O7 Na Cl
	468.9531	2.9	6.2	24.5	590.7	C24 H2 N2 O6 Na S
	468.9531	2.9	6.2	23.5	92.1	C23 H3 N4 O4 Cl2
	468.9589	-2.9	-6.2	22.5	539.7	C23 H5 N2 O6 S2
	468.9530	3.0	6.4	31.5	561.5	C30 H N2 O S2
	468.9590	-3.0	-6.4	22.5	20.6	C27 H8 O2 Cl3

Figure S30. HRMS spectrum of **5a**

Elemental Composition Report 5a

Single Mass Analysis

Tolerance = 20.0 PPM / DBE: min = -1.5, max = 50.0
Element prediction: Off
Number of isotope peaks used for i-FIT = 3

Monoisotopic Mass, Odd and Even Electron Ions
247 formula(e) evaluated with 8 results within limits (up to 50 closest results for each mass)
Elements Used:
C: 0-22 H: 0-20 N: 0-3 O: 0-5 Na: 0-1 S: 0-1 Cl: 0-2

Kaul LCT 07-May-2012
SK_9 39 (0.442) AM (Cen,5, 50.00, Ar,5000.0,490.89,1.00,LS 5) 1: TOF MS ES+
6.74e+002

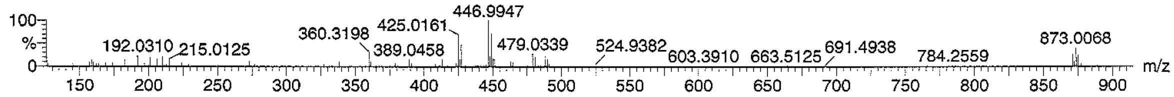

Mass	Calc. Mass	mDa	PPM	DBE	i-FIT	Formula
Minimum:				-1.5		
Maximum:		6.0	20.0	50.0		
446.9947	446.9949	-0.2	-0.4	11.5	0.1	C18 H14 N2 O4 Na S Cl2
	446.9944	0.3	0.7	16.0	45.8	C21 H11 N O5 Na S Cl
	446.9926	2.1	4.7	21.0	193.1	C22 H6 N3 O5 Na S
	446.9973	-2.6	-5.8	14.5	1.1	C20 H13 N2 O4 S Cl2
	446.9976	-2.9	-6.5	16.0	2.4	C21 H12 N3 O Na S Cl2
	446.9915	3.2	7.2	16.5	2.7	C21 H10 N2 O4 Na Cl2
	446.9864	8.3	18.6	16.0	4.0	C22 H12 N O2 Na S Cl2
	446.9861	8.6	19.2	14.5	2.7	C21 H13 O5 S Cl2

Figure S31. HRMS spectrum of **5b**

56

Elemental Composition Report

Single Mass Analysis (displaying only valid results)
Tolerance = 7.0 PPM / DBE: min = -1.5, max = 50.0
Selected filters: None

Monoisotopic Mass, Even Electron Ions
4567 formula(e) evaluated with 32 results within limits (up to 80 closest results for each mass)
Elements Used:
C: 0-50 H: 0-60 N: 0-5 O: 0-7 Na: 0-1 S: 0-2 Cl: 0-3
Kaul LCT Premier KD070
SK 380 33 (0.740) AM (Cen,4, 72.00, Ar,11000.0,556.28,0.70,LS 5) 1: TOF MS ES+ 215

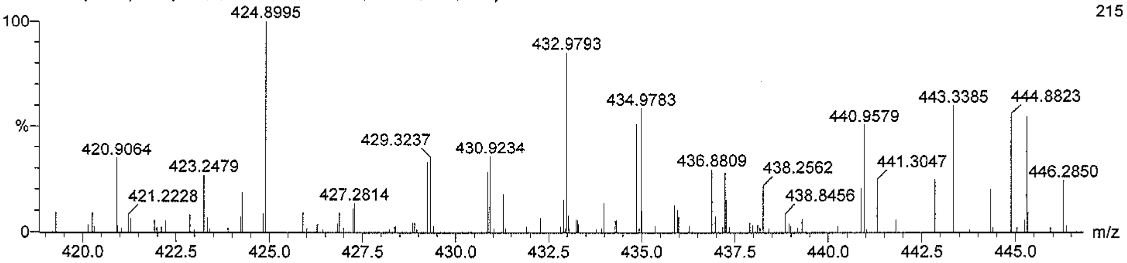

Minimum:				-1.5		
Maximum:		5.0	7.0	50.0		
Mass	Calc. Mass	mDa	PPM	DBE	i-FIT	Formula
432.9793	432.9793	0.0	0.0	11.5	1.3	C17 H12 N2 O4 Na S Cl2
	432.9795	-0.2	-0.5	5.5	6.3	C13 H16 N2 O6 S Cl3
	432.9796	-0.3	-0.7	25.5	56.5	C24 H2 N4 O2 Na S
	432.9790	0.3	0.7	16.5	5.7	C20 H9 N4 Na Cl3
	432.9798	-0.5	-1.2	19.5	15.9	C20 H6 N4 O4 S Cl
	432.9799	-0.6	-1.4	20.5	7.1	C25 H8 O2 Na Cl2
	432.9786	0.7	1.6	2.5	0.6	C9 H16 N4 O6 Na S2 Cl2
	432.9801	-0.8	-1.8	14.5	5.8	C21 H12 O4 Cl3
	432.9783	1.0	2.3	20.5	53.9	C23 H6 O6 Na S
	432.9783	1.0	2.3	19.5	4.8	C22 H7 N2 O4 Cl2
	432.9782	1.1	2.5	27.5	54.6	C29 H5 O S2
	432.9805	-1.2	-2.8	28.5	27.7	C28 H2 N2 O2 Cl
	432.9781	1.2	2.8	25.5	25.1	C26 H3 N2 O2 Na Cl
	432.9807	-1.4	-3.2	23.5	55.3	C25 H5 O6 S
	432.9808	-1.5	-3.5	11.5	9.1	C15 H11 N4 O4 Na S2 Cl
	432.9777	1.6	3.7	11.5	5.2	C19 H13 O4 Na Cl3
	432.9810	-1.7	-3.9	5.5	0.3	C11 H15 N4 O6 S2 Cl2
	432.9776	1.7	3.9	10.5	0.4	C14 H11 N4 O6 S Cl2
	432.9811	-1.8	-4.2	6.5	6.0	C16 H17 O4 Na S Cl3
	432.9774	1.9	4.4	16.5	14.0	C18 H7 N4 O4 Na S Cl
	432.9773	2.0	4.6	28.5	63.3	C28 H O6
	432.9814	-2.1	-4.9	20.5	18.8	C23 H7 N2 O2 Na S Cl
	432.9771	2.2	5.1	2.5	6.9	C11 H17 N2 O6 Na S Cl3
	432.9815	-2.2	-5.1	19.5	6.7	C22 H8 N4 Cl3
	432.9770	2.3	5.3	9.5	7.4	C17 H16 N2 O S2 Cl3
	432.9817	-2.4	-5.5	14.5	2.5	C19 H11 N2 O4 S Cl2
	432.9817	-2.4	-5.5	15.5	45.9	C20 H10 O6 Na S2
	432.9820	-2.7	-6.2	28.5	58.1	C26 H N4 O2 S
	432.9821	-2.8	-6.5	29.5	31.1	C31 H3 Na Cl
	432.9821	-2.8	-6.5	21.5	59.3	C20 H2 N4 O7 Na
	432.9765	2.8	6.5	24.5	22.0	C23 H2 N4 O4 Cl
	432.9823	-3.0	-6.9	23.5	9.4	C27 H7 O2 Cl2

Figure S32. HRMS spectrum of **6**

6

Elemental Composition Report

Single Mass Analysis (displaying only valid results)
Tolerance = 7.0 PPM / DBE: min = -1.5, max = 50.0
Selected filters: None

Monoisotopic Mass, Even Electron Ions
4097 formula(e) evaluated with 30 results within limits (up to 80 closest results for each mass)
Elements Used:
C: 0-50 H: 0-60 N: 0-5 O: 0-5 Na: 0-1 S: 0-2 Cl: 0-3

Zapolski LCT Premier KD070
VZ 2310 17 (0.387) AM (Cen,4, 70.00, Ar,11000.0,556.28,0.70,LS 5) 1: TOF MS ES+

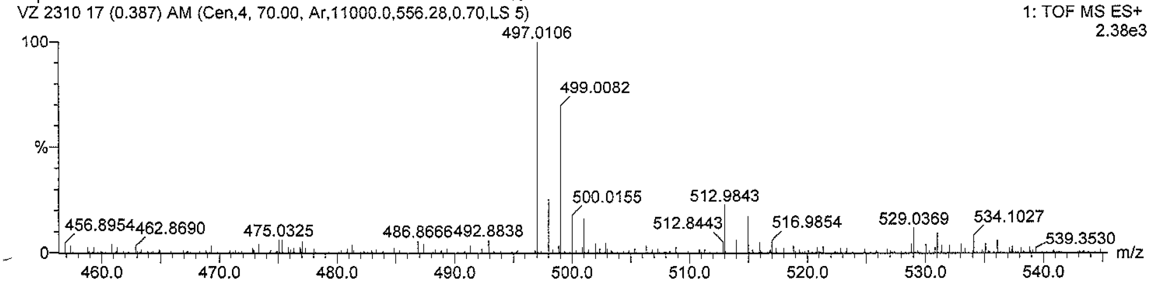

Mass	Calc. Mass	mDa	PPM	DBE	i-FIT	Formula
Minimum:				-1.5		
Maximum:		5.0	7.0	50.0		
497.0106	497.0106	0.0	0.0	14.5	0.9	C22 H16 N2 O4 Na S Cl2
	497.0103	0.3	0.6	19.5	66.3	C25 H13 N4 Na Cl3
	497.0109	-0.3	-0.6	28.5	632.9	C29 H6 N4 O2 Na S
	497.0111	-0.5	-1.0	22.5	135.3	C25 H10 N4 O4 S Cl
	497.0112	-0.6	-1.2	23.5	23.7	C30 H12 O2 Na Cl2
	497.0100	0.6	1.2	36.5	726.0	C34 H N4 O2
	497.0114	-0.8	-1.6	17.5	69.2	C26 H16 O4 Cl3
	497.0116	-1.0	-2.0	37.5	738.3	C37 H2 N2 Na
	497.0096	1.0	2.0	22.5	11.7	C27 H11 N2 O4 Cl2
	497.0095	1.1	2.2	30.5	565.1	C34 H9 O S2
	497.0094	1.2	2.4	28.5	210.1	C31 H7 N2 O2 Na Cl
	497.0118	-1.2	-2.4	31.5	225.1	C33 H6 N2 O2 Cl
	497.0121	-1.5	-3.0	14.5	85.9	C20 H15 N4 O4 Na S2 Cl
	497.0090	1.6	3.2	14.5	70.7	C24 H17 O4 Na Cl3
	497.0124	-1.8	-3.6	9.5	94.9	C21 H21 O4 Na S Cl3
	497.0087	1.9	3.8	19.5	128.0	C23 H11 N4 O4 Na S Cl
	497.0127	-2.1	-4.2	23.5	151.4	C28 H11 N2 O2 Na S Cl
	497.0128	-2.2	-4.4	22.5	68.1	C27 H12 N4 Cl3
	497.0083	2.3	4.6	12.5	105.7	C22 H20 N2 O S2 Cl3
	497.0130	-2.4	-4.8	17.5	3.8	C24 H15 N2 O4 S Cl2
	497.0133	-2.7	-5.4	31.5	636.5	C31 H5 N4 O2 S
	497.0134	-2.8	-5.6	32.5	249.2	C36 H7 Na Cl
	497.0078	2.8	5.6	27.5	191.4	C28 H6 N4 O4 Cl
	497.0136	-3.0	-6.0	26.5	37.0	C32 H11 O2 Cl2
	497.0137	-3.1	-6.2	14.5	86.1	C22 H17 N4 Na S Cl3
	497.0075	3.1	6.2	33.5	723.8	C32 H2 N4 O2 Na
	497.0073	3.3	6.6	21.5	113.9	C28 H14 O3 S2 Cl
	497.0139	-3.3	-6.6	9.5	7.7	C19 H20 N2 O4 Na S2 Cl2
	497.0072	3.4	6.8	19.5	5.3	C25 H12 N2 O4 Na Cl2
	497.0140	-3.4	-6.8	40.5	744.0	C39 H N2

Figure S33. HRMS spectrum of **7a**

7q

Elemental Composition Report

Single Mass Analysis (displaying only valid results)

Tolerance = 7.0 PPM / DBE: min = -1.5, max = 50.0
Selected filters: None

Monoisotopic Mass, Even Electron Ions
2145 formula(e) evaluated with 21 results within limits (up to 80 closest results for each mass)
Elements Used:
C: 0-50 H: 0-50 N: 0-2 O: 0-3 Na: 0-1 S: 0-2 Cl: 0-3 80Se: 0-1

Kaul LCT Premier KD070
SK 141 12 (0.263) AM (Cen,4, 70.00, Ar,11000.0,556.28,0.70,LS 5) 1: TOF MS ES+ 1.00e3

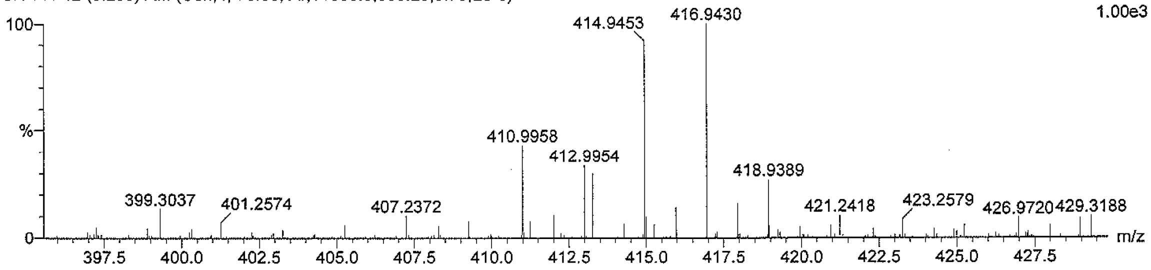

Minimum:				-1.5		
Maximum:		5.0	7.0	50.0		

Mass	Calc. Mass	mDa	PPM	DBE	i-FIT	Formula
414.9453	414.9454	-0.1	-0.2	12.5	276.7	C16 H12 N2 O Na S2 80Se
	414.9454	-0.1	-0.2	8.5	3.2	C14 H11 N2 O3 Na S Cl3
	414.9450	0.3	0.7	-1.5	38.1	C9 H22 O3 Na S2 Cl2 80Se
	414.9456	-0.3	-0.7	6.5	117.0	C12 H16 N2 O3 S2 Cl 80Se
	414.9460	-0.7	-1.7	17.5	31.0	C22 H7 O Na Cl3
	414.9444	0.9	2.2	16.5	19.2	C19 H6 N2 O3 Cl3
	414.9444	0.9	2.2	20.5	323.8	C21 H7 N2 O S 80Se
	414.9443	1.0	2.4	24.5	243.3	C26 H4 S2 Cl
	414.9463	-1.0	-2.4	15.5	165.4	C20 H12 O S Cl 80Se
	414.9442	1.1	2.7	22.5	110.7	C23 H2 N2 O Na Cl2
	414.9441	1.2	2.9	6.5	54.6	C14 H17 O3 S Cl2 80Se
	414.9466	-1.3	-3.1	25.5	126.2	C25 H N2 O Cl2
	414.9466	-1.3	-3.1	26.5	473.4	C26 O3 Na S
	414.9439	1.4	3.4	12.5	153.4	C18 H13 O Na S Cl 80Se
	414.9472	-1.9	-4.6	7.5	126.8	C15 H17 O Na S2 Cl 80Se
	414.9474	-2.1	-5.1	1.5	40.9	C11 H21 O3 S2 Cl2 80Se
	414.9432	2.1	5.1	3.5	113.2	C10 H17 N2 O3 Na S2 Cl 80Se
	414.9476	-2.3	-5.5	17.5	80.9	C20 H6 N2 O Na S Cl2
	414.9429	2.4	5.8	20.5	197.0	C23 H8 O Cl 80Se
	414.9478	-2.5	-6.0	15.5	285.7	C18 H11 N2 O S2 80Se
	414.9478	-2.5	-6.0	11.5	8.0	C16 H10 N2 O3 S Cl3

Figure S34. HRMS spectrum of **7b**

Elemental Composition Report

76

Single Mass Analysis (displaying only valid results)
Tolerance = 7.0 PPM / DBE: min = -1.5, max = 50.0
Selected filters: None

Monoisotopic Mass, Even Electron Ions
2907 formula(e) evaluated with 33 results within limits (up to 80 closest results for each mass)
Elements Used:
C: 0-50 H: 0-50 N: 0-2 O: 0-4 Na: 0-1 S: 0-2 Cl: 0-3 80Se: 0-1

Kaul
LCT Premier KD070
SK 295 10 (0.233) AM (Cen,4, 27.00, Ar,11000.0,554.26,0.70,LS 5); Cm (1:19)
1: TOF MS ES-
1.25e4

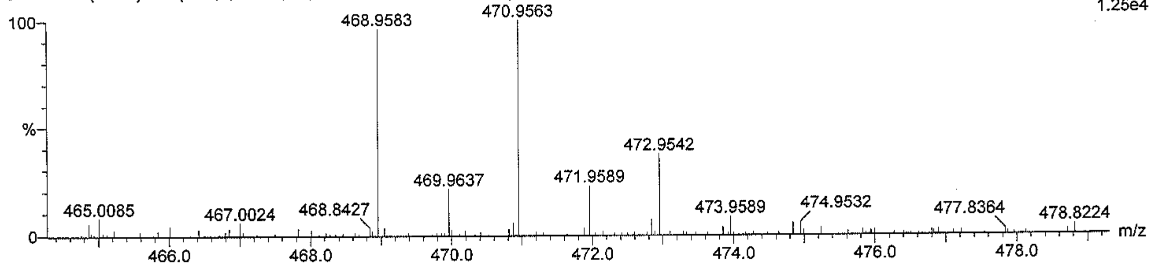

Minimum:				-1.5		
Maximum:		5.0	7.0	50.0		
Mass	Calc. Mass	mDa	PPM	DBE	i-FIT	Formula
468.9583	468.9583	0.0	0.0	13.5	3.7	C19 H12 N2 O4 S Cl3
	468.9584	-0.1	-0.2	17.5	3141.0	C21 H13 N2 O2 S2 80Se
	468.9581	0.2	0.4	19.5	585.7	C23 H8 N2 O2 Na S Cl2
	468.9580	0.3	0.6	3.5	370.3	C14 H23 O4 S2 Cl2 80Se
	468.9588	-0.5	-1.1	28.5	1120.0	C31 H4 Na Cl2
	468.9578	0.5	1.1	9.5	1280.9	C18 H19 O2 Na S2 Cl 80Se
	468.9588	-0.5	-1.1	0.5	57.3	C12 H25 N2 Na S2 Cl3 80Se
	468.9578	0.5	1.1	8.5	111.1	C17 H20 N2 S Cl3 80Se
	468.9590	-0.7	-1.5	22.5	181.6	C27 H8 O2 Cl3
	468.9590	-0.7	-1.5	26.5	3736.4	C29 H9 S 80Se
	468.9593	-1.0	-2.1	5.5	41.0	C14 H17 N2 O4 Na S2 Cl3
	468.9572	1.1	2.3	28.5	5385.8	C29 H2 O4 Na S
	468.9572	1.1	2.3	27.5	959.3	C28 H3 N2 O2 Cl2
	468.9596	-1.3	-2.8	31.5	5430.9	C31 H O4 S
	468.9568	1.5	3.2	17.5	1603.4	C23 H14 O2 S Cl 80Se
	468.9600	-1.7	-3.6	18.5	3237.6	C24 H14 Na S2 80Se
	468.9566	1.7	3.6	19.5	110.8	C25 H9 O2 Na Cl3
	468.9566	1.7	3.6	23.5	3659.1	C27 H10 Na S 80Se
	468.9600	-1.7	-3.6	14.5	33.3	C22 H13 O2 Na S Cl3
	468.9601	-1.8	-3.8	10.5	567.6	C17 H16 N2 O3 Na Cl2 80Se
	468.9602	-1.9	-4.1	12.5	1332.0	C20 H18 O2 S2 Cl 80Se
	468.9603	-2.0	-4.3	28.5	2609.4	C29 H3 N2 Na S Cl
	468.9562	2.1	4.5	8.5	1238.1	C15 H18 N2 O4 S2 Cl 80Se
	468.9605	-2.2	-4.7	22.5	678.2	C25 H7 N2 O2 S Cl2
	468.9605	-2.2	-4.7	23.5	4885.4	C26 H6 O4 Na S2
	468.9560	2.3	4.9	14.5	3102.5	C19 H14 N2 O2 Na S2 80Se
	468.9559	2.4	5.1	10.5	5.8	C17 H13 N2 O4 Na S Cl3
	468.9556	2.7	5.8	31.5	4178.0	C32 H5 80Se
	468.9556	2.7	5.8	0.5	391.5	C12 H24 O4 Na S2 Cl2 80Se
	468.9612	-2.9	-6.2	31.5	1261.0	C33 H3 Cl2
	468.9554	2.9	6.2	5.5	89.6	C15 H21 N2 Na S Cl3 80Se
	468.9612	-2.9	-6.2	3.5	58.1	C14 H24 N2 S2 Cl3 80Se
	468.9615	-3.2	-6.8	14.5	380.0	C20 H12 N2 O2 Na S2 Cl2

Figure S35. HRMS spectrum of **7c**

7c

Elemental Composition Report **Page 1**

Single Mass Analysis (displaying only valid results)
Tolerance = 7.0 PPM / DBE: min = -1.5, max = 50.0
Selected filters: None

Monoisotopic Mass, Even Electron Ions
1886 formula(e) evaluated with 8 results within limits (up to 80 closest results for each mass)
Elements Used:
C: 0-50 H: 0-50 N: 0-3 O: 0-4 Na: 0-1 S: 0-2 Cl: 0-3

Kaul LCT Premier KD070
SK 136 9 (0.217) AM (Cen,4, 66.00, Ar,11000.0,554.26,0.70,LS 5); Cm (6:34) 1: TOF MS ES- 9.77e3

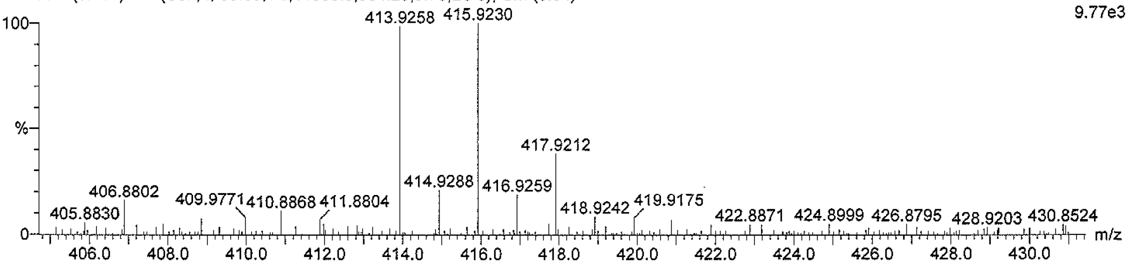

Minimum:				-1.5		
Maximum:		5.0	7.0	50.0		
Mass	Calc. Mass	mDa	PPM	DBE	i-FIT	Formula
413.9258	413.9256	0.2	0.5	18.5	19.8	C21 H4 N O Na Cl3
	413.9250	0.8	1.9	9.5	45.9	C13 H8 N3 O3 Na S Cl3
	413.9272	-1.4	-3.4	18.5	346.4	C19 H3 N3 O Na S Cl2
	413.9274	-1.6	-3.9	12.5	13.7	C15 H7 N3 O3 S Cl3
	413.9240	1.8	4.3	17.5	3.4	C18 H3 N3 O3 Cl3
	413.9239	1.9	4.6	25.5	1630.2	C25 H N S2 Cl
	413.9280	-2.2	-5.3	21.5	55.3	C23 H3 N O Cl3
	413.9283	-2.5	-6.0	4.5	125.8	C10 H12 N3 O3 Na S2 Cl3

Figure S36. HRMS spectrum of **8**

Elemental Composition Report

Single Mass Analysis (displaying only valid results)
Tolerance = 20.0 PPM / DBE: min = -1.5, max = 50.0
Selected filters: None

Monoisotopic Mass, Odd and Even Electron Ions
270 formula(e) evaluated with 15 results within limits (up to 80 closest results for each mass)
Elements Used:
C: 0-25 H: 0-30 N: 0-2 O: 0-8 S: 0-1 Cl: 0-2

Kaul LCT Premier KD070
SK 305 18 (0.405) AM (Cen,5, 60.00, Ar,11000.0,556.28,0.70,LS 5) 1: TOF MS ES+ 5.17e3

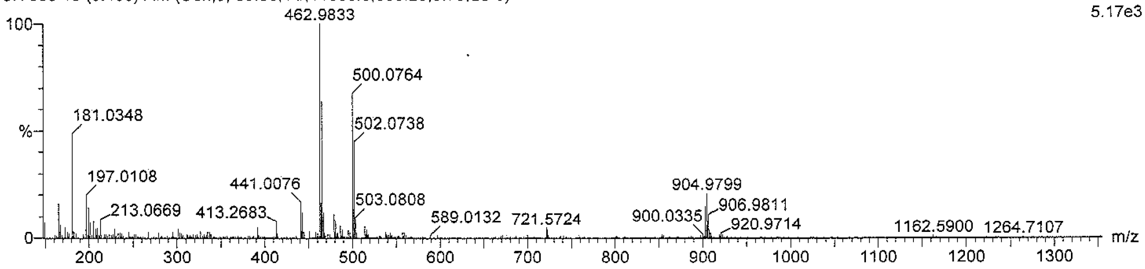

Minimum:				-1.5		
Maximum:		5.0	20.0	50.0		

Mass	Calc. Mass	mDa	PPM	DBE	i-FIT	Formula
441.0076	441.0074	0.2	0.5	16.0	59.3	C21 H12 N O6 S Cl
	441.0079	-0.3	-0.7	11.5	2.0	C18 H15 N2 O5 S Cl2
	441.0069	0.7	1.6	20.5	242.1	C24 H9 O7 S
	441.0052	2.4	5.4	7.0	0.3	C15 H17 N O8 S Cl2
	441.0101	-2.5	-5.7	20.5	73.4	C24 H10 N2 O3 S Cl
	441.0045	3.1	7.0	16.5	7.4	C21 H11 N2 O5 Cl2
	441.0040	3.6	8.2	21.0	84.1	C24 H8 N O6 Cl
	441.0119	-4.3	-9.8	15.5	11.5	C23 H15 O3 S Cl2
	441.0126	-5.0	-11.3	16.5	71.4	C20 H10 N2 O8 Cl
	441.0020	5.6	12.7	20.5	20.3	C25 H11 N2 S Cl2
	441.0018	5.8	13.2	12.0	2.2	C18 H13 N O8 Cl2
	441.0144	-6.8	-15.4	11.5	3.2	C19 H15 O8 Cl2
	440.9993	8.3	18.8	16.0	10.8	C22 H13 N O3 S Cl2
	441.0159	-8.3	-18.8	11.5	50.8	C17 H14 N2 O8 S Cl
	440.9988	8.8	20.0	20.5	75.8	C25 H10 O4 S Cl

Figure S37. HRMS spectrum of **9**

9

Elemental Composition Report

Single Mass Analysis (displaying only valid results)

Tolerance = 20.0 PPM / DBE: min = -1.5, max = 50.0
Selected filters: None

Monoisotopic Mass, Even Electron Ions
1254 formula(e) evaluated with 42 results within limits (up to 80 closest results for each mass)
Elements Used:
C: 0-30 H: 0-40 N: 0-2 O: 0-7 S: 0-2 Cl: 0-2 Na: 0-1

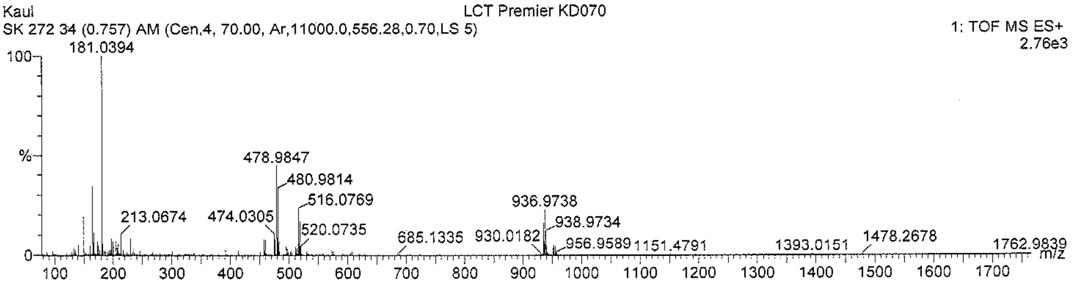

Mass	Calc. Mass	mDa	PPM	DBE	i-FIT	Formula
Minimum:				-1.5		
Maximum:		5.0	20.0	50.0		
478.9847	478.9847	0.0	0.0	11.5	4.4	C18 H14 N2 O6 S Cl2 Na
	478.9846	0.1	0.2	18.5	27.9	C24 H13 N2 O S2 Cl2
	478.9854	-0.7	-1.5	20.5	36.3	C26 H10 O4 Cl2 Na
	478.9838	0.9	1.9	19.5	23.6	C23 H9 N2 O6 Cl2
	478.9837	1.0	2.1	27.5	367.8	C30 H7 O3 S2
	478.9836	1.1	2.3	25.5	165.5	C27 H5 N2 O4 Cl Na
	478.9860	-1.3	-2.7	28.5	179.4	C29 H4 N2 O4 Cl
	478.9869	-2.2	-4.6	20.5	125.2	C24 H9 N2 O4 S Cl Na
	478.9871	-2.4	-5.0	14.5	10.2	C20 H13 N2 O6 S Cl2
	478.9822	2.5	5.2	15.5	18.4	C22 H14 N2 O S2 Cl2 Na
	478.9878	-3.1	-6.5	23.5	49.1	C28 H9 O4 Cl2
	478.9815	3.2	6.7	18.5	102.3	C24 H12 O5 S2 Cl
	478.9814	3.3	6.9	16.5	15.2	C21 H10 N2 O6 Cl2 Na
	478.9813	3.4	7.1	24.5	358.7	C28 H8 O3 S2 Na
	478.9881	-3.4	-7.1	6.5	0.6	C15 H18 N2 O6 S2 Cl2 Na
	478.9813	3.4	7.1	23.5	45.3	C27 H9 N2 O S Cl2
	478.9888	-4.1	-8.6	15.5	20.1	C23 H14 O4 S Cl2 Na
	478.9891	-4.4	-9.2	29.5	411.0	C30 H4 N2 O2 S Na
	478.9893	-4.6	-9.6	23.5	138.7	C26 H8 N2 O4 S Cl
	478.9796	5.1	10.6	23.5	345.4	C25 H7 N2 O5 S2
	478.9793	5.4	11.3	9.5	4.1	C18 H17 O7 S2 Cl2
	478.9903	-5.6	-11.7	15.5	90.3	C21 H13 N2 O4 S2 Cl Na
	478.9791	5.6	11.7	15.5	91.1	C22 H13 O5 S2 Cl Na
	478.9789	5.8	12.1	20.5	33.9	C25 H10 N2 O S Cl2 Na
	478.9905	-5.8	-12.1	9.5	3.6	C17 H17 N2 O6 S2 Cl2
	478.9909	-6.2	-12.9	24.5	157.8	C29 H9 O2 S Cl Na
	478.9912	-6.5	-13.6	18.5	30.8	C25 H13 O4 S Cl2
	478.9781	6.6	13.8	23.5	140.5	C27 H8 O5 S Cl
	478.9779	6.8	14.2	28.5	68.1	C30 H5 N2 O Cl2
	478.9916	-6.9	-14.4	25.5	432.5	C26 H4 N2 O7 Na
	478.9774	7.3	15.2	14.5	78.6	C19 H12 N2 O7 S2 Cl
	478.9921	-7.4	-15.4	10.5	10.0	C20 H18 O4 S2 Cl2 Na
	478.9772	7.5	15.7	20.5	338.8	C23 H8 N2 O5 S2 Na
	478.9925	-7.8	-16.3	24.5	361.0	C27 H8 N2 O2 S2 Na
	478.9769	7.8	16.3	6.5	2.2	C16 H18 O7 S2 Cl2 Na
	478.9927	-8.0	-16.7	18.5	102.7	C23 H12 N2 O4 S2 Cl
	478.9763	8.4	17.5	28.5	397.8	C28 H3 N2 O5 S
	478.9759	8.8	18.4	14.5	13.3	C21 H13 O7 S Cl2
	478.9935	-8.8	-18.4	20.5	148.4	C25 H9 O7 Cl Na
	478.9757	9.0	18.8	20.5	128.3	C25 H9 O5 S Cl Na
	478.9755	9.2	19.2	25.5	55.4	C28 H6 N2 O Cl2 Na

Figure S38. HRMS spectrum of **10a**

Single Mass Analysis

Tolerance = 25.0 PPM / DBE: min = -1.5, max = 50.0
Selected filters: None

Monoisotopic Mass, Odd and Even Electron Ions
3842 formula(e) evaluated with 184 results within limits (up to 50 closest results for each mass)
Elements Used:
C: 0-40 H: 0-50 N: 0-4 O: 0-2 P: 0-2 S: 0-1 Cl: 0-3 Fe: 0-2

Kaul Instrument : Micromass GCT
SK 339 316 (5.267) AM (Cen,4, 90.00, Ar,5000.0,130.99,1.00); Cm (261:329) TOF MS EI+ 777

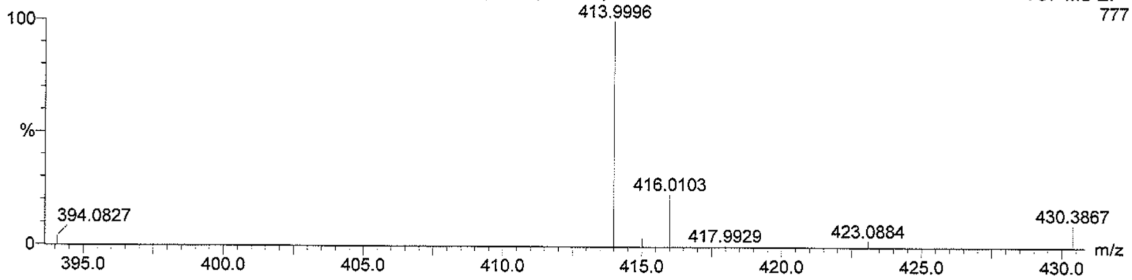

Mass	Calc. Mass	mDa	PPM	DBE	i-FIT	Formula
Minimum:				-1.5		
Maximum:		15.0	25.0	50.0		
413.9996	413.9997	-0.1	-0.2	15.0	180.2	C20 H12 N2 O2 S Cl2
	413.9997	-0.1	-0.2	3.0	184.9	C12 H22 N4 P Cl3 Fe
	413.9995	0.1	0.2	3.0	130.3	C11 H22 N4 O P2 Cl2 Fe
	413.9995	0.1	0.2	9.0	237.0	C15 H19 N4 O P Fe2
	413.9997	-0.1	-0.2	9.0	174.6	C16 H19 N4 Cl Fe2
	413.9994	0.2	0.5	7.5	195.9	C16 H19 N3 Cl3 Fe
	414.0000	-0.4	-1.0	10.5	158.8	C16 H15 N3 O2 P S Cl2
	414.0000	-0.4	-1.0	24.0	279.6	C26 H8 O2 P2
	414.0000	-0.4	-1.0	16.5	250.0	C20 H12 N3 O2 S Fe
	413.9992	0.4	1.0	13.5	266.5	C19 H16 N3 O Fe2
	413.9992	0.4	1.0	7.5	153.2	C15 H19 N3 O P Cl2 Fe
	414.0000	-0.4	-1.0	29.0	287.5	C27 H2 N4 S
	414.0001	-0.5	-1.2	24.0	222.5	C27 H8 O P Cl
	414.0001	-0.5	-1.2	10.5	204.9	C17 H15 N3 O S Cl3
	413.9991	0.5	1.2	20.0	180.6	C21 H9 N4 P2 Cl
	413.9990	0.6	1.4	7.5	127.0	C14 H19 N3 O2 P2 Cl Fe
	413.9989	0.7	1.7	12.0	177.5	C19 H16 N2 O Cl2 Fe
	414.0003	-0.7	-1.7	24.0	217.3	C28 H8 Cl2
	414.0003	-0.7	-1.7	12.0	217.3	C16 H15 N4 O2 P S Fe
	414.0003	-0.7	-1.7	6.0	139.5	C12 H18 N4 O2 P2 S Cl2
	414.0004	-0.8	-1.9	19.5	190.8	C23 H11 N O P2 Cl
	413.9988	0.8	1.9	24.5	214.0	C25 H6 N3 P Cl
	413.9987	0.9	2.2	12.0	166.6	C18 H16 N2 O2 P Cl Fe
	414.0005	-0.9	-2.2	12.0	166.8	C17 H15 N4 O S Cl Fe
	413.9987	0.9	2.2	6.0	189.3	C14 H19 N2 O2 P2 Cl3
	414.0005	-0.9	-2.2	6.0	196.3	C13 H18 N4 O P S Cl3
	414.0006	-1.0	-2.4	13.0	273.5	C21 H18 O2 Fe2
	414.0006	-1.0	-2.4	7.0	160.3	C17 H21 O2 P Cl2 Fe
	414.0006	-1.0	-2.4	25.5	306.0	C28 H8 N Fe
	413.9986	1.0	2.4	24.5	272.8	C24 H6 N3 O P2
	414.0006	-1.0	-2.4	19.5	194.8	C24 H11 N P Cl2
	414.0007	-1.1	-2.7	7.0	199.7	C18 H21 O Cl3 Fe
	413.9985	1.1	2.7	29.0	242.3	C29 H3 N2 Cl
	413.9984	1.2	2.9	16.5	201.7	C22 H13 N O2 Cl Fe
	413.9984	1.2	2.9	10.5	198.9	C18 H16 N O2 P Cl3
	414.0009	-1.3	-3.1	8.5	245.5	C17 H21 N O2 P Fe2
	414.0009	-1.3	-3.1	15.0	171.1	C20 H14 N2 P2 Cl2
	414.0009	-1.3	-3.1	2.5	136.9	C13 H24 N O2 P2 Cl2 Fe
	413.9983	1.3	3.1	29.0	296.3	C28 H3 N2 O P
	414.0009	-1.3	-3.1	21.0	284.8	C24 H11 N2 P Fe
	414.0010	-1.4	-3.4	8.5	184.8	C18 H21 N O Cl Fe2
	413.9981	1.5	3.6	15.0	210.6	C22 H13 O2 Cl3
	414.0011	-1.5	-3.6	2.5	188.0	C14 H24 N O P Cl3 Fe
	413.9980	1.6	3.9	33.5	315.5	C32 N O
	414.0012	-1.6	-3.9	4.0	212.0	C13 H24 N2 O2 P2 Fe2
	414.0012	-1.6	-3.9	16.5	259.0	C20 H14 N3 P2 Fe
	414.0014	-1.8	-4.3	4.0	147.3	C14 H24 N2 O P Cl Fe2

Figure S39. HRMS spectrum of **10b**

10 6

Elemental Composition Report

Single Mass Analysis

Tolerance = 25.0 PPM / DBE: min = -1.5, max = 50.0
Selected filters: None

Monoisotopic Mass, Odd and Even Electron Ions
2700 formula(e) evaluated with 161 results within limits (up to 50 closest results for each mass)
Elements Used:
C: 0-30 H: 0-40 N: 0-4 O: 0-6 S: 0-1 Cl: 0-3 Fe: 0-2

Kaul Instrument : Micromass GCT
SK 340 427 (7.116) AM (Cen,4, 34.00, Ar,5000.0,218.99,1.00); Cm (420:468) TOF MS EI+
464.0153 66.8

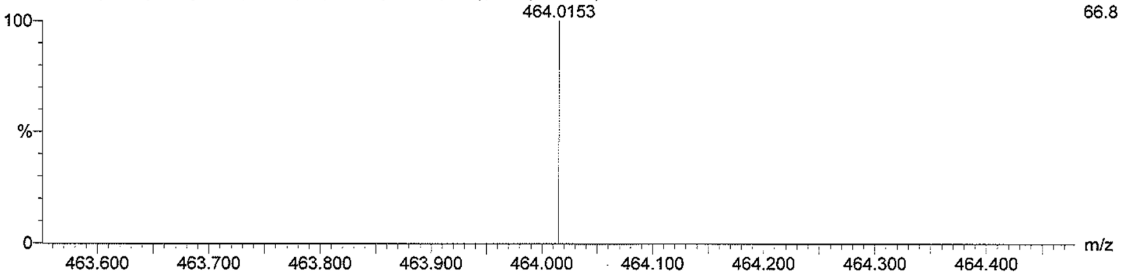

Mass	Calc. Mass	mDa	PPM	DBE	i-FIT	Formula
Minimum:				-1.5		
Maximum:		15.0	25.0	50.0		
464.0153	464.0153	0.0	0.0	18.0	5546056.0	C24 H14 N2 O2 S Cl2
	464.0153	0.0	0.0	5.5	5546056.0	C17 H24 N O4 S Cl2 Fe
	464.0154	-0.1	-0.2	12.0	5546053.5	C20 H21 N4 Cl Fe2
	464.0156	-0.3	-0.6	7.0	5546049.0	C17 H24 N2 O4 S Fe2
	464.0156	-0.3	-0.6	19.5	5546051.5	C24 H14 N3 O2 S Fe
	464.0150	0.3	0.6	10.5	5546057.0	C20 H21 N3 Cl3 Fe
	464.0157	-0.4	-0.9	1.0	5546057.0	C14 H27 N2 O3 S Cl3 Fe
	464.0149	0.4	0.9	16.5	5546051.5	C23 H18 N3 O Fe2
	464.0148	0.5	1.1	10.0	5546053.5	C20 H21 O5 S Cl Fe
	464.0158	-0.5	-1.1	13.5	5546057.0	C21 H17 N3 O S Cl3
	464.0148	0.5	1.1	22.5	5546054.0	C27 H11 N O3 S Cl
	464.0146	0.7	1.5	15.0	5546056.0	C23 H18 N2 O Cl2 Fe
	464.0160	-0.7	-1.5	2.5	5546053.5	C14 H27 N3 O3 S Cl Fe2
	464.0145	0.8	1.7	2.5	5546056.0	C16 H28 N O3 Cl2 Fe2
	464.0161	-0.8	-1.7	15.0	5546054.0	C21 H17 N4 O S Cl Fe
	464.0162	-0.9	-1.9	16.0	5546052.0	C25 H20 O2 Fe2
	464.0143	1.0	2.2	27.0	5546052.0	C30 H8 O4 S
	464.0164	-1.1	-2.4	10.0	5546057.0	C22 H23 O Cl3 Fe
	464.0141	1.2	2.6	19.5	5546054.0	C26 H15 N O2 Cl Fe
	464.0140	1.3	2.8	7.0	5546053.0	C19 H25 O4 Cl Fe2
	464.0167	-1.4	-3.0	11.5	5546053.5	C22 H23 N O Cl Fe2
	464.0139	1.4	3.0	6.0	5546056.0	C15 H22 N4 O3 S Cl2 Fe
	464.0138	1.5	3.2	18.0	5546057.0	C26 H15 O2 Cl3
	464.0136	1.7	3.7	24.0	5546052.5	C29 H12 O3 Fe
	464.0170	-1.7	-3.7	19.0	5546051.5	C26 H16 O3 S Fe
	464.0171	-1.8	-3.9	13.0	5546057.0	C23 H19 O2 S Cl3
	464.0135	1.8	3.9	23.0	5546054.0	C25 H9 N4 O2 S Cl
	464.0134	1.9	4.1	10.5	5546053.5	C18 H19 N3 O4 S Cl Fe
	464.0172	-1.9	-4.1	7.0	5546056.0	C19 H26 N2 Cl2 Fe2
	464.0174	-2.1	-4.5	14.5	5546054.0	C23 H19 N O2 S Cl Fe
	464.0132	2.1	4.5	3.0	5546055.5	C14 H26 N4 O2 Cl2 Fe2
	464.0174	-2.1	-4.5	2.0	5546053.5	C16 H29 O4 S Cl Fe2
	464.0175	-2.2	-4.7	27.0	5546054.5	C30 H9 N2 S Cl
	464.0131	2.2	4.7	9.0	5546057.0	C18 H19 N2 O4 S Cl3
	464.0130	2.3	5.0	27.5	5546052.0	C28 H6 N3 O3 S
	464.0129	2.4	5.2	15.0	5546050.0	C21 H16 N2 O5 S Fe
	464.0179	-2.6	-5.6	10.0	5546056.0	C20 H22 N2 O S Cl2 Fe
	464.0127	2.6	5.6	20.0	5546053.5	C24 H13 N4 O Cl Fe
	464.0127	2.6	5.6	7.5	5546053.0	C17 H23 N3 O3 Cl Fe2
	464.0126	2.7	5.8	13.5	5546056.0	C21 H16 N O5 S Cl2
	464.0182	-2.9	-6.2	11.5	5546050.5	C20 H22 N3 O S Fe2
	464.0124	2.9	6.2	6.0	5546057.0	C17 H23 N2 O3 Cl3 Fe
	464.0182	-2.9	-6.2	28.0	5546051.5	C27 H4 N4 O5
	464.0124	2.9	6.2	18.5	5546057.0	C24 H13 N3 O Cl3
	464.0183	-3.0	-6.5	9.5	5546057.0	C17 H17 N3 O6 Cl3
	464.0122	3.1	6.7	24.5	5546052.0	C27 H10 N3 O2 Fe
	464.0122	3.1	6.7	12.0	5546050.0	C20 H20 N2 O4 Fe2

Figure S40. HRMS spectrum of **10c**

10c

Elemental Composition Report

Single Mass Analysis
Tolerance = 10.0 PPM / DBE: min = -1.5, max = 50.0
Selected filters: None

Monoisotopic Mass, Odd and Even Electron Ions
1011 formula(e) evaluated with 19 results within limits (up to 50 closest results for each mass)
Elements Used:
C: 0-40 H: 0-50 N: 0-4 O: 0-5 S: 0-1 Cl: 0-3
Schmiel Instrument : Micromass GCT
SK309 383 (6.384) AM (Cen,4, 50.00, Ar,5000.0,218.99,1.00); Cm (382:446) TOF MS EI+ 3.09e3

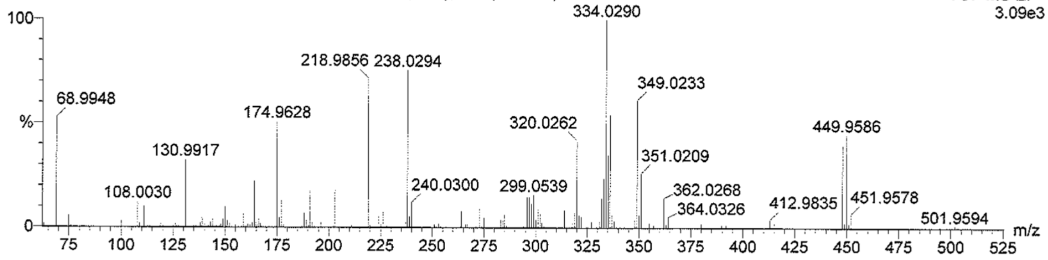

Minimum:				-1.5		
Maximum:		15.0	10.0	50.0		
Mass	Calc. Mass	mDa	PPM	DBE	i-FIT	Formula
447.9607	447.9607	0.0	0.0	15.0	308.8	C20 H11 N2 O2 S Cl3
	447.9610	-0.3	-0.7	29.0	759.8	C27 H N4 S Cl
	447.9602	0.5	1.1	19.5	496.0	C23 H8 N O3 S Cl2
	447.9613	-0.6	-1.3	24.0	474.7	C28 H7 Cl3
	447.9597	1.0	2.2	24.0	719.2	C26 H5 O4 S Cl
	447.9619	-1.2	-2.7	33.0	967.6	C32 O2 S
	447.9595	1.2	2.7	29.0	635.3	C29 H2 N2 Cl2
	447.9624	-1.7	-3.8	28.5	775.6	C29 H3 N O S Cl
	447.9589	1.8	4.0	20.0	471.4	C21 H6 N4 O2 S Cl2
	447.9629	-2.2	-4.9	24.0	570.4	C26 H6 N2 S Cl2
	447.9584	2.3	5.1	24.5	701.1	C24 H3 N3 O3 S Cl
	447.9580	2.7	6.0	10.5	229.4	C17 H13 N O5 S Cl3
	447.9635	-2.8	-6.3	25.0	699.9	C23 H N4 O5 Cl
	447.9579	2.8	6.3	29.0	923.8	C27 N2 O4 S
	447.9573	3.4	7.6	20.0	377.1	C23 H7 N2 O2 Cl3
	447.9568	3.9	8.7	24.5	568.2	C26 H4 N O3 Cl2
	447.9647	-4.0	-8.9	19.0	411.1	C25 H11 S Cl3
	447.9567	4.0	8.9	11.0	204.4	C15 H11 N4 O4 S Cl3
	447.9563	4.4	9.8	29.0	781.3	C29 H O4 Cl

Figure S41. HRMS spectrum of **10d**

Elemental Composition Report

10d

Single Mass Analysis

Tolerance = 10.0 PPM / DBE: min = -1.5, max = 50.0
Selected filters: None

Monoisotopic Mass, Odd and Even Electron Ions
10764 formula(e) evaluated with 237 results within limits (up to 50 closest results for each mass)
Elements Used:
C: 0-40 H: 0-50 N: 0-4 O: 0-5 S: 0-2 Cl: 0-3 Fe: 0-2 F: 0-2

Instrument : Micromass GCT
TOF MS EI+

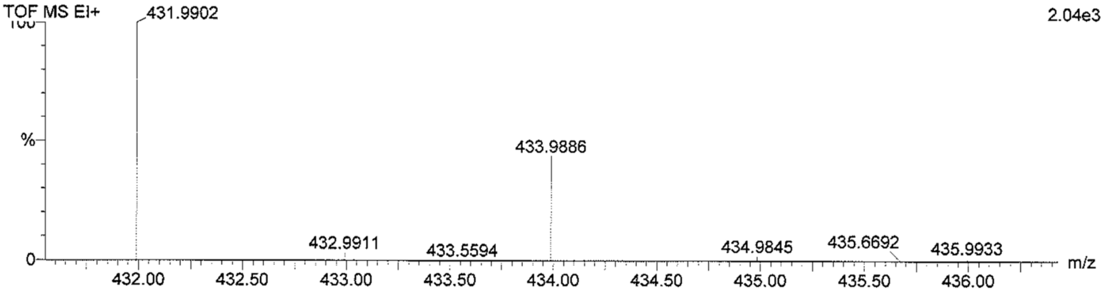

Minimum:				-1.5		
Maximum:		15.0	10.0	50.0		

Mass	Calc. Mass	mDa	PPM	DBE	i-FIT	Formula
431.9902	431.9902	0.0	0.0	11.0	542.0	C21 H19 Cl3 Fe
	431.9902	0.0	0.0	25.0	817.1	C26 H3 N2 O Cl F2
	431.9902	0.0	0.0	2.5	367.9	C13 H21 N O4 S Cl2 Fe F
	431.9902	0.0	0.0	15.0	527.0	C20 H11 N2 O2 S Cl2 F
	431.9901	0.1	0.2	0.0	496.4	C12 H23 O5 Cl Fe2 F2
	431.9903	-0.1	-0.2	9.0	684.6	C16 H18 N4 Cl Fe2 F
	431.9901	0.1	0.2	11.0	836.2	C17 H16 N2 O4 S2 Fe
	431.9903	-0.1	-0.2	5.0	393.6	C14 H19 N2 O3 S2 Cl3
	431.9901	0.1	0.2	12.5	677.3	C19 H13 N O3 Cl Fe F2
	431.9901	0.1	0.2	23.5	954.7	C24 H6 N3 O2 S2
	431.9903	-0.1	-0.2	-1.0	370.7	C10 H26 N4 O S Cl2 Fe2
	431.9900	0.2	0.5	-1.0	236.8	C8 H20 N4 O4 S Cl2 Fe F2
	431.9904	-0.2	-0.5	20.0	929.8	C23 H6 O5 S F2
	431.9900	0.2	0.5	17.0	1043.0	C24 H16 O Fe2
	431.9900	0.2	0.5	7.5	443.6	C16 H18 N3 Cl3 Fe F
	431.9905	-0.3	-0.7	4.0	805.0	C13 H21 N2 O4 S Fe2 F
	431.9905	-0.3	-0.7	16.5	933.7	C20 H11 N3 O2 S Fe F
	431.9899	0.3	0.7	16.0	732.9	C20 H13 N4 S Cl Fe
	431.9905	-0.3	-0.7	12.5	798.2	C21 H19 N Cl Fe2
	431.9898	0.4	0.9	11.0	446.0	C19 H13 O3 Cl3 F2
	431.9898	0.4	0.9	9.5	444.4	C17 H16 N O4 S2 Cl2
	431.9906	-0.4	-0.9	29.0	1028.6	C27 H N4 S F
	431.9898	0.4	0.9	13.5	975.9	C19 H15 N3 O Fe2 F
	431.9906	-0.4	-0.9	6.5	529.9	C14 H19 N3 O3 S2 Cl Fe
	431.9906	-0.4	-0.9	19.0	694.7	C21 H9 N4 O S2 Cl
	431.9906	-0.4	-0.9	8.0	457.8	C16 H16 N2 O2 Cl2 Fe F2
	431.9898	0.4	0.9	3.5	572.2	C13 H23 N3 O2 S Cl Fe2
	431.9907	-0.5	-1.2	10.5	440.7	C17 H14 N3 O S Cl3 F
	431.9897	0.5	1.2	17.0	972.2	C22 H10 O4 Fe F2
	431.9897	0.5	1.2	19.5	726.0	C23 H8 N O3 S Cl F
	431.9907	-0.5	-1.2	20.0	1008.4	C25 H12 O2 S Fe
	431.9907	-0.5	-1.2	2.0	234.1	C9 H16 N4 O5 S2 Cl2 F2
	431.9897	0.5	1.2	7.0	564.1	C16 H18 O5 S Cl Fe F
	431.9897	0.5	1.2	29.5	1059.2	C29 N O2 F2
	431.9907	-0.5	-1.2	20.5	610.8	C23 H6 N3 Cl2 F2
	431.9896	0.6	1.4	6.0	320.8	C12 H15 N4 O4 S2 Cl2 F
	431.9908	-0.6	-1.4	32.5	1083.1	C32 H2 N S
	431.9896	0.6	1.4	14.5	507.4	C20 H13 N3 S Cl3
	431.9895	0.7	1.6	16.0	599.3	C18 H7 N4 O3 S Cl F2
	431.9895	0.7	1.6	2.0	387.9	C13 H23 N2 O2 S Cl3 Fe
	431.9909	-0.7	-1.6	5.5	353.7	C14 H17 N O5 S2 Cl2 F
	431.9909	-0.7	-1.6	14.0	531.3	C22 H15 O S Cl3
	431.9895	0.7	1.6	3.5	406.5	C11 H17 N3 O5 S Cl Fe F2
	431.9895	0.7	1.6	12.0	552.7	C19 H15 N2 O Cl2 Fe F

Figure S42. HRMS spectrum of **11a**

11a

Elemental Composition Report

Single Mass Analysis

Tolerance = 25.0 PPM / DBE: min = -1.5, max = 50.0
Selected filters: None

Monoisotopic Mass, Odd and Even Electron Ions
3918 formula(e) evaluated with 143 results within limits (up to 50 closest results for each mass)
Elements Used:
C: 0-40 H: 0-50 N: 0-4 O: 0-6 P: 0-2 Cl: 0-3 Fe: 0-2
Kaul Instrument : Micromass GCT
SK 327 100 (1.666) AM (Cen,4, 19.00, Ar,5000.0,130.99,1.00) TOF MS EI+

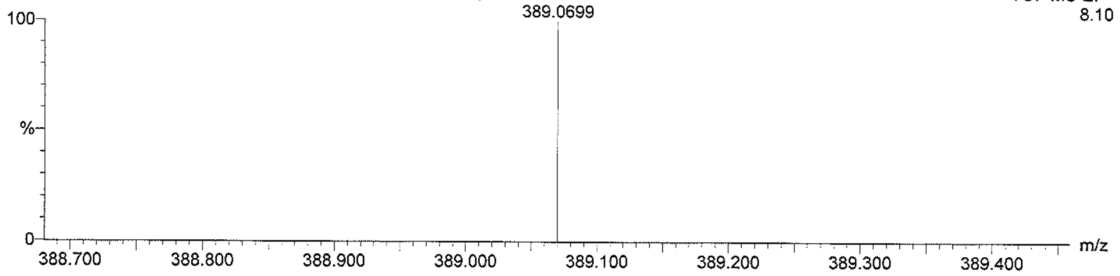

Mass	Calc. Mass	mDa	PPM	DBE	i-FIT	Formula
Minimum:				-1.5		
Maximum:		15.0	25.0	50.0		
389.0699	389.0699	0.0	0.0	7.5	5546026.0	C14 H20 N4 O3 P2 Cl
	389.0698	0.1	0.3	12.0	5546027.0	C19 H17 N3 O2 Cl2
	389.0700	-0.1	-0.3	1.0	5546025.5	C12 H27 N3 O4 Fe2
	389.0701	-0.2	-0.5	7.5	5546027.0	C15 H20 N4 O2 P Cl2
	389.0701	-0.2	-0.5	13.5	5546025.5	C19 H17 N4 O2 Fe
	389.0697	0.2	0.5	-0.5	5546027.0	C12 H27 N2 O4 Cl2 Fe
	389.0696	0.3	0.8	12.0	5546026.0	C18 H17 N3 O3 P Cl
	389.0696	0.3	0.8	-0.5	5546026.0	C11 H27 N2 O5 P Cl Fe
	389.0703	-0.4	-1.0	7.5	5546028.0	C16 H20 N4 O Cl3
	389.0694	0.5	1.3	12.0	5546025.5	C17 H17 N3 O4 P2
	389.0694	0.5	1.3	-0.5	5546025.5	C10 H27 N2 O6 P2 Fe
	389.0693	0.6	1.5	16.5	5546026.0	C22 H14 N2 O3 Cl
	389.0692	0.7	1.8	4.0	5546026.0	C15 H24 N O5 Cl Fe
	389.0691	0.8	2.1	4.0	5546025.5	C14 H24 N O6 P Fe
	389.0691	0.8	2.1	16.5	5546025.5	C21 H14 N2 O4 P
	389.0708	-0.9	-2.3	11.5	5546025.5	C19 H19 O5 P2
	389.0689	1.0	2.6	2.5	5546028.0	C15 H24 O5 Cl3
	389.0710	-1.1	-2.8	11.5	5546026.0	C20 H19 O4 P Cl
	389.0688	1.1	2.8	21.0	5546026.0	C25 H11 N O4
	389.0688	1.1	2.8	2.5	5546027.0	C14 H24 O6 P Cl2
	389.0688	1.1	2.8	8.5	5546025.5	C18 H21 O6 Fe
	389.0711	-1.2	-3.1	11.5	5546027.0	C21 H19 O3 Cl2
	389.0713	-1.4	-3.6	7.0	5546026.0	C16 H22 N O4 P2 Cl
	389.0714	-1.5	-3.9	0.5	5546025.5	C14 H29 O5 Fe2
	389.0714	-1.5	-3.9	7.0	5546027.0	C17 H22 N O3 P Cl2
	389.0714	-1.5	-3.9	13.0	5546025.5	C21 H19 N O3 Fe
	389.0715	-1.6	-4.1	25.5	5546026.0	C28 H9 N2 O
	389.0716	-1.7	-4.4	7.0	5546028.0	C18 H22 N O2 Cl3
	389.0717	-1.8	-4.6	8.5	5546025.5	C17 H22 N2 O3 P Fe
	389.0718	-1.9	-4.9	21.0	5546026.0	C24 H12 N3 O P
	389.0718	-1.9	-4.9	2.5	5546027.0	C13 H25 N2 O3 P2 Cl2
	389.0719	-2.0	-5.1	8.5	5546026.0	C18 H22 N2 O2 Cl Fe
	389.0679	2.0	5.1	-1.5	5546028.0	C9 H25 N4 O4 P Cl3
	389.0719	-2.0	-5.1	2.5	5546028.0	C14 H25 N2 O2 P Cl3
	389.0679	2.0	5.1	4.5	5546026.0	C13 H22 N4 O4 Cl Fe
	389.0720	-2.1	-5.4	21.0	5546026.0	C25 H12 N3 Cl
	389.0677	2.2	5.7	-1.5	5546027.5	C8 H25 N4 O5 P2 Cl2
	389.0677	2.2	5.7	4.5	5546025.5	C12 H22 N4 O5 P Fe
	389.0721	-2.2	-5.7	16.5	5546025.5	C20 H15 N4 O P2
	389.0721	-2.2	-5.7	4.0	5546025.5	C13 H25 N3 O3 P2 Fe
	389.0676	2.3	5.9	3.0	5546028.0	C13 H22 N3 O4 Cl3
	389.0722	-2.3	-5.9	4.0	5546026.0	C14 H25 N3 O2 P Cl Fe
	389.0723	-2.4	-6.2	16.5	5546026.0	C21 H15 N4 P Cl
	389.0675	2.4	6.2	21.5	5546025.5	C23 H9 N4 O3
	389.0674	2.5	6.4	9.0	5546025.5	C16 H19 N3 O5 Fe
	389.0674	2.5	6.4	3.0	5546027.0	C12 H22 N3 O5 P Cl2
	389.0724	-2.5	-6.4	4.0	5546027.0	C15 H25 N3 O Cl2 Fe

Figure S43. HRMS spectrum of **11b**

Elemental Composition Report   11 b   Page 1

Single Mass Analysis

Tolerance = 20.0 PPM / DBE: min = -1.5, max = 50.0
Selected filters: None

Monoisotopic Mass, Odd and Even Electron Ions
584 formula(e) evaluated with 22 results within limits (up to 50 closest results for each mass)
Elements Used:
C: 0-40 H: 0-50 N: 0-5 O: 0-4 Cl: 0-4
Instrument : Micromass GCT   SK 317 2 120 (2.000) AM (Cen,4, 52.00, Ar,5000.0,218.99,1.00)
TOF MS EI+   13.2

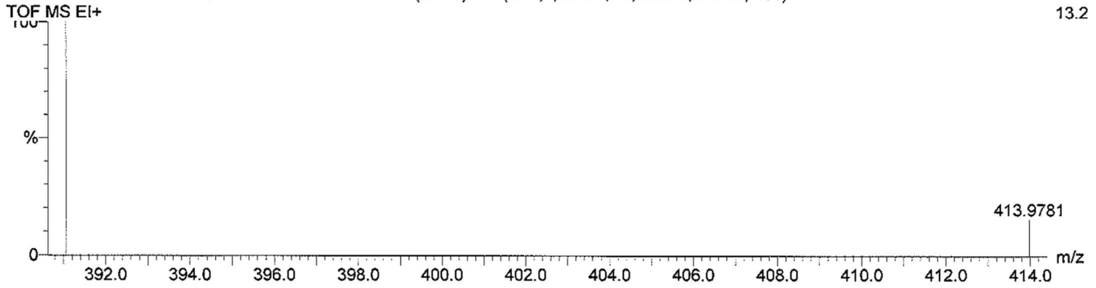

Mass	Calc. Mass	mDa	PPM	DBE	i-FIT	Formula
Minimum:				-1.5		
Maximum:		15.0	20.0	50.0		
391.0490	391.0490	0.0	0.0	12.0	5546029.5	C18 H15 N3 O3 Cl2
	391.0486	0.4	1.0	16.5	5546027.5	C21 H12 N2 O4 Cl
	391.0494	-0.4	-1.0	26.0	5546027.0	C25 H5 N5 O
	391.0495	-0.5	-1.3	7.5	5546030.5	C15 H18 N4 O2 Cl3
	391.0500	-1.0	-2.6	3.0	5546031.0	C12 H21 N5 O Cl4
	391.0504	-1.4	-3.6	11.5	5546029.5	C20 H17 O4 Cl2
	391.0473	1.7	4.3	-1.5	5546031.0	C9 H23 N4 O4 Cl4
	391.0472	1.8	4.6	17.0	5546027.5	C19 H10 N5 O3 Cl
	391.0508	-1.8	-4.6	25.5	5546027.5	C27 H7 N2 O2
	391.0509	-1.9	-4.9	7.0	5546030.5	C17 H20 N O3 Cl3
	391.0512	-2.2	-5.6	21.0	5546028.0	C24 H10 N3 O Cl
	391.0467	2.3	5.9	21.5	5546027.0	C22 H7 N4 O4
	391.0514	-2.4	-6.1	2.5	5546031.0	C14 H23 N2 O2 Cl4
	391.0517	-2.7	-6.9	16.5	5546029.5	C21 H13 N4 Cl2
	391.0526	-3.6	-9.2	20.5	5546028.0	C26 H12 O2 Cl
	391.0531	-4.1	-10.5	16.0	5546029.5	C23 H15 N O Cl2
	391.0536	-4.6	-11.8	11.5	5546030.5	C20 H18 N2 Cl3
	391.0548	-5.8	-14.8	29.5	5546027.5	C32 H7
	391.0428	6.2	15.9	7.0	5546031.0	C18 H21 N Cl4
	391.0554	-6.4	-16.4	6.5	5546031.0	C19 H23 Cl4
	391.0423	6.7	17.1	11.5	5546030.5	C21 H18 O Cl3
	391.0422	6.8	17.4	30.0	5546027.5	C31 H5 N

Figure S44. HRMS spectrum of **12**

12

Elemental Composition Report

Page 1

Single Mass Analysis (displaying only valid results)

Tolerance = 7.0 PPM / DBE: min = -1.5, max = 50.0
Selected filters: None

Monoisotopic Mass, Even Electron Ions
14142 formula(e) evaluated with 113 results within limits (up to 80 closest results for each mass)
Elements Used:
C: 0-50 H: 0-60 N: 0-5 O: 0-7 Na: 0-1 S: 0-2 Cl: 0-3 F: 0-2

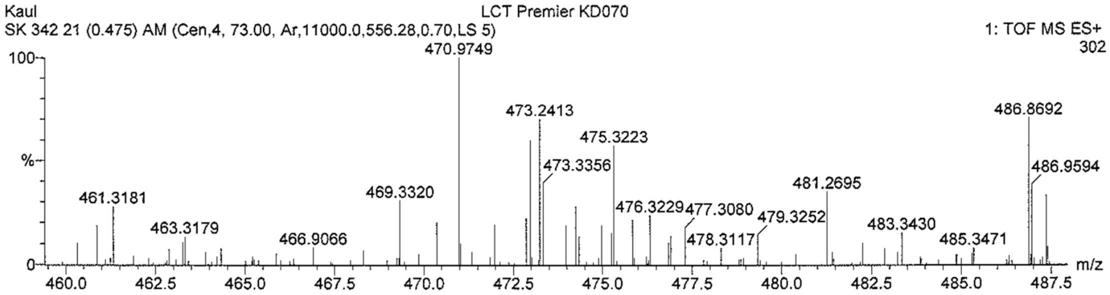

Mass	Calc. Mass	mDa	PPM	DBE	i-FIT	Formula
Minimum:				-1.5		
Maximum:		5.0	7.0	50.0		
470.9749	470.9749	0.0	0.0	14.5	2.8	C20 H11 N2 O3 Na S Cl2 F
	470.9749	0.0	0.0	24.5	22.0	C26 H3 N2 O2 Na Cl F2
	470.9750	-0.1	-0.2	26.5	66.1	C29 H5 O S2 F2
	470.9750	-0.1	-0.2	4.5	22.8	C14 H19 N2 O4 Na S2 Cl3
	470.9750	-0.1	-0.2	35.5	35.2	C34 N2 Cl
	470.9751	-0.2	-0.4	8.5	19.3	C16 H15 N2 O5 S Cl3 F
	470.9751	-0.2	-0.4	19.5	68.8	C23 H6 O6 Na S F2
	470.9751	-0.2	-0.4	9.5	5.2	C17 H14 O7 Na S2 Cl F
	470.9751	-0.2	-0.4	18.5	2.7	C22 H7 N2 O4 Cl2 F2
	470.9746	0.3	0.6	21.5	59.1	C23 H7 N2 O6 S2
	470.9746	0.3	0.6	21.5	16.9	C27 H10 O2 Cl3
	470.9752	-0.3	-0.6	30.5	75.8	C31 H3 O4 S
	470.9745	0.4	0.8	-0.5	27.9	C8 H19 N4 O7 S2 Cl3 F
	470.9745	0.4	0.8	10.5	15.8	C19 H13 O4 Na Cl3 F2
	470.9753	-0.4	-0.8	28.5	74.6	C27 H N4 O Na S F
	470.9753	-0.4	-0.8	18.5	10.0	C21 H9 N4 O2 Na S2 Cl
	470.9744	0.5	1.1	27.5	11.7	C31 H6 Na Cl2
	470.9744	0.5	1.1	26.5	19.4	C26 H4 N4 O2 S Cl
	470.9744	0.5	1.1	9.5	2.4	C14 H11 N4 O6 S Cl2 F2
	470.9754	-0.5	-1.1	1.5	6.8	C9 H16 N4 O6 Na S2 Cl2 F2
	470.9744	0.5	1.1	19.5	15.0	C20 H5 N4 O7 Na Cl
	470.9743	0.6	1.3	5.5	4.5	C12 H15 N4 O5 Na S2 Cl2 F
	470.9755	-0.6	-1.3	22.5	15.1	C23 H5 N4 O3 S Cl F
	470.9755	-0.6	-1.3	12.5	3.7	C17 H13 N4 O4 S2 Cl2
	470.9756	-0.7	-1.5	13.5	18.0	C22 H15 O2 Na S Cl3
	470.9756	-0.7	-1.5	23.5	7.8	C28 H7 O Na Cl2 F
	470.9742	0.7	1.5	15.5	10.0	C18 H7 N4 O4 Na S Cl F2
	470.9742	0.7	1.5	17.5	11.9	C22 H9 O7 S Cl F
	470.9741	0.8	1.7	27.5	82.3	C28 H O6 F2
	470.9741	0.8	1.7	32.5	77.7	C30 N4 Na S
	470.9757	-0.8	-1.7	17.5	56.7	C20 H8 N2 O7 S2 F
	470.9740	0.9	1.9	13.5	7.3	C20 H13 O6 Na S2 Cl
	470.9758	-0.9	-1.9	17.5	15.8	C24 H11 O3 Cl3 F
	470.9740	0.9	1.9	22.5	5.3	C25 H6 N2 O3 Cl2 F
	470.9740	0.9	1.9	12.5	18.3	C19 H14 N2 O4 S Cl3
	470.9739	1.0	2.1	1.5	23.0	C11 H17 N2 O6 Na S Cl3 F2
	470.9759	-1.0	-2.1	15.5	15.0	C20 H9 N4 Na Cl3 F2
	470.9739	1.0	2.1	23.5	71.1	C26 H5 O5 Na S F
	470.9738	1.1	2.3	8.5	21.1	C17 H16 N2 O S2 Cl3 F2
	470.9760	-1.1	-2.3	27.5	23.4	C29 H5 N2 Na S Cl
	470.9738	1.1	2.3	18.5	4.7	C23 H10 N2 O2 Na S Cl2

Figure S45. HRMS spectrum of **13**

13

Elemental Composition Report

Single Mass Analysis (displaying only valid results)

Tolerance = 20.0 PPM / DBE: min = -1.5, max = 50.0
Selected filters: None

Monoisotopic Mass, Odd and Even Electron Ions
642 formula(e) evaluated with 19 results within limits (up to 80 closest results for each mass)
Elements Used:
C: 0-40 H: 0-60 N: 0-3 O: 0-5 S: 0-1 Cl: 0-2

Kaul LCT Premier KD070
SK 278 10 (0.228) AM (Cen,4, 70.00, Ar,11000.0,556.28,0.70,LS 5) 1: TOF MS ES+ 4.31e3

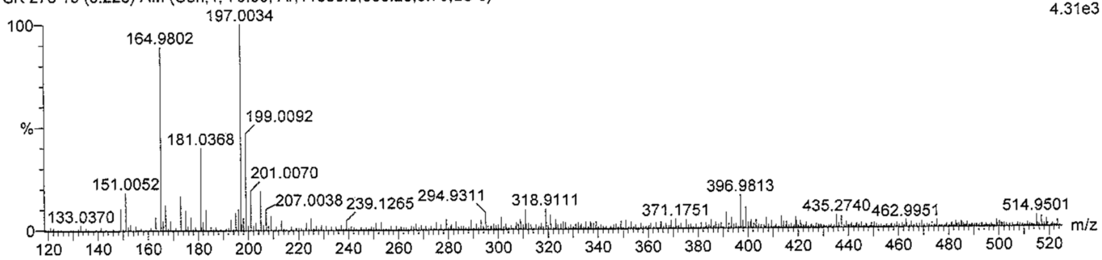

Mass	Calc. Mass	mDa	PPM	DBE	i-FIT	Formula
Minimum:				-1.5		
Maximum:		5.0	20.0	50.0		
396.9813	396.9812	0.1	0.3	16.0	28.1	C19 H8 N O5 S Cl
	396.9810	0.3	0.8	21.0	5.6	C22 H5 N3 O Cl2
	396.9817	-0.4	-1.0	11.5	2.3	C16 H11 N2 O4 S Cl2
	396.9805	0.8	2.0	25.5	53.8	C25 H2 N2 O2 Cl
	396.9823	-1.0	-2.5	20.5	7.6	C24 H7 O2 Cl2
	396.9793	2.0	5.0	21.0	167.3	C20 H3 N3 O5 S
	396.9834	-2.1	-5.3	25.0	173.9	C25 H3 N O3 S
	396.9839	-2.6	-6.5	20.5	36.0	C22 H6 N2 O2 S Cl
	396.9783	3.0	7.6	16.5	1.8	C19 H7 N2 O4 Cl2
	396.9843	-3.0	-7.6	16.0	3.5	C19 H9 N3 O S Cl2
	396.9845	-3.2	-8.1	29.5	69.3	C30 H2 Cl
	396.9778	3.5	8.8	21.0	44.2	C22 H4 N O5 Cl
	396.9857	-4.4	-11.1	15.5	4.9	C21 H11 O2 S Cl2
	396.9860	-4.7	-11.8	29.5	182.8	C28 H N2 S
	396.9753	6.0	15.1	25.0	48.3	C26 H4 N S Cl
	396.9748	6.5	16.4	29.5	182.7	C29 H O S
	396.9879	-6.6	-16.6	24.5	50.3	C27 H6 S Cl
	396.9885	-7.2	-18.1	25.5	198.0	C24 H N2 O5
	396.9890	-7.7	-19.4	21.0	44.6	C21 H4 N3 O4 Cl

Figure S46. HRMS spectrum of **14**

Elemental Composition Report

Single Mass Analysis (displaying only valid results)
Tolerance = 20.0 PPM / DBE: min = -1.5, max = 50.0
Selected filters: None

Monoisotopic Mass, Odd and Even Electron Ions
374 formula(e) evaluated with 15 results within limits (up to 80 closest results for each mass)
Elements Used:
C: 0-25 H: 0-30 N: 0-3 O: 0-5 Na: 0-1 Cl: 0-2

Kaul LCT Premier KD070
SK 273 9 (0.211) AM (Cen,4, 40.00, Ar,11000.0,556.28,0.70,LS 5) 1: TOF MS ES+ 2.93e3

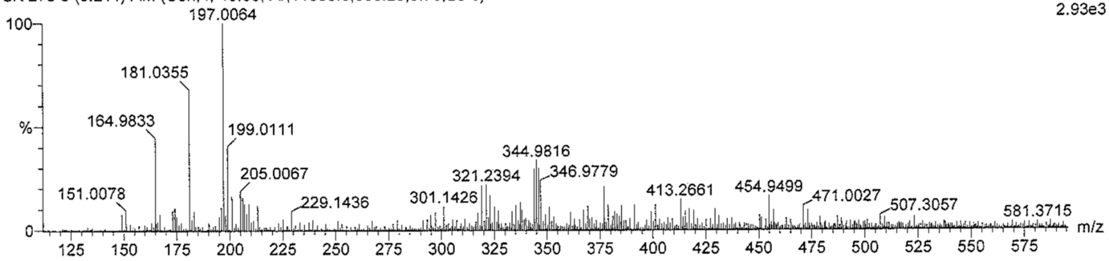

Minimum:				-1.5		
Maximum:		5.0	20.0	50.0		
Mass	Calc. Mass	mDa	PPM	DBE	i-FIT	Formula
344.9816	344.9810	0.6	1.7	10.5	289.2	C14 H8 N2 O3 Na Cl2
	344.9824	-0.8	-2.3	22.5	526.1	C22 H O5
	344.9805	1.1	3.2	15.0	321.5	C17 H5 N O4 Na Cl
	344.9827	-1.1	-3.2	24.0	518.5	C23 N O2 Na
	344.9829	-1.3	-3.8	18.0	298.6	C19 H4 N O4 Cl
	344.9800	1.6	4.6	19.5	550.1	C20 H2 O5 Na
	344.9832	-1.6	-4.6	19.5	288.5	C20 H3 N2 O Na Cl
	344.9834	-1.8	-5.2	13.5	269.3	C16 H7 N2 O3 Cl2
	344.9836	-2.0	-5.8	15.0	257.8	C17 H6 N3 Na Cl2
	344.9787	2.9	8.4	20.0	565.8	C18 N3 O4 Na
	344.9850	-3.4	-9.9	14.5	247.8	C19 H8 O Na Cl2
	344.9856	-4.0	-11.6	22.5	267.3	C22 H2 N2 O Cl
	344.9861	-4.5	-13.0	18.0	239.7	C19 H5 N3 Cl2
	344.9874	-5.8	-16.8	17.5	230.6	C21 H7 O Cl2
	344.9748	6.8	19.7	18.0	244.3	C20 H5 N O Cl2

Figure S47. HRMS spectrum of **15b**

Elemental Composition Report 158

Single Mass Analysis

Tolerance = 10.0 PPM / DBE: min = -1.5, max = 50.0
Selected filters: None

Monoisotopic Mass, Odd and Even Electron Ions
2827 formula(e) evaluated with 57 results within limits (up to 50 closest results for each mass)
Elements Used:
C: 0-40 H: 0-50 N: 0-4 O: 0-5 S: 0-1 Cl: 0-3 Br: 0-2

Instrument : Micromass GCT
TOF MS EI+

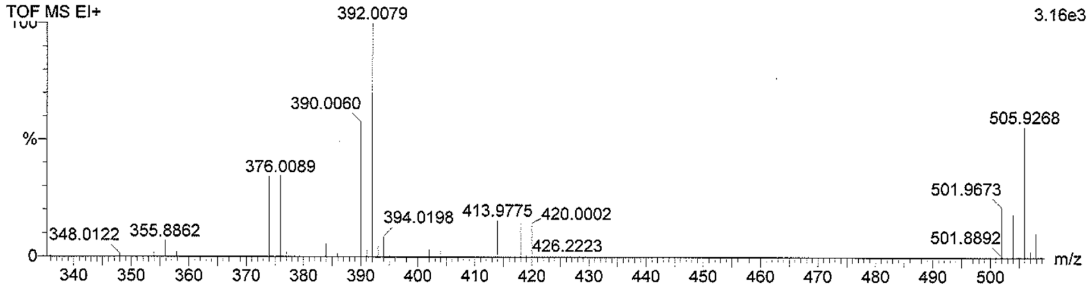

Mass	Calc. Mass	mDa	PPM	DBE	i-FIT	Formula
Minimum:				-1.5		
Maximum:		15.0	10.0	50.0		
503.9280	503.9279	0.1	0.2	16.0	397.7	C21 H11 N2 O4 Cl2 Br
	503.9278	0.2	0.4	4.5	123.1	C15 H22 N3 S Cl2 Br2
	503.9283	-0.3	-0.6	30.0	724.4	C28 H N4 O2 Br
	503.9276	0.4	0.8	27.0	798.4	C26 H2 N4 O2 S Cl2
	503.9284	-0.4	-0.8	11.5	266.2	C18 H14 N3 O3 Cl3 Br
	503.9284	-0.4	-0.8	31.0	928.2	C31 H O4 S Cl
	503.9274	0.6	1.2	20.5	543.8	C24 H8 N O5 Cl Br
	503.9273	0.7	1.4	9.0	207.8	C18 H19 N2 O S Cl Br2
	503.9289	-0.9	-1.8	26.5	810.3	C28 H4 N O3 S Cl2
	503.9290	-1.0	-2.0	20.5	622.2	C22 H7 N3 O5 S Br
	503.9292	-1.2	-2.4	4.0	140.2	C17 H24 O S Cl2 Br2
	503.9268	1.2	2.4	13.5	317.1	C21 H16 N O2 S Br2
	503.9267	1.3	2.6	17.5	615.8	C22 H9 N O5 S Cl3
	503.9294	-1.4	-2.8	22.0	671.8	C25 H7 N2 O2 S Cl3
	503.9295	-1.5	-3.0	16.0	464.6	C19 H10 N4 O4 S Cl Br
	503.9296	-1.6	-3.2	29.5	738.3	C30 H3 N O3 Br
	503.9297	-1.7	-3.4	-0.5	77.6	C14 H27 N S Cl3 Br2
	503.9263	1.7	3.4	4.5	110.8	C17 H23 N Cl3 Br2
	503.9298	-1.8	-3.6	11.0	287.8	C20 H16 O4 Cl3 Br
	503.9261	1.9	3.8	21.0	523.9	C22 H6 N4 O4 Cl Br
	503.9300	-2.0	-4.0	31.0	775.9	C33 H3 Cl3
	503.9260	2.0	4.0	27.0	719.5	C28 H3 N2 O2 Cl3
	503.9301	-2.1	-4.2	25.0	603.3	C27 H6 N2 O2 Cl Br
	503.9258	2.2	4.4	9.0	183.1	C20 H20 O Cl2 Br2
	503.9303	-2.3	-4.6	0.5	61.4	C11 H22 N3 O5 Cl2 Br2
	503.9256	2.4	4.8	25.5	675.0	C25 H3 N3 O5 Br
	503.9255	2.5	5.0	14.0	295.9	C19 H14 N4 O S Br2
	503.9255	2.5	5.0	31.5	847.7	C31 N O3 Cl2
	503.9306	-2.6	-5.2	20.5	464.4	C24 H9 N3 O Cl2 Br
	503.9254	2.6	5.2	18.0	597.2	C20 H7 N4 O4 S Cl3
	503.9308	-2.8	-5.6	15.5	486.0	C21 H12 N O5 S Cl Br
	503.9251	2.9	5.8	0.0	76.1	C12 H24 N2 O3 S Cl2 Br2
	503.9311	-3.1	-6.2	16.0	333.4	C21 H12 N4 Cl3 Br
	503.9249	3.1	6.2	24.5	614.4	C28 H8 N S Cl Br
	503.9249	3.1	6.2	22.5	755.3	C23 H4 N3 O5 S Cl2
	503.9247	3.3	6.5	4.5	148.9	C15 H21 N O4 S Cl Br2
	503.9313	-3.3	-6.5	11.0	338.6	C18 H15 N2 O4 S Cl2 Br
	503.9245	3.5	6.9	9.5	165.2	C18 H18 N3 Cl2 Br2
	503.9244	3.6	7.1	29.0	744.6	C31 H5 O S Br
	503.9316	-3.6	-7.1	31.0	846.2	C31 H2 N2 S Cl2
	503.9317	-3.7	-7.3	25.0	677.8	C25 H5 N4 O2 S Br
	503.9242	3.8	7.5	9.0	251.0	C18 H18 O5 S Br2
	503.9318	-3.8	-7.5	6.5	212.3	C15 H18 N3 O3 S Cl3 Br
	503.9319	-3.9	-7.7	20.0	484.9	C26 H11 O2 Cl2 Br
	503.9240	4.0	7.9	14.0	258.1	C21 H15 N2 O Cl Br2
	503.9320	-4.0	-7.9	14.0	298.4	C20 H14 N2 O4 Br2

Figure S48. HRMS spectrum of **16c**

16c

Elemental Composition Report

Single Mass Analysis (displaying only valid results)
Tolerance = 7.0 PPM / DBE: min = -1.5, max = 50.0
Selected filters: None

Monoisotopic Mass, Even Electron Ions
1048 formula(e) evaluated with 5 results within limits (up to 80 closest results for each mass)
Elements Used:
C: 0-50 H: 0-60 N: 0-5 O: 0-5 Na: 0-1 Cl: 0-3

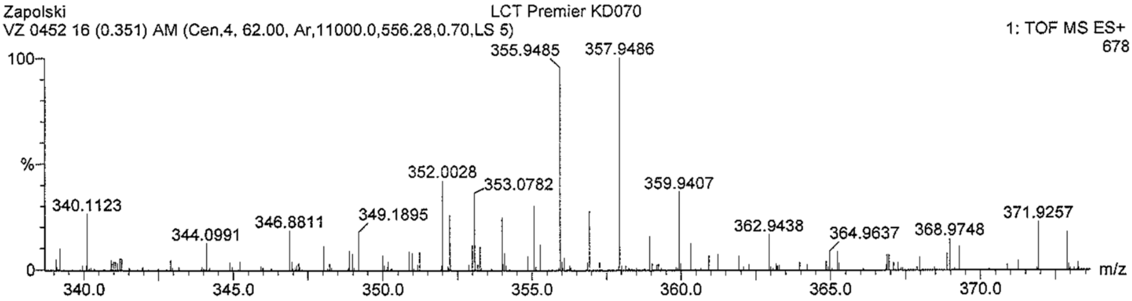

Minimum:				-1.5		
Maximum:		5.0	7.0	50.0		
Mass	Calc. Mass	mDa	PPM	DBE	i-FIT	Formula
355.9485	355.9485	0.0	0.0	8.5	27.4	C10 H6 N5 O2 Na Cl3
	355.9493	-0.8	-2.2	12.5	41.9	C15 H5 N O4 Na Cl2
	355.9475	1.0	2.8	17.5	142.1	C16 N3 O4 Na Cl
	355.9507	-2.2	-6.2	17.5	38.5	C16 H N5 Na Cl2
	355.9509	-2.4	-6.7	11.5	20.5	C12 H5 N5 O2 Cl3

Figure S49. HRMS spectrum of **17c**

17c

Elemental Composition Report **Page 1**

Single Mass Analysis
Tolerance = 10.0 PPM / DBE: min = -1.5, max = 50.0
Element prediction: Off
Number of isotope peaks used for i-FIT = 3

Monoisotopic Mass, Even Electron Ions
1557 formula(e) evaluated with 19 results within limits (up to 50 closest results for each mass)
Elements Used:
C: 0-27 H: 0-38 N: 0-5 O: 0-10 Na: 0-1 Cl: 0-3
Zapolski Q-Tof Premier UPLC-MS 03-Jun-2016 10:49:21
VZ 0293 305 (3.128) AM (Cen,4, 68.00, Ar,10000.0,556.28,0.70,LS 5) 1: TOF MS ES+ 5.25e+002

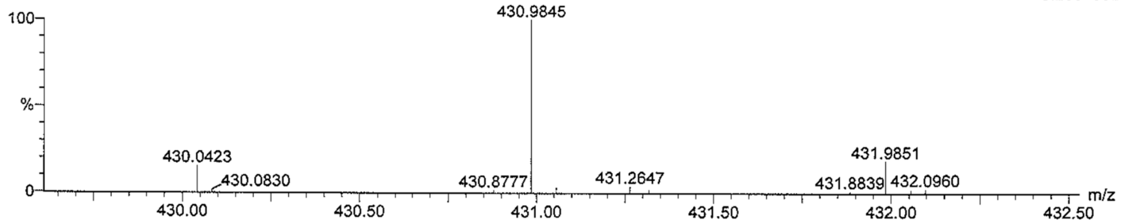

Minimum:				-1.5		
Maximum:		100.0	10.0	50.0		
Mass	Calc. Mass	mDa	PPM	DBE	i-FIT	Formula
430.9845	430.9845	0.0	0.0	12.5	n/a	C17 H11 N4 O2 Na Cl3
	430.9838	0.7	1.6	15.5	n/a	C19 H9 N2 O6 Cl2
	430.9836	0.9	2.1	21.5	n/a	C23 H5 N2 O4 Na Cl
	430.9854	-0.9	-2.1	16.5	n/a	C22 H10 O4 Na Cl2
	430.9856	-1.1	-2.6	10.5	n/a	C18 H14 O6 Cl3
	430.9832	1.3	3.0	7.5	n/a	C16 H15 O6 Na Cl3
	430.9860	-1.5	-3.5	24.5	n/a	C25 H4 N2 O4 Cl
	430.9828	1.7	3.9	24.5	n/a	C25 H3 O8
	430.9867	-2.2	-5.1	21.5	n/a	C23 H6 N4 Na Cl2
	430.9869	-2.4	-5.6	15.5	n/a	C19 H10 N4 O2 Cl3
	430.9819	2.6	6.0	20.5	n/a	C20 H4 N4 O6 Cl
	430.9817	2.8	6.5	26.5	n/a	C24 N4 O4 Na
	430.9816	2.9	6.7	6.5	n/a	C13 H14 N2 O8 Cl3
	430.9876	-3.1	-7.2	17.5	n/a	C17 H4 N4 O9 Na
	430.9814	3.1	7.2	12.5	n/a	C17 H10 N2 O6 Na Cl2
	430.9878	-3.3	-7.7	19.5	n/a	C24 H9 O4 Cl2
	430.9806	3.9	9.0	15.5	n/a	C19 H8 O10 Cl
	430.9886	-4.1	-9.5	16.5	n/a	C22 H11 N2 Na Cl3
	430.9804	4.1	9.5	21.5	n/a	C23 H4 O8 Na

Figure S50. HRMS spectrum of **18a**

18a

Elemental Composition Report

Page 1

Single Mass Analysis (displaying only valid results)

Tolerance = 7.0 PPM / DBE: min = -1.5, max = 50.0
Selected filters: None

Monoisotopic Mass, Even Electron Ions
3702 formula(e) evaluated with 22 results within limits (up to 80 closest results for each mass)
Elements Used:
C: 0-50 H: 0-60 N: 0-5 O: 0-5 Na: 0-1 S: 0-2 Cl: 0-3

Zapolski LCT Premier KD070
VZ 2311 13 (0.299) AM (Cen,4, 73.00, Ar,11000.0,556.28,0.70,LS 5) 1: TOF MS ES+ 2.43e3

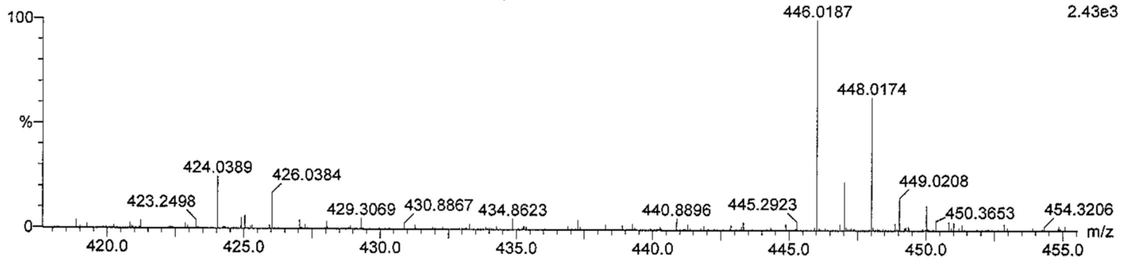

Mass	Calc. Mass	mDa	PPM	DBE	i-FIT	Formula
Minimum:				-1.5		
Maximum:		5.0	7.0	50.0		
446.0187	446.0187	0.0	0.0	16.5	2.6	C20 H11 N5 O2 Na Cl2
	446.0185	0.2	0.4	4.5	162.2	C16 H23 N O3 S2 Cl3
	446.0189	-0.2	-0.4	18.5	81.8	C23 H13 N3 O S2 Cl
	446.0190	-0.3	-0.7	10.5	118.5	C16 H15 N5 O4 Cl3
	446.0183	0.4	0.9	10.5	16.4	C20 H19 N O Na S2 Cl2
	446.0196	-0.9	-2.0	20.5	149.9	C25 H10 N O4 Na Cl
	446.0178	0.9	2.0	25.5	676.3	C26 H5 N3 O4 Na
	446.0199	-1.2	-2.7	2.5	171.0	C11 H20 N5 O4 Na S Cl3
	446.0173	1.4	3.1	18.5	21.1	C25 H14 N O S Cl2
	446.0202	-1.5	-3.4	28.5	679.3	C28 H4 N3 O4
	446.0170	1.7	3.8	23.5	507.6	C24 H8 N5 O S2
	446.0206	-1.9	-4.3	11.5	107.3	C19 H16 N3 O2 Na Cl3
	446.0167	2.0	4.5	9.5	18.7	C17 H18 N3 O3 S2 Cl2
	446.0207	-2.0	-4.5	13.5	19.8	C22 H18 N O S2 Cl2
	446.0166	2.1	4.7	7.5	130.7	C14 H16 N5 O4 Na Cl3
	446.0165	2.2	4.9	15.5	70.9	C21 H14 N3 O Na S2 Cl
	446.0209	-2.2	-4.9	25.5	173.4	C26 H6 N5 Na Cl
	446.0211	-2.4	-5.4	20.5	580.8	C23 H9 N3 O4 Na S
	446.0212	-2.5	-5.6	19.5	7.7	C22 H10 N5 O2 Cl2
	446.0161	2.6	5.8	1.5	175.1	C14 H24 N O3 Na S2 Cl3
	446.0157	3.0	6.7	18.5	487.2	C23 H12 N O5 S2
	446.0218	-3.1	-7.0	29.5	693.6	C31 H5 N O2 Na

Figure S51. HRMS spectrum of **18b**

18c

Elemental Composition Report **Page 1**

Single Mass Analysis
Tolerance = 10.0 PPM / DBE: min = -1.5, max = 50.0
Element prediction: Off
Number of isotope peaks used for i-FIT = 3

Monoisotopic Mass, Even Electron Ions
1572 formula(e) evaluated with 21 results within limits (up to 50 closest results for each mass)
Elements Used:
C: 0-27 H: 0-38 N: 0-5 O: 0-10 Na: 0-1 Cl: 0-3
Zapolski Q-Tof Premier UPLC-MS 03-Jun-2016 10:57:59
VZ 0294 345 (3.537) AM (Cen,4, 90.00, Ar,10000.0,556.28,0.70,LS 5) 1: TOF MS ES+ 7.21e+002

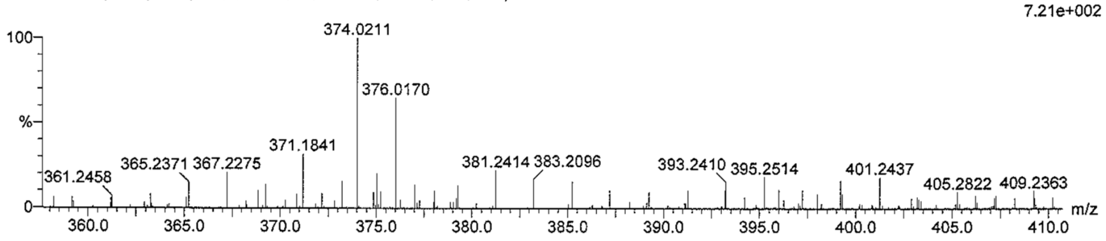

Minimum:				-1.5		
Maximum:		100.0	10.0	50.0		
Mass	Calc. Mass	mDa	PPM	DBE	i-FIT	Formula
374.0211	374.0212	-0.1	-0.3	13.5	0.1	C16 H10 N5 O2 Cl2
	374.0209	0.2	0.5	19.5	49.7	C20 H6 N5 Na Cl
	374.0206	0.5	1.3	5.5	33.7	C13 H16 N3 O2 Na Cl3
	374.0218	-0.7	-1.9	23.5	215.0	C25 H5 N O2 Na
	374.0202	0.9	2.4	22.5	211.2	C22 H4 N3 O4
	374.0220	-0.9	-2.4	17.5	47.0	C21 H9 N O4 Cl
	374.0198	1.3	3.5	8.5	1.4	C15 H14 N O6 Cl2
	374.0196	1.5	4.0	14.5	44.3	C19 H10 N O4 Na Cl
	374.0228	-1.7	-4.5	14.5	0.9	C19 H11 N3 Na Cl2
	374.0230	-1.9	-5.1	8.5	30.3	C15 H15 N3 O2 Cl3
	374.0190	2.1	5.6	4.5	39.9	C10 H15 N5 O4 Cl3
	374.0233	-2.2	-5.9	22.5	54.0	C22 H5 N5 Cl
	374.0187	2.4	6.4	10.5	1.2	C14 H11 N5 O2 Na Cl2
	374.0236	-2.5	-6.7	10.5	214.7	C13 H9 N3 O9 Na
	374.0180	3.1	8.3	13.5	42.0	C16 H9 N3 O6 Cl
	374.0242	-3.1	-8.3	26.5	217.2	C27 H4 N O2
	374.0178	3.3	8.8	19.5	211.4	C20 H5 N3 O4 Na
	374.0176	3.5	9.4	-0.5	47.9	C9 H19 N O8 Cl3
	374.0246	-3.5	-9.4	1.5	17.9	C7 H15 N5 O7 Na Cl2
	374.0246	-3.5	-9.4	9.5	27.5	C18 H16 N Na Cl3
	374.0174	3.7	9.9	5.5	4.5	C13 H15 N O6 Na Cl2

Figure S52. HRMS spectrum of **18c**

18c

Elemental Composition Report **Page 1**

Single Mass Analysis (displaying only valid results)
Tolerance = 7.0 PPM / DBE: min = -1.5, max = 50.0
Selected filters: None

Monoisotopic Mass, Even Electron Ions
3208 formula(e) evaluated with 20 results within limits (up to 80 closest results for each mass)
Elements Used:
C: 0-50 H: 0-60 N: 0-5 O: 0-5 Na: 0-1 S: 0-2 Cl: 0-3
Zapolski LCT Premier KD070
VZ 2500 16 (0.351) AM (Cen,4, 73.00, Ar,11000.0,556.28,0.70,LS 5) 1: TOF MS ES+

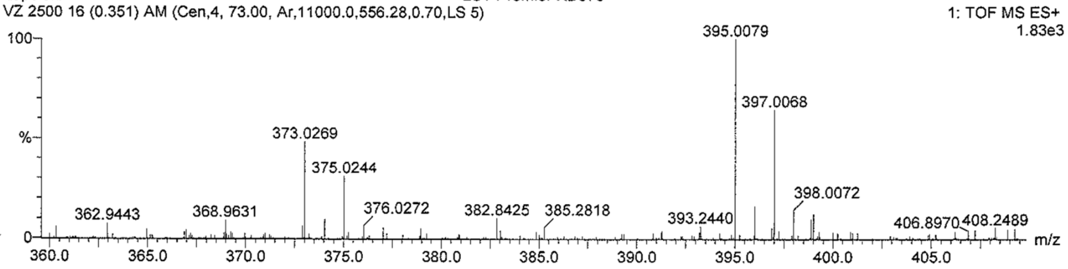

Mass	Calc. Mass	mDa	PPM	DBE	i-FIT	Formula
Minimum:				-1.5		
Maximum:		5.0	7.0	50.0		
395.0079	395.0079	0.0	0.0	13.5	10.4	C17 H10 N4 O2 Na Cl2
	395.0080	-0.1	-0.3	15.5	105.4	C20 H12 N2 O S2 Cl
	395.0081	-0.2	-0.5	7.5	68.3	C13 H14 N4 O4 Cl3
	395.0076	0.3	0.8	1.5	98.0	C13 H22 O3 S2 Cl3
	395.0074	0.5	1.3	7.5	15.0	C17 H18 O Na S2 Cl2
	395.0087	-0.8	-2.0	17.5	162.8	C22 H9 O4 Na Cl
	395.0069	1.0	2.5	22.5	569.1	C23 H4 N2 O4 Na
	395.0090	-1.1	-2.8	-0.5	102.0	C8 H19 N4 O4 Na S Cl3
	395.0093	-1.4	-3.5	25.5	580.6	C25 H3 N2 O4
	395.0064	1.5	3.8	15.5	38.3	C22 H13 O S Cl2
	395.0097	-1.8	-4.6	8.5	64.3	C16 H15 N2 O2 Na Cl3
	395.0061	1.8	4.6	20.5	445.7	C21 H7 N4 O S2
	395.0098	-1.9	-4.8	10.5	24.4	C19 H17 O S2 Cl2
	395.0100	-2.1	-5.3	22.5	191.6	C23 H5 N4 Na Cl
	395.0058	2.1	5.3	6.5	9.6	C14 H17 N2 O3 S2 Cl2
	395.0057	2.2	5.6	4.5	75.1	C11 H15 N4 O4 Na Cl3
	395.0102	-2.3	-5.8	17.5	489.0	C20 H8 N2 O4 Na S
	395.0056	2.3	5.8	12.5	87.7	C18 H13 N2 O Na S2 Cl
	395.0103	-2.4	-6.1	16.5	21.9	C19 H9 N4 O2 Cl2
	395.0052	2.7	6.8	-1.5	105.4	C11 H23 O3 Na S2 Cl3

Figure S53. X-Ray - Supplementary Material for **3a**

checkCIF/PLATON report

You have not supplied any structure factors. As a result the full set of tests cannot be run.

THIS REPORT IS FOR GUIDANCE ONLY. IF USED AS PART OF A REVIEW PROCEDURE FOR PUBLICATION, IT SHOULD NOT REPLACE THE EXPERTISE OF AN EXPERIENCED CRYSTALLOGRAPHIC REFEREE.

No syntax errors found.   CIF dictionary   Interpreting this report

Datablock: 3a

Bond precision: C-C = 0.0086 A   Wavelength=0.71069

Cell:	a=7.6849(13)	b=8.1888(15)	c=12.236(2)
	alpha=77.658(14)	beta=76.198(13)	gamma=68.326(13)
Temperature:	223 K		

	Calculated	Reported
Volume	688.1(2)	688.2(2)
Space group	P -1	P-1
Hall group	-P 1	?
Moiety formula	C8 H7 Cl4 N O4 S	C8 H7 Cl4 N O4 S
Sum formula	C8 H7 Cl4 N O4 S	C8 H7 Cl4 N O4 S
Mr	355.01	355.01
Dx,g cm-3	1.713	1.713
Z	2	2
Mu (mm-1)	1.014	1.014
F000	356.0	356.0
F000'	357.56	
h,k,lmax	9,9,14	9,9,14
Nref	2611	2558
Tmin,Tmax	0.760,0.776	0.760,0.776
Tmin'	0.745	

Correction method= # Reported T Limits: Tmin=0.760 Tmax=0.776
AbsCorr = NUMERICAL

Data completeness= 0.980   Theta(max)= 25.680

R(reflections)= 0.0568(2334)   wR2(reflections)= 0.1564(2558)

S = 1.043   Npar= 191

Figure S54. X-Ray - Supplementary Material for **3a**

The following ALERTS were generated. Each ALERT has the format
test-name_ALERT_alert-type_alert-level.
Click on the hyperlinks for more details of the test.

Alert level B

```
PLAT230_ALERT_2_B Hirshfeld Test Diff for    Cl12      --C11       .       37.8 s.u.
PLAT230_ALERT_2_B Hirshfeld Test Diff for    Cl16      --C15       .       39.2 s.u.
PLAT368_ALERT_2_B Short  C(sp2)-C(sp2) Bond  C11       - C15       .       1.08 Ang.
```

Alert level C

```
ABSTY02_ALERT_1_C  An _exptl_absorpt_correction_type has been given without
            a literature citation. This should be contained in the
            _exptl_absorpt_process_details field.
            Absorption correction given as numerical
PLAT213_ALERT_2_C Atom C11             has ADP max/min Ratio .....        3.2 prolat
PLAT213_ALERT_2_C Atom C15             has ADP max/min Ratio .....        3.1 prolat
PLAT220_ALERT_2_C NonSolvent    Resd 1  C    Ueq(max)/Ueq(min) Range      3.5 Ratio
PLAT241_ALERT_2_C High   'MainMol' Ueq as Compared to Neighbors of        C11 Check
PLAT241_ALERT_2_C High   'MainMol' Ueq as Compared to Neighbors of        C15 Check
PLAT242_ALERT_2_C Low    'MainMol' Ueq as Compared to Neighbors of        C10 Check
PLAT250_ALERT_2_C Large U3/U1 Ratio for Average U(i,j) Tensor ....        2.3 Note
PLAT336_ALERT_2_C Long Bond Distance for ..... C11      -Cl12           1.928 Ang.
PLAT336_ALERT_2_C Long Bond Distance for ..... C15      -Cl16           1.903 Ang.
PLAT340_ALERT_3_C Low Bond Precision on  C-C Bonds ...............     0.0086 Ang.
PLAT790_ALERT_4_C Centre of Gravity not Within Unit Cell: Resd.  #          1 Note
              C8 H7 Cl4 N O4 S
```

Alert level G

```
PLAT005_ALERT_5_G No Embedded Refinement Details Found  in the CIF     Please Do !
PLAT066_ALERT_1_G Predicted and Reported Tmin&Tmax Range Identical         ? Check
PLAT431_ALERT_2_G Short Inter HL..A Contact  Cl12      ..O17      .       3.08 Ang.
                                          1-x,-y,-z  =        2_655 Check
PLAT899_ALERT_4_G SHELXL97  is Deprecated and Succeeded by SHELXL-     2019/2 Note
```

 0 **ALERT level A** = Most likely a serious problem - resolve or explain
 3 **ALERT level B** = A potentially serious problem, consider carefully
12 **ALERT level C** = Check. Ensure it is not caused by an omission or oversight
 4 **ALERT level G** = General information/check it is not something unexpected

 2 ALERT type 1 CIF construction/syntax error, inconsistent or missing data
13 ALERT type 2 Indicator that the structure model may be wrong or deficient
 1 ALERT type 3 Indicator that the structure quality may be low
 2 ALERT type 4 Improvement, methodology, query or suggestion
 1 ALERT type 5 Informative message, check

Figure S55. X-Ray - Supplementary Material for **3a**

It is advisable to attempt to resolve as many as possible of the alerts in all categories. Often the minor alerts point to easily fixed oversights, errors and omissions in your CIF or refinement strategy, so attention to these fine details can be worthwhile. In order to resolve some of the more serious problems it may be necessary to carry out additional measurements or structure refinements. However, the purpose of your study may justify the reported deviations and the more serious of these should normally be commented upon in the discussion or experimental section of a paper or in the "special_details" fields of the CIF. checkCIF was carefully designed to identify outliers and unusual parameters, but every test has its limitations and alerts that are not important in a particular case may appear. Conversely, the absence of alerts does not guarantee there are no aspects of the results needing attention. It is up to the individual to critically assess their own results and, if necessary, seek expert advice.

Publication of your CIF in IUCr journals

A basic structural check has been run on your CIF. These basic checks will be run on all CIFs submitted for publication in IUCr journals (*Acta Crystallographica*, *Journal of Applied Crystallography*, *Journal of Synchrotron Radiation*); however, if you intend to submit to *Acta Crystallographica Section C* or *E* or *IUCrData*, you should make sure that full publication checks are run on the final version of your CIF prior to submission.

Publication of your CIF in other journals

Please refer to the *Notes for Authors* of the relevant journal for any special instructions relating to CIF submission.

PLATON version of 28/11/2022; check.def file version of 28/11/2022

Figure S56. X-Ray - Supplementary Material for **3a**

Datablock 3a - ellipsoid plot

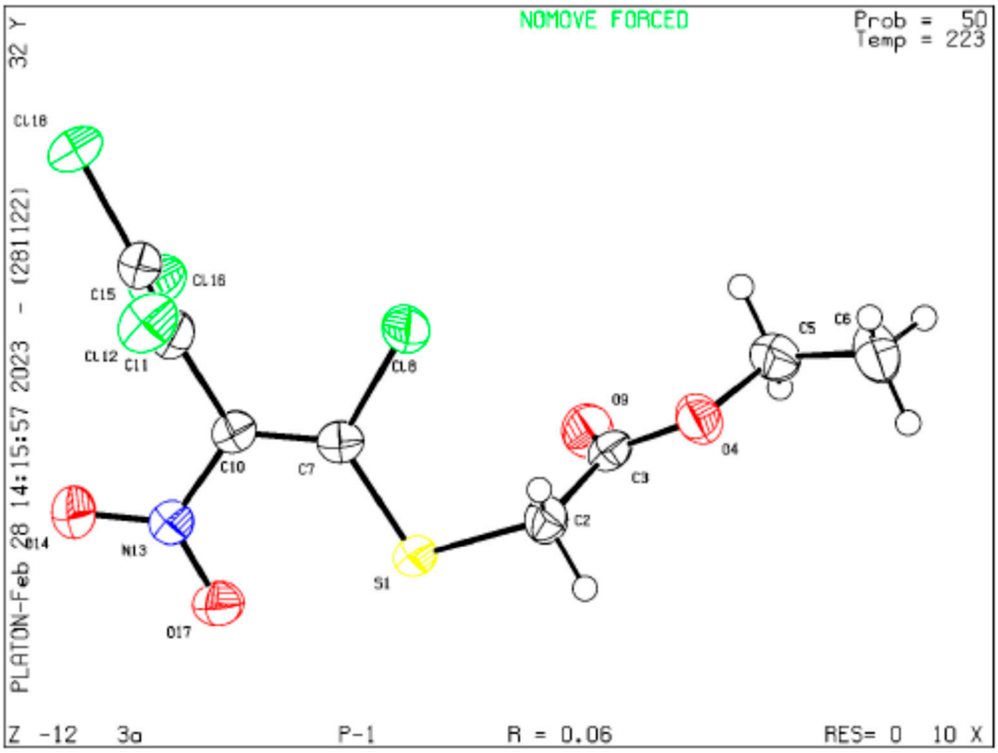

Figure S57. X-Ray - Supplementary Material for **5a**

checkCIF/PLATON report

You have not supplied any structure factors. As a result the full set of tests cannot be run.

THIS REPORT IS FOR GUIDANCE ONLY. IF USED AS PART OF A REVIEW PROCEDURE FOR PUBLICATION, IT SHOULD NOT REPLACE THE EXPERTISE OF AN EXPERIENCED CRYSTALLOGRAPHIC REFEREE.

No syntax errors found. CIF dictionary Interpreting this report

Datablock: 5a

```
Bond precision:    C-C = 0.0069 A           Wavelength=0.71073

Cell:             a=11.5036(16)     b=11.5202(15)      c=14.139(2)
                  alpha=90          beta=101.168(12)   gamma=90
Temperature:      223 K

                  Calculated                  Reported
Volume            1838.3(4)                   1838.3(5)
Space group       P 21/n                      P21/n
Hall group        -P 2yn                      ?
Moiety formula    C18 H14 Cl2 N2 O4 S         C18 H14 Cl2 N2 O4 S
Sum formula       C18 H14 Cl2 N2 O4 S         C18 H14 Cl2 N2 O4 S
Mr                425.27                      425.27
Dx,g cm-3         1.537                       1.537
Z                 4                           4
Mu (mm-1)         0.495                       0.495
F000              872.0                       872.0
F000'             874.03
h,k,lmax          13,13,16                    13,13,16
Nref              3255                        3257
Tmin,Tmax         0.871,0.897                 0.871,0.897
Tmin'             0.871

Correction method= # Reported T Limits: Tmin=0.871 Tmax=0.897
AbsCorr = NUMERICAL

Data completeness= 1.001           Theta(max)= 25.020

R(reflections)= 0.0642( 2351)                  wR2(reflections)=
                                               0.1431( 3257)
S = 1.032                  Npar= 295
```

Figure S58. X-Ray - Supplementary Material for **5a**

```
The following ALERTS were generated. Each ALERT has the format
       test-name_ALERT_alert-type_alert-level.
Click on the hyperlinks for more details of the test.
```

Alert level B

```
PLAT201_ALERT_2_B Isotropic non-H Atoms in Main Residue(s) .......          1 Report
              S15
```

Alert level C

```
ABSTY02_ALERT_1_C  An _exptl_absorpt_correction_type has been given without
            a literature citation. This should be contained in the
            _exptl_absorpt_process_details field.
            Absorption correction given as numerical
PLAT222_ALERT_3_C NonSolvent Resd 1  H   Uiso(max)/Uiso(min) Range       5.9 Ratio
PLAT245_ALERT_2_C U(iso) H19A      Smaller than U(eq) C19       by      0.026 Ang**2
PLAT340_ALERT_3_C Low Bond Precision on  C-C Bonds ..............    0.00688 Ang.
```

Alert level G

```
PLAT005_ALERT_5_G No Embedded Refinement Details Found  in the CIF     Please Do !
PLAT066_ALERT_1_G Predicted and Reported Tmin&Tmax Range Identical          ? Check
PLAT432_ALERT_2_G Short Inter X...Y Contact  O26       ..C2       .       3.01 Ang.
                                       1-x,1-y,-z  =      3_665 Check
PLAT899_ALERT_4_G SHELXL97  is Deprecated and Succeeded by SHELXL-     2019/2 Note
```

```
   0 ALERT level A - Most likely a serious problem - resolve or explain
   1 ALERT level B - A potentially serious problem, consider carefully
   4 ALERT level C - Check. Ensure it is not caused by an omission or oversight
   4 ALERT level G - General information/check it is not something unexpected

   2 ALERT type 1 CIF construction/syntax error, inconsistent or missing data
   3 ALERT type 2 Indicator that the structure model may be wrong or deficient
   2 ALERT type 3 Indicator that the structure quality may be low
   1 ALERT type 4 Improvement, methodology, query or suggestion
   1 ALERT type 5 Informative message, check
```

Figure S59. X-Ray - Supplementary Material for **5a**

It is advisable to attempt to resolve as many as possible of the alerts in all categories. Often the minor alerts point to easily fixed oversights, errors and omissions in your CIF or refinement strategy, so attention to these fine details can be worthwhile. In order to resolve some of the more serious problems it may be necessary to carry out additional measurements or structure refinements. However, the purpose of your study may justify the reported deviations and the more serious of these should normally be commented upon in the discussion or experimental section of a paper or in the "special_details" fields of the CIF. checkCIF was carefully designed to identify outliers and unusual parameters, but every test has its limitations and alerts that are not important in a particular case may appear. Conversely, the absence of alerts does not guarantee there are no aspects of the results needing attention. It is up to the individual to critically assess their own results and, if necessary, seek expert advice.

Publication of your CIF in IUCr journals

A basic structural check has been run on your CIF. These basic checks will be run on all CIFs submitted for publication in IUCr journals (*Acta Crystallographica*, *Journal of Applied Crystallography*, *Journal of Synchrotron Radiation*); however, if you intend to submit to *Acta Crystallographica Section C* or *E* or *IUCrData*, you should make sure that full publication checks are run on the final version of your CIF prior to submission.

Publication of your CIF in other journals

Please refer to the *Notes for Authors* of the relevant journal for any special instructions relating to CIF submission.

PLATON version of 28/11/2022; check.def file version of 28/11/2022

Figure S60. X-Ray - Supplementary Material for **5a**

Datablock 5a - ellipsoid plot

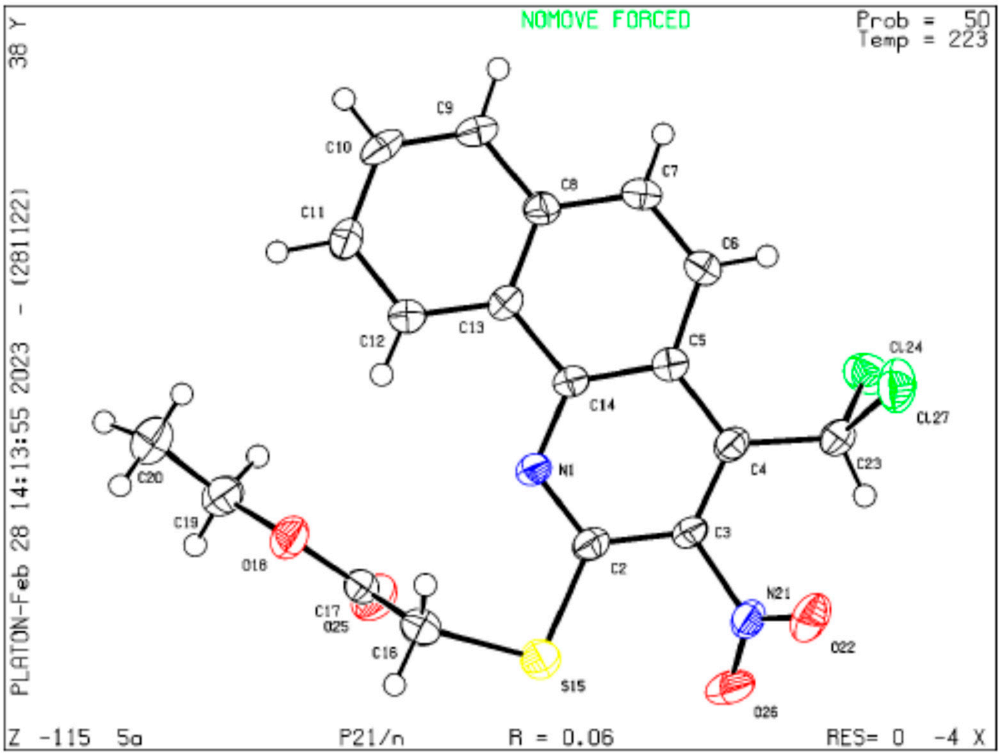

Figure S61. X-Ray - Supplementary Material for **11b**

checkCIF/PLATON report

You have not supplied any structure factors. As a result the full set of tests cannot be run.

THIS REPORT IS FOR GUIDANCE ONLY. IF USED AS PART OF A REVIEW PROCEDURE FOR PUBLICATION, IT SHOULD NOT REPLACE THE EXPERTISE OF AN EXPERIENCED CRYSTALLOGRAPHIC REFEREE.

No syntax errors found. CIF dictionary Interpreting this report

Datablock: 11b

```
Bond precision:    C-C = 0.0035 A          Wavelength=0.71073

Cell:              a=7.1031(9)     b=22.053(2)         c=11.3620(13)
                   alpha=90        beta=99.704(10)     gamma=90
Temperature:       223 K

                 Calculated                   Reported
Volume           1754.3(3)                    1754.3(4)
Space group      P 21/c                       P21/c
Hall group       -P 2ybc                      ?
Moiety formula   C18 H15 Cl2 N3 O3            C18 H15 Cl2 N3 O3
Sum formula      C18 H15 Cl2 N3 O3            C18 H15 Cl2 N3 O3
Mr               392.23                       392.23
Dx,g cm-3        1.485                        1.485
Z                4                            4
Mu (mm-1)        0.394                        0.394
F000             808.0                        808.0
F000'            809.50
h,k,lmax         8,26,13                      8,26,13
Nref             3331                         3328
Tmin,Tmax        0.903,0.924                  0.903,0.924
Tmin'            0.903

Correction method= # Reported T Limits: Tmin=0.903 Tmax=0.924
AbsCorr = NUMERICAL

Data completeness= 0.999           Theta(max)= 25.680

                                                     wR2(reflections)=
R(reflections)= 0.0528( 2792)                        0.1456( 3328)

S = 1.075                Npar= 295
```

Figure S62. X-Ray - Supplementary Material for **11b**

```
The following ALERTS were generated. Each ALERT has the format
       test-name_ALERT_alert-type_alert-level.
Click on the hyperlinks for more details of the test.
```

Alert level C

```
ABSTY02_ALERT_1_C  An _exptl_absorpt_correction_type has been given without
            a literature citation. This should be contained in the
            _exptl_absorpt_process_details field.
            Absorption correction given as numerical
RINTA01_ALERT_3_C  The value of Rint is greater than 0.12
            Rint given    0.151
PLAT790_ALERT_4_C Centre of Gravity not Within Unit Cell: Resd.  #          1 Note
              C18 H15 Cl2 N3 O3
```

Alert level G

```
PLAT005_ALERT_5_G No Embedded Refinement Details Found  in the CIF     Please Do !
PLAT020_ALERT_3_G The Value of Rint is Greater Than 0.12 .........      0.151 Report
PLAT066_ALERT_1_G Predicted and Reported Tmin&Tmax Range Identical          ? Check
PLAT333_ALERT_2_G Large Aver C6-Ring C-C Dist C9        -C13      .       1.42 Ang.
PLAT398_ALERT_2_G Deviating  C-O-C   Angle From 120 for O1        .      109.4 Degree
PLAT432_ALERT_2_G Short Inter X...Y Contact  Cl22      ..C13      .       3.24 Ang.
                                          1+x,y,z  =      1_655 Check
PLAT899_ALERT_4_G SHELXL97  is Deprecated and Succeeded by SHELXL-     2019/2 Note
```

```
   0 ALERT level A - Most likely a serious problem - resolve or explain
   0 ALERT level B - A potentially serious problem, consider carefully
   3 ALERT level C - Check. Ensure it is not caused by an omission or oversight
   7 ALERT level G - General information/check it is not something unexpected

   2 ALERT type 1 CIF construction/syntax error, inconsistent or missing data
   3 ALERT type 2 Indicator that the structure model may be wrong or deficient
   2 ALERT type 3 Indicator that the structure quality may be low
   2 ALERT type 4 Improvement, methodology, query or suggestion
   1 ALERT type 5 Informative message, check
```

Figure S63. X-Ray - Supplementary Material for **11b**

It is advisable to attempt to resolve as many as possible of the alerts in all categories. Often the minor alerts point to easily fixed oversights, errors and omissions in your CIF or refinement strategy, so attention to these fine details can be worthwhile. In order to resolve some of the more serious problems it may be necessary to carry out additional measurements or structure refinements. However, the purpose of your study may justify the reported deviations and the more serious of these should normally be commented upon in the discussion or experimental section of a paper or in the "special_details" fields of the CIF. checkCIF was carefully designed to identify outliers and unusual parameters, but every test has its limitations and alerts that are not important in a particular case may appear. Conversely, the absence of alerts does not guarantee there are no aspects of the results needing attention. It is up to the individual to critically assess their own results and, if necessary, seek expert advice.

Publication of your CIF in IUCr journals

A basic structural check has been run on your CIF. These basic checks will be run on all CIFs submitted for publication in IUCr journals (*Acta Crystallographica*, *Journal of Applied Crystallography*, *Journal of Synchrotron Radiation*); however, if you intend to submit to *Acta Crystallographica Section C* or *E* or *IUCrData*, you should make sure that full publication checks are run on the final version of your CIF prior to submission.

Publication of your CIF in other journals

Please refer to the *Notes for Authors* of the relevant journal for any special instructions relating to CIF submission.

PLATON version of 28/11/2022; check.def file version of 28/11/2022

Figure S64. X-Ray - Supplementary Material for **11b**

Datablock 11b - ellipsoid plot

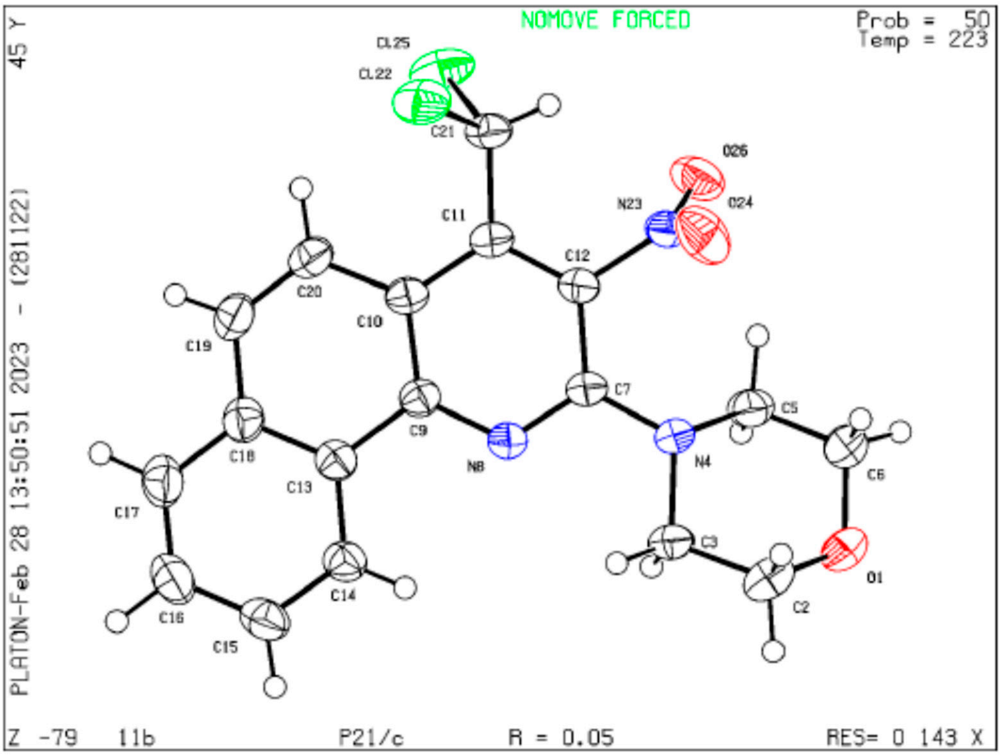

Supplement: Supplementary file 1 [file molecules-28-02479-s001.zip › molecules-2227845-supplementary.pdf]
